# Large language model as clinical decision support system augments medication safety in 16 clinical specialties

**DOI:** 10.1016/j.xcrm.2025.102323

**Published:** 2025-09-24

**Authors:** Jasmine Chiat Ling Ong, Liyuan Jin, Kabilan Elangovan, Gilbert Yong San Lim, Daniel Yan Zheng Lim, Gerald Gui Ren Sng, Yu He Ke, Joshua Yi Min Tung, Ryan Jian Zhong, Christopher Ming Yao Koh, Keane Zhi Hao Lee, Xiang Chen, Jack Kian Ch’ng, Aung Than, Ken Junyang Goh, Chuan Poh Lim, Tat Ming Ng, Nan Liu, Daniel Shu Wei Ting

**Affiliations:** 1 Division of Pharmacy, Singapore General Hospital, Singapore, Singapore; 2 Duke-NUS Medical School, Singapore, Singapore; 3 Singapore National Eye Centre, Singapore Eye Research Institute, Singapore, Singapore; 4 Singapore Health Services, Artificial Intelligence Office, Singapore, Singapore; 5 Department of Gastroenterology, Singapore General Hospital, Singapore, Singapore; 6 Data Science and Artificial Intelligence Lab, Singapore General Hospital, Singapore, Singapore; 7 Department of Anesthesiology, Singapore General Hospital, Singapore, Singapore; 8 Department of Endocrinology, Singapore General Hospital, Singapore, Singapore; 9 Department of Urology, Singapore General Hospital, Singapore, Singapore; 10 Department of Vascular Surgery, Singapore General Hospital, Singapore, Singapore; 11 Department of Internal Medicine, Singapore General Hospital, Singapore, Singapore; 12 Department of Respiratory and Critical Care Medicine, Singapore General Hospital, Singapore, Singapore; 13 Division of Pharmacy, Tan Tock Seng Hospital, Singapore, Singapore

**Keywords:** large language models, medication safety, pharmacy, clinical-AI collaboration, co-pilot

## Abstract

Large language models (LLMs) have emerged as tools to support healthcare delivery, from automating tasks to aiding clinical decision-making. This study evaluated LLMs as alternative to rule-based alert systems, focusing on their ability to identify prescribing errors. This was designed as a prospective, cross-over, open-label study involving 91 error scenarios based on 40 clinical vignettes across 16 medical and surgical specialties. We developed and validated five LLM models using a retrieval-augmented generation framework. The best-performing model evaluated three different implementation strategies: LLM-based clinical decision support system (CDSS) alone, pharmacist plus LLM-based CDSS (co-pilot), and pharmacist alone. The co-pilot arm demonstrated the best performance with an accuracy of 61% (precision 0.57, recall 0.61, and F1 0.59). In detecting errors posing serious harm, the co-pilot mode increased accuracy by 1.5-fold over the pharmacist alone. Effective LLM integration for complex tasks like medication chart reviews can enhance healthcare professional performance, improving patient safety.

## Highlights

• Evaluated LLMs in identifying drug-related problems across complex cases
• Developed and validated RAG-LLM to enhance reasoning in clinical decision support tasks
• Demonstrated varying LLM accuracy in medication safety-related reasoning
• LLM use in real-world clinical practice

## Graphical abstract

**Figure:**
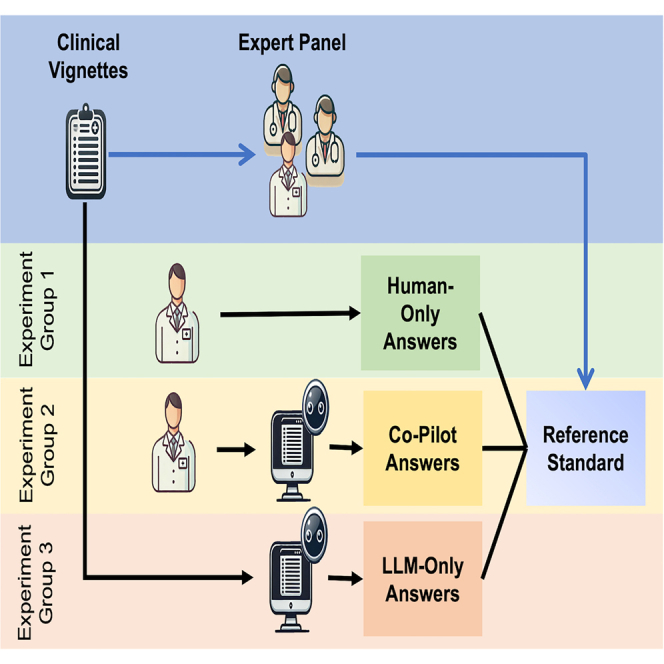

## Teaser

Ong et al. explored how large language models (LLMs) support safe prescribing by detecting drug-related problems across clinical scenarios. The study demonstrated that LLMs augmented performance of pharmacists and improved overall accuracy of medication chart review. However, limitations in clinical generalizability, bias, and prompt design must be addressed before routine use.

## Introduction

Medical errors remain a formidable challenge for healthcare institutions all around the world and are the third leading cause of mortality in the United States. Medication-related errors account for an average of 21% of hospital readmissions, with 69% of these admissions considered preventable.^1^^,^^2^ Medication errors can potentially result in prolonged hospitalization stay and elevated risk for morbidity and mortality as well as an increase in healthcare spending.^3^ This translates to high economic burden of medication errors, amounting up to USD$40 billion in the United States and £750 million in England per year.^3^^,^^4^ In an acute care setting, medication errors can occur at any stage of the medication use process: medication prescribing, dispensing, and administration and patient monitoring. A vast majority of errors occur at the prescribing stage, accounting for 70% of errors that result in adverse patient events.^5^ Halting errors at this stage is critical in preventing perpetuation of the error downstream and eventually reaching the patient.

Clinical decision support systems (CDSSs) have become a cornerstone of modern healthcare systems as a direct aid to clinical decision-making. A CDSS is intended to improve healthcare delivery by enhancing medical decisions with targeted clinical knowledge, patient information, and other health information.^6^ CDSSs have demonstrated utility in reducing prescribing errors or adverse events when integrated with electronic health records and computerized provider order entry systems.^7^ In particular categories of prescribing errors such as drug-drug interactions, the real-world implementation of CDSS was effective in reducing the incidence of errors.^8^ However, a vast majority of current systems are rule based, resulting in the generation of voluminous and clinically irrelevant alerts to users.^9^ Over time, safety alerts are ignored and overwritten by physicians in as high as 95%. “Alert fatigue,” coupled with heavy patient load and high cognitive burden from the need to process a massive amount of patient health information, poses barrier to effective adoption and utilization of CDSS systems.^10^^,^^11^^,^^12^ Greater personalization of alerts, more intuitive interfaces, and lesser emphasis on disruptive alerts show promise in improving current state of CDSS.

The growing capabilities of large language models (LLMs) in medical tasks are becoming more apparent. LLMs are advancing in handling such tasks, particularly those that do not require extensive specialized expertise.^13^^,^^14^ This includes simplifying administrative duties like composing medical letters, creating summaries upon patient discharge using information from electronic health records (EHRs) to semi-autonomous decision-making support for managing operating theaters.^15^^,^^16^ Finally, LLM-powered healthcare chatbots are capable of providing patients and health professionals with highly professional-sounding, accurate, and personalized responses to medical queries.^17^^,^^18^ Healthcare systems are facing critical shortage of healthcare professionals compounded by a mounting issue of healthcare professional burnout.^19^^,^^20^^,^^21^ LLM-powered solutions are well poised to improve operational efficiency and standards of patient care when trained with the right data, robustly evaluated in the clinical setting with a deployment strategy armed with appropriate safety measures.

Prior published studies have developed LLM-based tools to support various clinical applications and domains.^22^^,^^23^^,^^24^ Generative AI tools, such as LLMs, demonstrate potential to reduce medication-related harm when integrated into the medication use process as clinical decision support tools. LLMs were designed to perform specific tasks such as identifying inappropriate benzodiazepine prescriptions, classifying adverse drug reactions to chemotherapy, and predicting potential drug-drug interactions. The use of LLMs as an innovative substitute to current rules-based CDSS for medication review, however, has not been described. In this study, we propose a modified clinical model of care, leveraging upon LLMs as a tool to improve safety of medication use in acute care settings. The objective of this study was to evaluate the performance of LLM-based tools in correctly identifying prescribing errors and provision of clinically aligned recommendations to rectify identified errors across different medical disciplines. This has implications for the potential integration of such tools in healthcare and attendant improvements in patient safety and quality of care.

## Results

Six pharmacists of varying levels of work experience participated in this study. Four participants were junior pharmacists with post-licensure practice experience of between 2 and 5 years, while 2 participants were senor pharmacists with >10 years of post-licensure practice experience. The case vignettes are representative of complex clinical case scenarios with multiple co-morbidities and problem lists (Table 1). The expert panel determined that 31.9% (29 out of 91) error scenarios were capable of causing serious harm and 52.7% (48 out of 91) capable of inflicting moderate harm while the remaining 15.4% (14 out of 91) were rated as capable of causing minor or no harm. The three most common drug-related problems (DRPs) in the case scenarios were inappropriate dosage regimen that arose due the need for dose, frequency or duration adjustment; adverse drug reactions requiring change in medication or reversal agents (this also includes prescribing medications that are contraindicated based on patient profile), and significant drug-drug interactions requiring change in medication or therapeutic drug monitoring. The median unique medications across anatomical therapeutic chemical classes was 12 (IQR 5–16) per case vignette, suggesting that our constructed cases were complex and demonstrative of patients seen at a tertiary healthcare institution.

**Table 1:** Demographics of patient case vignettes

Variable	Case vignette subjects, no. (%), (*N* = 40)	
Age, median (interquartile range, IQR) [range], years	65.5	(58.5, 72), [17–86]

**Gender**		

*Female*	15	(37.5)
*Male*	25	(62.5)

**Admitting discipline**		

*Medical*	27	(67.5)
*Surgical*	13	(32.5)
Charlson’s comorbidity index, median (IQR) [range]	3	(2, 5), [0–10]

**Co-morbidities**		

*Myocardial infarction*	14	(35)
*Congestive heart failure*	6	(15)
*Peripheral vascular disease*	2	(5)
*CVA or TIA*	5	(12.5)
*Dementia*	2	(5)
*COPD*	1	(2.5)
*Peptic ulcer disease*	1	(2.5)
*Severe liver disease*	6	(15)
*Complicated diabetes mellitus*	19	(47.5)
*Moderate to severe CKD*	16	(40)
*Solid cancer*	6	(15)
*Hematological cancer*	1	(2.5)

**Medication reconciliation performed**		

*Yes*	33	(82.5)
*No*	7	(17.5)
No. of medication orders, median (IQR) [range]	13	(10, 15), [5–22]
MCRI score, median (IQR) [range]	40	(30, 52), [14–83]

### Comparative performance of native and RAG-LLMs

**Figure 1:**
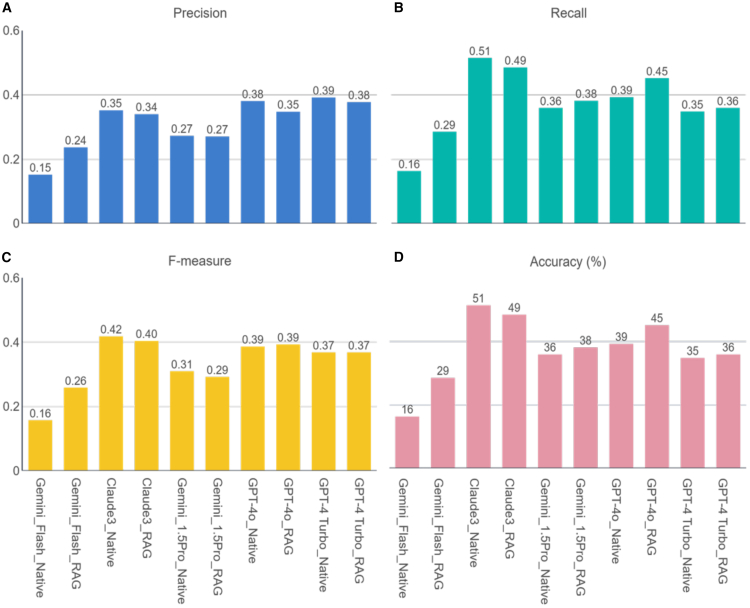
Comparative performance of respective native and RAG-LLMs Performance of different LLMs in terms of (A) precision, (B) recall, (C) F-measure, and (D) accuracy.

We evaluated outputs from native LLMs and retrieval augmented generation (RAG)-LLMs. Mean performance of all models were presented in Figure 1. Of the native models, Gemini Flash scored the lowest while Claude 3.5 Sonnet performed the best on measures of recall (0.16 vs. 0.51), precision (0.15 vs. 0.35), F1 (0.16 vs. 0.42), and overall accuracy (16% vs. 51%). Applying a RAG framework improved accuracy and recall of all models except for Claude 3.5 Sonnet. When compared against the native model, the RAG model demonstrated difference in accuracy of −2% (51% vs. 49%), recall of −0.02 (0.51 vs. 0.49), precision of −0.01 (0.35 vs. 0.34), and F-measure of −0.02 (0.42 vs. 0.40). A secondary analysis using a reasoning model, OpenAI’s o4-mini, was performed with results reported in Table S3.

**Figure 2:**
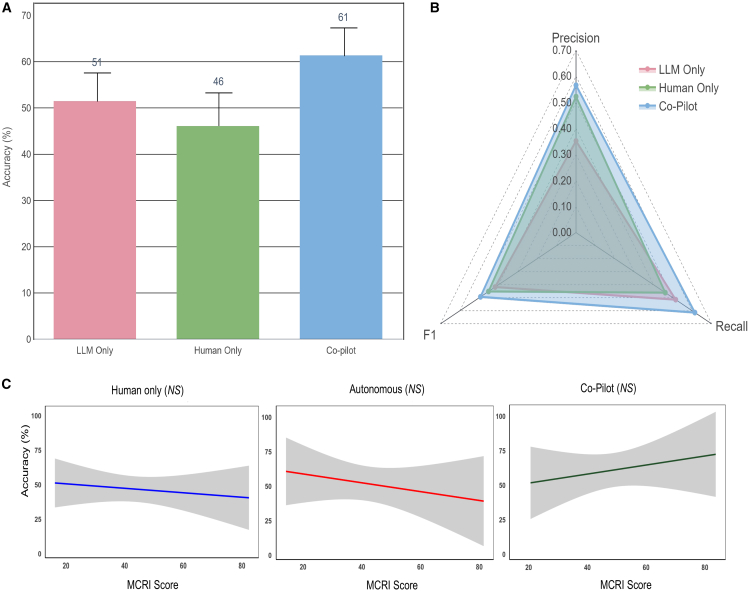
Performance of different modalities of implementation (A) Chart showing comparative accuracy of different modes. (B) Spider diagram showing relative precision, recall, and F1 scores of different modes. (C) No correlation between MCRI score and accuracy in identifying medication-related problems. Higher MCRI scores suggest greater complexity of cases. The three plots are presented as correlation coefficient −0.0777 (95% CI: −0.3272 to 0.182) for human only, −0.1389 (95% CI: −0.4353 to 0.1848), and 0.1456 (95% CI: −0.2081 to 0.4657). The solid line represents respective regression lines. The translucent band around the regression line represents the 95% CI. NS indicates no statistical significance (*p* > 0.05).

Reproducibility as evaluated using BERTscore (bidirectional encoder representations from transformer)ML, cosine similarity, and BLEU (bilingual evaluation understudy) score demonstrated high degree of reproducibility across LLM responses when prompted repeatedly. Claude 3.5 Sonnet demonstrated high degree of semantic reproducibility, scoring an average of 0.84, 0.947, and 0.570 for BERT score, cosine similarity, and BLEU score, respectively.

### Comparative performance of different modes of delivery

The Claude 3.5 Sonnet (native) model was the adopted model in LLM-CDSS due to the best overall performance during validation. There was no statistically significant differences in mean accuracy between different modes of care: co-pilot (pharmacist with LLM-CDSS), human only (pharmacist without LLM-CDSS), and LLM only (*p = 0.38*) (Figure 2A). The mean accuracy in identifying DRPs increased by 32.6% in co-pilot mode when compared against human alone (61%, SD 17.7 vs. 46%, SD 21.1). Precision, recall, and F1 score were all higher in co-pilot mode when compared against human alone (0.57 vs. 0.52, 0.61 vs. 0.46, and 0.49 vs. 0.45) (Figure 2B). Performance under various modes was not significantly correlated with increasing case complexity as measured with the medication regimen complexity index (MCRI) score (Figure 2C).

**Figure 3:**
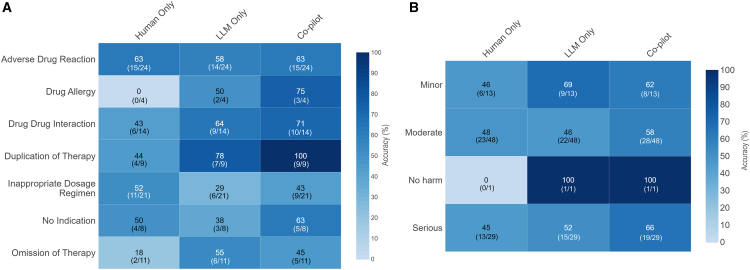
Performance across different categories of DRPs and different severities of potential harm (A) Heatmap showing mean accuracy of different modes in various categories of DRP. (B) Heatmap showing mean accuracy of different modes for DRPs of varying severity of harm. Data are represented as mean ± SD.

In terms of DRP categories (Figure 3), co-pilot mode showed higher accuracy of DRP identification when compared against human alone across all categories except for inappropriate dosage regimen. Accuracy was seen to decline in co-pilot mode when an inappropriate dosing regimen was encountered in the clinical vignettes (52% vs. 43%). LLMs alone were capable of catching 52% of serious errors, while that improved with co-pilot mode to 66%.

**Figure 4:**
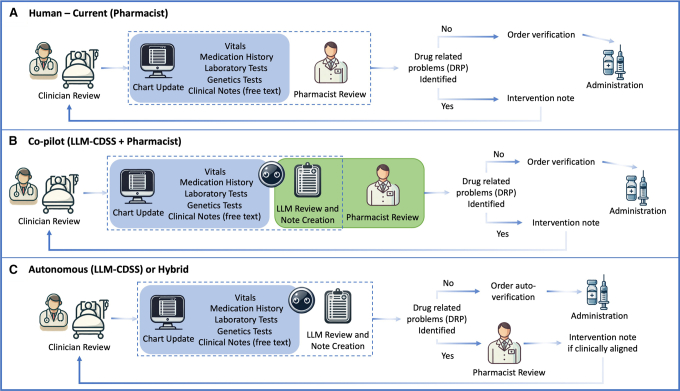
Proposed clinical flow integrating LLM-based CDSS in medication review in acute care setting (A) Describes current state, whereby patient information and medication orders are updated in electronic health record system after clinician review of the patient. In a sequential fashion, the pharmacist reviews the medication order and patient chart and intervenes with prescriber if DRP is identified. (B) LLM-CDSS acts as a co-pilot to summarize patient information, performs a medication review, and creates a note if DRP is identified for pharmacist to preview. (C) Institutions with no access to trained clinical pharmacists or insufficient pharmacy staffing may use LLM-CDSS as an initial screening process for DRPs. Medication orders with DRP will be highlighted for pharmacist review and intervention.

## Discussion

Previous work evaluating the utility of LLMs in medicine focuses heavily on reporting model performance while few prospective studies have evaluated the practical performance of healthcare professionals when LLMs are incorporated as part of a clinical workflow.^25^^,^^26^^,^^27^ In this study, we provide a proof of concept of an application of LLM-based CDSS, designed to improve safety of medication use and prescribing in an acute care practice setting. Our findings are based on 90 different errors and DRPs, embedded within 40 clinical vignettes. Different modes of delivery were tested in a cross-over, open-label study design. We report greater accuracy in DRP identification by 32% when used in a co-pilot mode by pharmacists as compared to pharmacists alone without the tool. Similarly, the accuracy of co-pilot mode in identifying DRPs surpassed that of LLM alone. In co-pilot mode, two-thirds of DRPs with potential for causing serious harm were detected. This study evaluates LLM in assessing prescription appropriateness using patient-specific textual information, which is the most common format encountered by pharmacists during medication reviews. In contrast to prior work that focused on narrow tasks using limited input data,^26^ our study used a combination of patient information and medication details to leverage the general reasoning capabilities of LLMs for evaluating clinical appropriateness. We compared three modes of implementation: LLM alone, human alone, and a collaborative setting where the LLM assisted the user. This approach provides a more comprehensive and realistic understanding of how LLMs can be integrated into clinical practice, addressing limitations of previous studies that lacked direct comparisons or real-world application scenarios.^28^^,^^29^ In addition, previous studies that included a comparator often evaluated LLMs against human experts under the assumption that LLMs will be deployed autonomously in clinical settings.^30^ In contrast, our study examined three configurations: human expert-only, LLM-only, and a co-pilot approach. This provides a more complete and practical understanding of how these models perform when used alongside clinicians in real-world settings.

Our results suggests that, for complex reasoning clinical tasks such as medication chart review, LLMs can potentially augment human performance. However, we observed that, in the co-pilot mode, performance declined in 1 key category of DRP: inappropriate dosage regimen. Variability in medication dosing regimen across different geographical regions and academic institutions has been well described.^31^^,^^32^ In our study, we evaluated the performance of Claude Sonnet 3.5, a commercially available LLM, that was pre-trained on a broad dataset not restricted to carefully curated medication content or the most up-to-date drug dosing guidelines.^33^ These results are consistent with those reported by Yu et al., who found that inaccurate AI predictions negatively impacted the performance of radiologists.^34^ The impact of AI-based decision support tools on user performance has been shown to be heterogeneous, suggesting a need for personalized approaches in the design of clinician-AI interfaces.^35^ The differences between clinician and medical AI decisions are due to a basic mismatch in how each defines the clinical problem. AI systems developed for healthcare applications often lack a clear explanation of how decisions are derived. Although explainable AI tools aim to reveal the reasoning behind an AI’s output, they often use technical explanations that remain confusing or are misleading to users.^35^ This lack of clarity can make it hard for clinicians to trust or effectively use these systems. In contrast, LLMs with conversational interfaces allow clinicians to ask questions and receive context-specific explanations in plain language. We believe that this interactive and adaptive communication can strengthen the partnership between clinicians and AI, making the technology more useful and trustworthy in clinical decision-making.

CDSSs can be broadly classified into two distinct categories: knowledge-based (KB) and AI-driven systems. KB CDSSs rely on a set of rules and associations derived from clinical guidelines or expert opinions. They function by matching patient-specific data with a knowledge base and providing recommendations or alerts.^36^ AI-driven systems, on the other hand, are capable of analyzing large datasets, identify patterns, and make predictions or recommendations based on new data without predefined rules.^7^ Our LLM-based CDSS is an interesting approach to an AI-driven system. Widespread adoption of AI-driven CDSS systems is met with barriers, including lack of transparency, uncertainty relating to the evidence, lack of trust in the system, and disruptions to clinical workflow that add time to routine clinical practice.^37^^,^^38^ To date, ML-based CDSSs are fairly narrow in application, most being domain specific. LLMs grounded with contextual knowledge present various advantages over ML-based models, including the ability to integrate and process vast amounts of varied data types, including unstructured clinical texts;easily update the clinical knowledge corpus without the need for explicit retraining; and offer explanations in natural language that are more comprehensible to human practitioners. In our study, we leveraged upon the generalist capability of LLMs and tested the model in a wide variety of clinical scenarios from different disciplines and included a wide range of medication classes.

However, despite the strengths of LLM-based CDSS, the potential pitfalls of LLM-CDSS tools must be recognized. Although contextually grounded models show promise in reducing hallucinations and improve clinical accuracy of LLMs, our findings suggest that even supplementing an LLM with a knowledge base through the use of RAG did not yield comparatively better results (Figure 2). This might be explained by various reasons. First, our study tested RAG-LLMs on a small number of vignettes and explored a limited range of scenarios. A bigger sample size of scenarios is required to validate this observation. Second, LLMs may demonstrate reluctance to fully accept newly retrieved knowledge, especially when it conflicts with the model’s pretraining data. When conflicting knowledge is integrated, the LLM often produces ambiguous or inconsistent recommendations. Moreover, with regards to LLM-RAG-based CDSSs, RAG systems face challenges due to the lack of a state-of-the-art pipeline or retrieval techniques that have been universally established. This necessitates the creation of highly tailored RAG pipelines for each specific use case, which can be resource intensive and technically demanding. Finally, suboptimal performance of the RAG pipeline in our study can be attributed to the complexity of clinical reasoning required for medication-related tasks, which often exceeds the capabilities of general-purpose retrieval and generation mechanisms. Conflicts between retrieved and pre-trained knowledge and retrieval noise may have compromised the integration of relevant clinical context. Barriers to adopting AI-driven CDSSs, including LLM-based systems, also remain substantial. These systems are met with concerns about their lack of transparency, uncertainty surrounding the evidence base, and disruptions they may cause to clinical workflow, which can add time to routine clinical practice. As such, further refinements and reliable methodologies are required before AI-driven CDSSs can achieve widespread adoption in healthcare.

KB CDSSs are implemented for various purposes and often tailored to the peculiar needs of the healthcare facility. Regarding their efficacy in reinforcing prescribing safety, various studies have shown that KB CDSSs can significantly contribute to the reduction of prescription errors and improve prescribing practices. In a meta-analysis of 68 trials evaluating CDSSs on physician prescribing, positive behavior improved by 4.4% (95% confidence interval [CI] 2.6%–6.2%) with the deployment of KB CDSSs.^36^ AI-based CDSSs have similarly demonstrated positive outcomes on prescribing safety and potential cost savings from DRP avoidance.^37^^,^^38^ These trials underscore the vital role of CDSSs in enhancing prescribing safety, whether through rule-based systems or through advanced AI-driven analytics. However, the successful implementation and effectiveness of these systems depend on several factors, including system design, user interface, integration into clinical workflows, and training of healthcare professionals. LLM-based CDSSs present with unique advantages such as the ability to process and interpret large volumes of unstructured clinical data, adapt to evolving medical knowledge, and provide personalized treatment recommendations.^39^^,^^40^ We foresee that LLM-based CDSSs can be integrated into clinical workflow in co-pilot mode to augment the performance of pharmacists (Figure 4). In addition, we envisage that, in low resource settings with manpower constraints, an autonomous/hybrid mode of AI delivery may plug current gaps in clinical services. In our study, we were unable to perform a direct comparison of our LLM-based CDSS against traditional KB CDSS. This is due to high variability of CDSS design across different healthcare institutions, making standardization of KB CDSSs unfeasible. Another reason was that the inclusion of traditional KB CDSS tools could have introduced confounding factors, such as differences in pharmacists’ familiarity with the tools and the potential influence of automation bias.^41^

**Figure 5:**
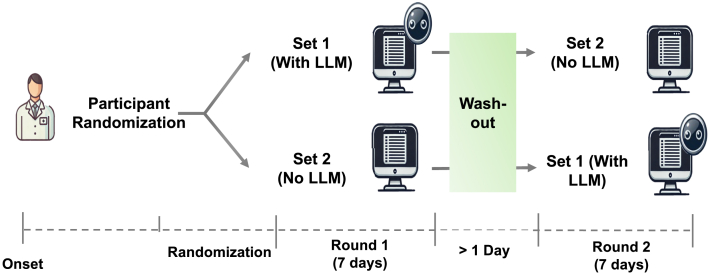
An overview of the cross-over study flow

The limitations of our study are as follows. (1) DRPs identified from clinical scenarios were limited to 5 categories, and this might limit generalizability in clinical cases with other DRP categories. Test scenarios were adapted from errors captured by pharmacists and may represent bias toward scenarios that reach intervention and reporting thresholds. (2) The number of fictional clinical scenarios was limited, notwithstanding that each fictional scenario was designed to be highly complex in order to allow for initial exploration of LLM-based CDSS capabilities. (3) Only 5 LLMs in complex clinical decision-making tasks were evaluated. (4) Bias and fairness of output were not evaluated. Studies have shown that LLM outputs may encode gender and racial biases. (5) In this study, we only tested LLM performance based on one prompt. As different prompt strategies may influence model outputs, future research should focus on designing more precise prompts. (6) Rapid development of new LLM models and versions limits conclusions from study results. (7) Knowledge base data were limited, such as the use of only local institutional monographs and drug use guidelines due to copyright constraints, which may affect generalizability.

Future research should consider several key directions to enhance the accuracy and reliability of LLM-based CDSSs. First, improvements to current tested pipeline are needed, particularly through iterative refinement of information retrieval strategies. This includes chunking and indexing methods, as well as exploration of alternative knowledge sources like real-time online browsing or integration with AI agents. Second, research should focus on optimizing human-computer interaction and clinician-AI collaboration. Although our findings indicate better performance when the model is used collaboratively with clinical pharmacists, critical issues such as model bias, automation bias, and the erosion of trust in AI systems require further evaluation, particularly when deployed at scale in real-world settings. Finally, investigating newly released models with advanced reasoning capabilities, such as OpenAI’s o3-mini and DeepSeek’s R1, offers promising potential. These models support chain-of-thought reasoning, which can enhance transparency by revealing the steps behind an output, thereby addressing concerns around the “black box” nature of many AI systems and improving user trust.

The integration of LLM-based CDSSs presents as a potential tool in improving prescription safety. Our study reveals that, when used in tandem with pharmacists, identification of DRPs is enhanced, surpassing the accuracy of humans alone. The combined pharmacist and LLM model (co-pilot mode) demonstrates superior performance in detecting severe DRPs and offers a promising hybrid solution for improving patient safety in medication management. This co-pilot model could represent the next step in CDSS development, merging human expertise with the analytical prowess of AI to improve healthcare outcomes.

### Limitations of the study

This study’s limitations constrain its immediate clinical applicability. It addressed only five DRP categories using a small set of complex, fictional scenarios that may not reflect the diversity of real-world cases. Test cases were drawn from known pharmacist-reported errors, introducing potential bias. The evaluation of five LLMs with a single prompt, without assessing output bias or fairness, further limits generalizability. Additionally, rapid LLM evolution and reliance on local drug references restrict applicability across broader clinical settings. These factors underscore the need for further validation before integrating such tools into routine clinical practice.

## Resource availability

### Lead contact

Further information and requests for resources should be directed to and will be fulfilled by the lead contact, Dr. Daniel Shu Wei Ting (daniel.ting.s.w@singhealth.com.sg).

### Materials availability

This study did not generate new unique reagents.

### Data and code availability

• Individual participant data will be made available on reasonable request, such as for research collaboration, directed to the corresponding author (also the lead contact), Dr. Daniel Shu Wei Ting.
• Sample dataset and codes are openly available at https://github.com/Liyuan1Y/CDSS-LLM-Cookbook/tree/main.
• Any additional information required to reanalyze the data reported in this work paper is available from the lead contact upon request.

## Acknowledgments

This work was supported by the 10.13039/501100001349National Medical Research Council, Singapore (grants MOH-001689-00, MOH-000655-00, and MOH-001014-00); 10.13039/100016017Duke-NUS Medical School (grants Duke-NUS/RSF/2021/0018, 05/FY2020/EX/15-A58, and 05/FY2022/EX/66-A128); and 10.13039/501100001348Agency for Science, Technology and Research, Singapore (grants A20H4g2141 and H20C6a0032).

## Author contributions

J.C.L.O., L.J., K.E., and D.S.W.T. developed the initial concept, design of the study, and the initial manuscript draft. R.J.Z., C.M.Y.K., K.Z.H.L., and X.C. refined the study design and analyzed results. G.Y.S.L., D.Y.Z.L., G.G.R.S., Y.H.K., J.Y.M.T., J.K.C., A.T., K.J.G., C.P.L., T.M.N., and N.L. refined and vetted the manuscript.

## Declaration of interests

The authors declare no competing interests.

## STAR★Methods

### Key resources table

**Table:** 

REAGENT or RESOURCE	SOURCE	IDENTIFIER
**Software and algorithms**		

Gemini Flash, Gemini 1.5 Pro, GPT-4 Turbo, GPT-4 omni, Claude 3.5 Sonnet	Google, OpenAI, Anthropic	Sample dataset and code can be accessed at: https://github.com/Liyuan1Y/CDSS-LLM-Cookbook/tree/main. Any additional information required to reanalyse the data reported in this work paper is available from the lead contact A/Prof Daniel Ting upon request at daniel.ting.s.w@singhealth.com.sg

### Experimental model and study participant details

This was a prospective, cross-over, open-label study. The institutional ethics review board exempted this study from review as no identifiable patient data was used. An overview of the evaluation workflow is shown in Figure 5. Participants were registered pharmacists, practicing in 2 different tertiary acute care institutions. Participants were randomized using a random number generator into one of 2 arms. In the first arm, participants reviewed a set of patient case vignettes without LLM-CDS assistance in round 1. Participants waited at least one day before they are asked to review another set of patient case vignettes with appended LLM-CDS output in round 2. In the second arm, participants reviewed a set of case vignettes with LLM-CDS assistance in round 1, followed a set of vignettes without LLM-CDS assistance after the washout period. During each round, participants were asked to complete the reviews of 7–8 case within a time frame of 1.5 h. Rounds consisted of a fresh set of 7–8 vignettes (not used in the previous round). We provide details of response generation in subsection on “Generation of LLM and Pharmacist Responses”.

#### Development of prescribing error scenarios

A total of 91 different simulated prescribing error scenarios based on 40 case vignettes modeled after complex clinical cases were curated. Prescribing error scenarios were adapted from our institution’s pharmacy intervention and error reporting databases to maintain realism. The vignettes covered clinical scenarios from 16 different medical or surgical subspecialties (Cardiology, Colorectal Surgery, Endocrinology, Emergency Medicine, Family Medicine, Gastroenterology, General Surgery, General Medicine, Haematology, Infectious Disease, Neurology, Oncology, Ophthalmology, Respiratory Medicine, Urology, Vascular Surgery) with some cases involving more than one discipline. Each case vignette consisted of a patient clinical note and medication prescription. Prescribing errors in the clinical scenarios were designed to be reflective of drug-related problems (DRP) encountered in an acute care tertiary medical institution in Singapore. We present a sample of one case vignette in Figure S1. A detailed description of all case vignettes is found in Data S1. The Anatomical Therapeutic Chemical (ATC) category of medications prescribed in each clinical scenario is also presented in Table S2.

#### Development of reference standard

The reference standard was developed through manual grading of DRP categories and severity by a multi-disciplinary expert panel. The expert panel consisted of pharmacotherapy board certified pharmacists and physicians with >10 years of clinical practice experience in tertiary hospitals. Every clinical scenario was graded by at least 1 pharmacist and 1 physician member of the panel. Any disagreements were resolved by a 3^rd^ independent member. DRPs from each clinical scenario were categorized using the Pharmaceutical Care Network Europe (PCNE) classification V9.1 and ASHP statement of pharmaceutical care as a guide.^42^^,^^43^ Types of errors in the clinical scenarios included adverse drug reaction, allergy, drug-drug interactions, duplication of therapy, inappropriate choice of therapy, inappropriate dosage regimen, no indication and omission of drug therapy. The potential severity of these errors were graded according to the Harm Associated with Medication Error Classification (HAMEC) classification tool.^44^ In addition, we calculated the MCRI (medication regimen complexity index) for each case. The MCRI is a 65-item instrument validated across different study populations to quantify medication complexity at patient level and identify patients who require medication therapy management interventions. This instrument demonstrated moderate to good concordance between patient-level MRCI scores and experts in ranking of medication regimen complexity.^45^

#### Development and validation of LLM tool

We first evaluated the performance of 5 different state-of-the-art LLMs (Gemini Flash, Gemini 1.5 Pro, GPT-4 Turbo, GPT-4 omni, Claude 3.5 Sonnet) with and without provision of contextual knowledge. We then developed and validated a retrieval augmented generation (RAG) based-LLM model through optimizing the transformation of specialized documents into embedding vectors. This process utilizes advanced pre-processing and embedding models, with a focus on similarity-based retrieval between query vectors and embedded vectors of targeted documents, including drug monographs and hospital drug-use protocols.

Our RAG framework integrated the Llamaindex, relying on auto-merging retrieval to provide contextualized search. We optimized pharmacological knowledge corpus with manual indexing of drug names improved specificity during retrieval. Total number of retrieved passages were set as 50, expanded with increased context size in latest LLMs, for optimal breadth and depth of information retrieval.

The prompt was designed as a series of tasks, simulating human clinical thinking process. Each task required retrieval of knowledge for each medication on the prescription (e.g., Is this medication indicated for the patient?). The response from each task is collated into a final LLM model for clinical synthesis and recommendations. A diagrammatic view of the sequence is shown in Figure S2. All responses were generated and analyzed in triplicates to account for reproducibility.

#### Knowledge source

Institutional medication use and dosing guidelines, medication monographs were used as sources of information. Each medication monograph was split into 4 separate sections according to the following categories of information: (1) Adverse drug reactions, cautions and contraindications, (2) ATC category and mechanism of action, (3) Drug-drug interactions, (4) Drug dosing and adjustments.

#### LLM prompt

We designed a prompt strategy informed by prior research and expert recommendations. Studies have demonstrated that prompting methods such as zero shot, few shot, and chain of thought can significantly influence LLM performance in medical applications, with no single approach universally optimal across all models and tasks. Based on this, we followed best practice guidelines and adopted a chain-of-thought prompting format. This approach was chosen to reflect the structured, step by step reasoning processes commonly used by clinicians and pharmacists, thereby enabling a more clinically relevant assessment of the LLM’s performance in evaluating medication appropriateness.^46^ The final adapted prompt used is presented in Figure S3. To standardize LLM outputs, we prompted the models to present its final recommendations in a SOAP (Subjective, Objective, Assessment and Plan) format.

#### Generation of LLM and Pharmacist Responses

Native LLM and RAG-LLM were presented with all case scenarios to generate a response. For reproducibility, we generated all LLM and RAG-LLM outputs in triplicates, resulting in a total of 1,200 responses for assessment. Respective performance was used to select the best model to be used as comparator against human only and for adoption in co-pilot mode.

We randomly assigned the scenarios to participants to independently identify any DRPs and generate a standard clinical note in standardized healthcare SBAR (situation, background, assessment, recommendation) format for each scenario. Each pharmacist had access to all institutional protocols and guidelines.

#### Assessment of accuracy of responses

We evaluated the accuracy of all DRP responses using the human expert panel as the criterion standard (Table S1). To determine whether the LLMs accurately identified these issues, we employed a structured methodology:

(1) Evaluation was grounded upon internationally accepted definition of a drug-related problem, specifically the one provided by the Pharmaceutical Care Network Europe (PCNE), version 9.1. According to this definition, “A Drug-Related Problem is an event or circumstance involving drug therapy that actually or potentially interferes with desired health outcomes.”
(2) LLM output should identify the medication order(s) that posed potential risk(s) to the patient.
(3) Third, LLM should propose an appropriate action or intervention. Recommended actions were evaluated using a rubric of acceptable actions, adapted from the PCNE classification system for DRPs (Table S2).
(4) Finally, an overall assessment (global assessment) of the LLM output for the presence of any clinically significant risk to the patient.

For a response to be graded as accurate, the DRP response should fulfill criteria (1) – (3), and assessed to present no clinically significant risk to patient as described in (4).

### Method details

This prospective, crossover, open-label study assessed the effectiveness of a large language model (LLM)-based clinical decision support system (CDSS) in identifying prescribing errors. Pharmacists from two tertiary hospitals reviewed 91 prescribing error scenarios across 40 complex clinical vignettes, drawn from 16 medical specialties. Each participant completed reviews in two randomized arms: with and without LLM-CDSS assistance, separated by a washout period. A multi-disciplinary expert panel annotated each case using the Pharmaceutical Care Network Europe (PCNE) v9.1 and HAMEC frameworks to classify drug-related problems (DRPs) and assess potential harm severity.

Five state-of-the-art LLMs (Gemini Flash, Gemini 1.5 Pro, GPT-4 Turbo, GPT-4 omni, Claude 3.5 Sonnet) were tested, both in their native forms and within a retrieval-augmented generation (RAG) framework. The RAG system incorporated LlamaIndex with auto-merging retrieval and indexed pharmacological knowledge (e.g., drug monographs, dosing protocols). Prompts followed a chain-of-thought structure and were standardized to a SOAP format. Responses were generated in triplicate for each case to assess reproducibility. Pharmacist responses were produced in SBAR format. Claude 3.5 Sonnet, the top-performing model, was selected for the co-pilot arm.

Accuracy was determined by comparing LLM and pharmacist outputs to expert consensus. A response was marked correct if it identified the relevant medication and DRP, recommended an appropriate intervention, and posed no risk of harm. Quantitative metrics included accuracy, precision, recall, and F1 score, calculated using R version 4.3.0. Statistical analysis included one-way ANOVA and Pearson correlation with the Medication Regimen Complexity Index (MCRI). To evaluate consistency across LLM responses, BERT scores, BLEU scores, and cosine similarity were computed, indicating high reproducibility of model outputs.

### Quantification and statistical analysis

Concordance between performance of co-pilot and autonomous modes, using human expert input as the benchmark, was measured in terms of accuracy, precision, recall, and F1 score. Accuracy is expressed as a percentage of correctly identified DRP against expert. Precision, which denotes the fraction of DRPs correctly identified amongst all suggested DRPs, was defined as precision = true positives/(true positives + false positives). Recall, or the fraction of all DRPs in the criterion standard correctly identified by LLMs, was defined as recall = true positives/(true positives + false negatives). The F1 score is the harmonic mean of precision and recall, and thus penalizes unbalanced precision and recall scores (i.e., is higher when both precision and recall have similar values): F1 score = (2 × precision × recall)/(precision + recall). The higher any of the 3 scores, the better the response, with 1 being the maximum value for each score. One-way ANOVA was applied to compare differences in accuracy between different modes with a one-tailed α = 0.05. Pearson’s correlation analysis was performed to examine correlation between MCRI and accuracy. Data analysis, calculation of precision, recall and F1 scores were done in R version 4.3.0 (R Project for Statistical Computing).

BERT scores, Cosine Similarity and BLEU scores were generated to assess semantic similarity across repeated LLM responses. These metrics were chosen to assess both semantic consistency and lexical overlap between responses. All LLM responses were generated via consecutive API calls in a single session. To quantify overall reproducibility of LLM responses, the sum and average of each metric across the three comparisons were calculated. This provides a structured assessment of LLM consistency across multiple response iterations.

## Supplemental information

Document S1. Figures S1–S3, Tables S1–S3, and Data S1

Document S2. Article plus supplemental information
