# Supplementary material for: Large language model as clinical decision support system augments medication safety in 16 clinical specialties
Source: Cell Rep Med. 2025 Sep 24;6(10):102323. doi: 10.1016/j.xcrm.2025.102323 (PMC12629785; doi:10.1016/j.xcrm.2025.102323)
Supplement: Document S1. Figures S1–S3, Tables S1–S3, and Data S1 [file mmc1.pdf]

Cell Reports Medicine, Volume *6*

Supplemental information

Large language model as clinical decision support system augments medication safety in 16 clinical specialties

Jasmine Chiat Ling Ong, Liyuan Jin, Kabilan Elangovan, Gilbert Yong San Lim, Daniel Yan Zheng Lim, Gerald Gui Ren Sng, Yu He Ke, Joshua Yi Min Tung, Ryan Jian Zhong, Christopher Ming Yao Koh, Keane Zhi Hao Lee, Xiang Chen, Jack Kian Ch'ng, Aung Than, Ken Junyang Goh, Chuan Poh Lim, Tat Ming Ng, Nan Liu, and Daniel Shu Wei Ting

Table of Supplementary Data

Title	Legend
Figure S1: Sample of Case Vignette.	Abbreviations (CVM: cardiovascular Medicine, BP: blood pressure, RR: respiratory rate, Ht: height, BMI: body mass index, T: temperature, Hb: hemoglobin, TW: total white count, Plt: platelet count, SCr: serum creatinine, INR: international normalized ratio, PMHx: past medical history, DM: diabetes mellitus, HTN: hypertension, HLD: hyperlipidemia, CKD: chronic kidney disease, BKA: below knee amputation , OM: osteomyelitis, I&D: incision and drainage, DVT: deep vein thrombosis, PE: pulmonary embolism, VAS: vascular, HOPC: history of presenting complain, CABG: coronary artery bypass grafting, NSAID (non-steroidal anti-inflammatory drug), LV: left ventricle, RV: right ventricle, EF: ejection fraction, MI: myocardial infarction, O/E: on examination, NSR: normal sinus rhythm, JVP: jugular venous pressure, SNT: soft non tender, BS: bowel sound, LAD: left anterior descending artery, AV: atrioventricular, LCx: left circumflex artery, ICA: intermediate care area, DAPT: dual anti-platelet therapy)
Figure S2: Overview of RAG-LLM architecture with auto-merging retrieval	NA
Figure S3: Final adapted prompt	NA
Table S1: Rubric to evaluate acceptable actions produced by LLM based on DRP category	NA
Table S2: DRPs and Risk / Potential for Harm Categories	NA
Table S3: Results from reasoning model (OpenAI's o4-mini)	SD: Standard Deviation
Data S1: Summary of Case Vignettes	NA

Figure S1: Sample of Case Vignette.

CVM Inpatient Daily Ward Round
General Information:
Admission Date 11-Sep-2024 14:24:49 Post Admission Day 2.

Clinical Notes:
Latest Vital Signs: 12-Sep-2024 07:00:00
12/09/2024 07:00:00
Pain Score: 0
BP (NIBP) (mmHg): 103/83 (101-144/68-118), **HR (beats/min):** 65 (55-99)
RR (breaths/min): 12 (12-21), **SPO2 (%):** 100 (95-100), **O2 Therapy (L/min):** NP 2 (2-3)
Hypocount (from 11/09/2024 06:00:00 to 12/09/2024 07:18:37):
15.8(H) <- 20.4(H) <- 27.5(HH) <- 21.0(H) <- 5.9(N)
Ht: 182 cm (11-Sep-2024 15:18:00), **Wt:** 99.1 kg (11-Sep-2024 16:57:00)
BMI: 29.9, **BSA:** 2.24 m2
12/09/2024 04:00:00
T (deg.C): 36.6, **Tmax (deg.C):** 36.6 (12/09/2024 04:00:00)

Latest I/O from 11/09/2024 06:01 to 12/09/2024 06:00
Intake: 2100 Output: 3750 Net: -1650
Intake: 2100
- Diet Fluid Volume: 100
- IV: 2000
Output: 3750
- Urine Output: 3750

Lab Values:
12/09/2024 06:15
Hb: **11.3 [12.0 – 16.0 G/DL]**
TW: 8.64 [4.0 – 10.0 x 10(9)/L]
Plt: 250 [140 – 440 x 10(9)/L]
SCr: 100 [37 – 75 UMOL/L]
INR: 1.2

Surgical operations:
right LL deep vein thrombolysis and creation of SFA-LSV arteriovenous fistual on 15-May-2024.

Clinical Notes:
61 / M / Malay
Allergic to mefenamic acid and salicylate, claims rashes and facial swelling
Lives with wife and children
ADL independent, Community ambulant

PMHx:
1. Poorly controlled DM
2. HTN, HLD
3. CKD Stage 2
4. Necrotising fasciitis of R LL
- s/p R BKA 12/2/20
5. Left foot OM
- s/p 2nd and 3rd ray amputation Dec 2019
6. Large right medial thigh abscess
- s/p I&D 6/3/24
7. Recurrent DVT and PE (on long-term warfarin) - f/u Haem and VAS
- 2018: L LL DVT
- 2020: Extensive left arm DVT s/p R LL thrombolysis and IVC filter insertion
- s/p R LL venous thrombectomy with stenting 28/4/24
- Possible right interlobar pulmonary embolism (April 2024)

HOPC:
Chest pain since Friday
Started at rest
Central pressing with dyspnoea and diaphoresis
No radiation
Stuttering since then
Worse with exertion
Worse since 5am today hence presented
Still has ongoing mild discomfort
No fever or intercurrent illness
No bleeding history
Normally takes warfarin at night - has not taken for today
All his brothers had CABG at his age
Single NSAID (mefenamic acid) allergy - rashes
Progress in emergency department:
Bedside echo: LV impaired systolic function (also does look dilated), RV normal size and function, EF < 45%
ECG: evolved anterior MI (anterior Q waves new since May 2024)
Cath lab activated

O/E:
Vitals stable, afebrile
SpO2 95-100% on 2LNP
Poor H/C control
Telemetry ON: NSR
JVP not elevated
H S1 S2 NIL murmurs
L Clear
Abdomen SNT, BS+

Issues and progress:
1) Evolved anterior MI with ongoing chest discomfort
- Cath activated
- Findings:
- Co-dominant coronary system
- Severe proximal to mid LAD stenosis, involving the bifurcation with the dominant diagonal branch
- Diffusely diseased AV groove branch (continuation of the distal LCx)
- Anomalous origin of the RCA (high anterior). Diffuse moderate proximal to mid RCA narrowing.
- Successful PCI to the LAD and implantation of overlapping drug eluting stent (Onyx) from mid to proximal LAD. Ostium of the diagonal branch preserved with modified jailed balloon technique.
- Post cath stable - to ICA
2) b/g of recurrent VTE on Warfain
Hold off warfarin for now
Bridge with clexane
Trend Hb whilst on DAPT + anticoagulation
- Hb on 12/9 11.3 (stable)

Plan:
To GW with telemetry
Vitals as per ward protocol
CBG TDS + 10PM with SCSI cover
Low salt/low fat/DM diet
Heart failure for medical therapy in the interim
Aspirin for 1/12
Clopidogrel for at least 12 months

Allergies:
Mefenamic Acid. Facial Swelling.
Salicylate. Facial Swelling

**Medications Prescribed**	**Status**
Sodium Chloride 0.9% InFUSion, IV Intermittent 2,000 mL, Once, Infuse Over 16 hour, 125 mL/hr	Active
Enoxaparin Sodium Injection, Sub-Cutaneous 60 mg, BD	Active
ACTRAPID [Insulin Soluble] Injection, Sub-Cutaneous 4 unit, Once	Active
LANTUS [Insulin Glargine] Solostar, Sub-Cutaneous 24 unit, OM	Active
NovoRAPID [Insulin Aspart] Flexpen, Sub-Cutaneous 8 unit, TDS (Pre-meal)	Active
Aspirin Tablet, PO 100 mg, OM	Active
Clopidogrel Tablet, PO 75 mg, OM	Active
OMEprazole Capsule, PO 20 mg, OM	Active
Glyceryl Trinitrate Tablet, Sub-Lingual 0.5 mg, Use as directed PRN Chest Pain	Active
Linagliptin Tablet, PO 5 mg, OM	Active
Bisoprolol Fumarate Tablet, PO 2.5 mg, OM	Active
Perindopril Erbumine [Tert-butylamine] Tablet, PO 2 mg, OM	Active
Neurobion Tablet [Vit B1 100mg, B6 200mg, B12 200mcg], PO 1 tablet, OM	Active

Abbreviations (CVM: cardiovascular Medicine, BP: blood pressure, RR: respiratory rate, Ht: height, BMI: body mass index, T: temperature, Hb: hemoglobin, TW: total white count, Plt: platelet count, SCr: serum creatinine, INR: international normalized ratio, PMHx: past medical history, DM: diabetes mellitus, HTN: hypertension, HLD: hyperlipidemia, CKD: chronic kidney disease, BKA: below knee amputation , OM: osteomyelitis, I&D: incision and drainage, DVT: deep vein thrombosis, PE: pulmonary embolism, VAS: vascular, HOPC: history of presenting complain, CABG: coronary artery bypass grafting, NSAID (non-steroidal anti-inflammatory drug), LV: left ventricle, RV: right ventricle, EF: ejection fraction, MI: myocardial infarction, O/E: on examination, NSR: normal sinus rhythm, JVP: jugular venous pressure, SNT: soft non tender, BS: bowel sound, LAD: left anterior descending artery, AV: atrioventricular, LCx: left circumflex artery, ICA: intermediate care area, DAPT: dual anti-platelet therapy)

Figure S2: Overview of RAG-LLM architecture with auto-merging retrieval

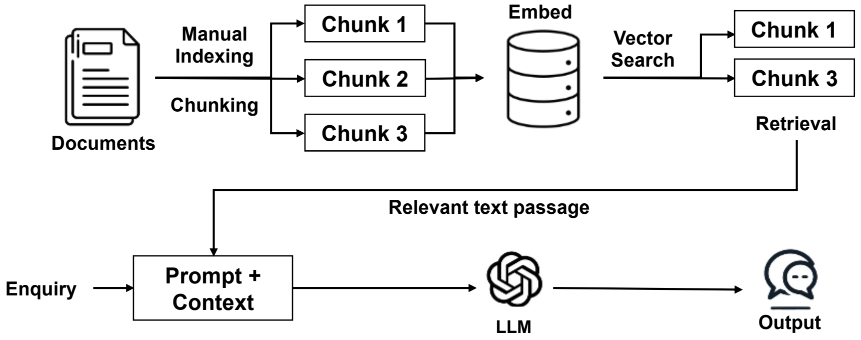

Figure S3: Final adapted prompt

For the Junior Pharmacist

Assume the role of a clinical pharmacist. You are tasked to perform a medication chart review for a patient admitted to the department of <cardiology>. I will provide you with the patient's medication list, clinical note, and drug monographs as reference. Identify drug related problems specific to the patient's profile using this guide:

- Medication Indications: Confirm that each medication has a clear indication and that current health conditions are being addressed with appropriate pharmacotherapy. [Drug monograph reference sections: "Pharmacologic Category", "Use: Labeled Indications", "Use: Off-Label: Adult", "Mechanism of Action"]
- Dosing Verification: Check that the dosages of medications are within the recommended ranges and adjust if necessary, considering factors such as age, kidney function, and liver function [Drug monograph reference sections: "Dosing: Adult", "Dosing: Older Adult", "Dosing: Altered Kidney Function: Adult", "Dosing: Hepatic Impairment: Adult"]
- Drug-Drug Interactions: Investigate potential interactions between current medications that could increase the risk of adverse effects or reduce therapeutic efficacy and warrants a change in therapy or monitoring tests. [Drug monograph reference sections: "Metabolism/Transport Effects", "Drug Interactions"]
- Potential adverse drug reaction, contraindications and cautions, medication allergy [Drug monograph reference sections: "Special alerts", "ALERT: U.S. Boxed Warning", "Warnings/Precautions", "Contraindications", "Adverse Reactions", "Adverse Reactions (Significant): Considerations"]
- Medication Omissions: Look for any conditions that are not being treated which should be, according to the patient's history and current clinical guidelines.
- Any duplication in medication class or therapy
- Patient-Specific Factors: Take into account patient-specific factors such as age, allergies, and preferences that may influence medication selection and management.

Create a pharmacist recommendation note to address any identified drug related problem(s) in the following format: "situation, background, assessment, recommendation". Your plan should be clear and justified with specific recommendations for any changes to the medication regimen, including discontinuations, dose adjustments, or additions.

Table S1: Rubric to evaluate acceptable actions produced by LLM based on DRP category

Category	Description	Acceptable Action
Drug Selection	The cause of the DRP can be related to the selection of the drug, e.g. No indication for drug, inappropriate selection of drug, untreated indication	Discontinue medication; Substitute medication; Initiate medication for untreated indication
Drug Form	The cause of the DRP is related to the selection of the drug formulation	Substitute with different formulation of medication; Substitute with different medication of same formulation; Suggest different route of administration
Dose Selection	The cause of the DRP can be related to the selection of the dosage schedule or dosing regimen	Amend dosing regimen: dose or frequency
Treatment duration	The cause of the DRP is related to the duration of treatment	Amend medication duration; Discontinue medication; Resume medication (unintended discontinuation)

Table S2: DRPs and Risk / Potential for Harm Categories

Case No	DRP(s) Category and Description	Severity / Potential for Harm
1	(1) Drug allergy: Background of NSAIDS allergy (rash) but prescribed with aspirin without any challenge or test dose	Moderate
	(2) Inappropriate dosage regimen: Enoxaparin dosed at 60mg BD in obese patient of 90kg, no bleeding.	Serious
	(3) Omission of therapy: Omission of statin therapy in patient presenting with myocardial infarction	Moderate
	(4) Adverse drug reaction: Borderline blood pressure but prescribed with perindopril and bisoprolol	Moderate
2	(1) Drug Drug Interaction: Significant Interaction between atorvastatin and clarithromycin	Moderate
	(2) Inappropriate dosage regimen: Colchicine dosed in MG instead of MCG	Serious
	(3) Adverse drug reaction: Bradycardia but prescribed with both bisoprolol and ticagrelor	Moderate
3	(1) Wrong indication: Wrong drug of clarithromycin instead of clindamycin for cellulitis in patient with penicillin allergy	Serious
	(2) Inappropriate dosage regimen: Wrong dose of enoxaparin (dosed 1mg/kg BD) in patient with renal failure	Moderate
	(3) Drug drug interaction: Significant drug interaction (contraindicated) between sildenafil and isosorbide mononitrate	Serious
4	Control Case	NA
5	(1) Adverse drug reaction: Patient presented with hypokalemia, on intensive furosemide therapy without electrolyte replacement	Serious
	(2) Adverse drug reaction: Patient presenting with acute pulmonary edema but continued on beta-blocker therapy	Moderate
	(3) No indication for medication: Patient on triple antithrombotic therapy with aspirin / clopidogrel / enoxaparin post myocardial infarction, also continued on dipyridamole (chronic medication for previous history of CVA)	Serious
6	(1) Duplication of therapy: Duplication between Simvastatin and Atorvastatin. Both belong to the same category of HMG-CoA reductase inhibitor	Minor
	(2) Inappropriate dosing frequency for atorvastatin. It is usually given once daily, but does not exceed max daily dose	Minor
	(3) Adverse Drug Reaction: Patient has allergy to amoxicillin (rash) but was prescribed with Co-amoxiclav which contains amoxicillin	Moderate
7	(4) Adverse Drug Reaction: Acute kidney injury with hyperkalemia but continued on enalapril	Moderate
	(5) Adverse drug reaction: Acute kidney injury but prescribed with NSAID	Moderate
	(6) Adverse drug reaction: Presented with hypoglycemia but continued on glipizide	Serious
8	(1) Inappropriate dosage regimen: Wrong weight-based dose of calcitonin prescribed for patient with hypercalcemia of malignancy	Serious
	(2) Adverse drug reaction: Colecalciferol not held off despite hypercalcemia	Moderate
	(3) No indication: Wrong drug of cinnarizine prescribed instead of cinacalcet for patient with b/g secondary hyperparathyroidism	Minor
9	(1) Duplication of therapy: Patient with poorly controlled diabetes on both ultra-short-acting insulin Aspart and short-acting insulin Actrapid.	Moderate
	(2) Omission of therapy: Omisson of basal insulin such as insulatard in patient with poorly controlled diabetes	Moderate
	(3) Omission of therapy: Antibiotics not ordered for patient with suspected sepsis	Serious
10	(1) Adverse drug reaction: Laboratory results demonstrating non-anion gap metabolic acidosis with respiratory compensation, continued on acetazolamide	Moderate
	(2) Adverse drug reaction: Hydroxyzine (first generation antihistamine with significant anticholinergic effects) contraindicated in acute-angled glaucoma	Moderate
	(3) Adverse drug reaction: Patient with frequent urinary tract infection but continued on dapaglifozin, an SGLT-2 inhibitor	Moderate
11	(1) Duplication of therapy: Patient prescribed with PO prednisolone and IV Methylprednisolone (high-intensity) concurrently	Minor
	(2) Inappropriate dosage regimen: Levothyroxine prescribed in MG instead of MCG	Serious
12	(1) Drug drug interaction: Significant interaction between tramadol and linezolid leading to increased risk for serotonin syndrome	Moderate
	(2) Duplication of therapy: Patient prescribed with IV Tienam and PO ciprofloxacin for gram-negative coverage of urinary tract infection	Minor

	(3) Inappropriate dosage regimen: Instructions for prednisolone eye drops was to administer to left eye, when the affected eye was the right	Serious
13	(1) Drug drug interaction: Significant (contraindicated) interaction between azathioprine and allopurinol leading to increased risk for neutropenia	Serious
	(2) Omission of therapy: Patient initiated on allopurinol but not on anti-inflammation therapy to avoid exacerbation of gout flare (e.g. colchicine or steroids)	Moderate
14	(1) Omission of therapy: Noted complains of no bowel movement for n-days but no laxatives ordered for patient with a significant history of liver cirrhosis	Moderate
	(2) Drug drug interaction: Significant interaction between Epclusa and omeprazole that will reduce absorption and efficacy of anti-viral agent	Moderate
	(3) Inappropriate dosage regimen: Dosage of paracetamol not adjusted in the presence of liver cirrhosis	Moderate
15	Control Case	NA
16	(1) Drug drug interaction: Significant interaction between calcium supplement and tetracycline. Calcium reduces absorption of tetracycline when taken concurrently	Moderate
	(2) Inappropriate dosage regimen: Dose of metronidazole not adjusted in the presence of significant liver impairment	Moderate
17	(1) Inappropriate dosage regiment: The infusion rate of vancomycin has exceeded the maximum recommended rate, leading to increased risk for red man's syndrome	Serious
	(2) No indication: G-CSF (filgrastim) continued despite recovery of absolute neutrophil counts	Moderate
18	(1) Drug drug interaction: Significant interaction between ciclosporin and atorvastatin, with max dose of atorvastatin limited to not more than 20mg per day	Moderate
	(2) Inappropriate dosage regimen: Inappropriate dose of co-trimoxazole, prescribed in trimethoprim component instead of co-trimoxazole for PCP prophylaxis	Moderate
19	(1) Inappropriate dosage regimen: Dose of tramadol not adjusted in the presence of significant renal failure	Moderate
	(1) Inappropriate dosage regimen: Dose of gabapentin not adjusted in the presence of significant renal failure	Moderate
	(2) Omission of therapy: Aspirin omitted in patient with significant cardiac history and low risk of bleeding	Minor
	(3) Drug drug interaction: significant interaction between sulphonylurea and ciprofloxacin leading to increased risk for hypoglycemia	Minor
20	(1) Duplication of therapy: Celecoxib and etoricoxib overlapping mechanism of action	Minor
	(2) Duplication of therapy: PO and IV omeprazole both ordered	No harm
	(3) Adverse drug reaction: Initiation of hydralazine in patient with a background of lupus disease	Serious
21	(1) Adverse Drug Reaction: Hyperkalemia but continued on potassium chloride infusion	Serious
	(2) Inappropriate dosage regimen: Exceeded maximum recommended dose of omeprazole for the indication of ulcer prevention	Minor
	(3) Drug drug interaction: Significant interaction between prochlorperazine and metoclopramide	Serious
	(4) Omission of therapy: Untreated hyperlipidemia	Minor
	(5) Omission of therapy: Untreated hyperkalemia	Serious
22	(1) Inappropriate dosage regimen: Rapid intravenous infusion of potassium chloride 10mmol over 1 minute	Moderate
	(2) Omission of therapy: Omission of steroid (e.g. prednisolone) when on abiraterone treatment for castrate resistant bladder cancer	Moderate
	(3) Adverse drug reaction: Diclofenac use in patient with recent myocardial infarction presenting with heightened risk for cardiovascular events	Serious
23	(1) Duplication of therapy: Prescription of 2 alpha-blockers (Tamsulosin and Alfuzosin) in a patient with history of benign prostate hyperplasia	Minor
	(2) Wrong choice of therapy: Co-amoxiclav use in patient with wound culture growing *E.Coli* reported to be resistant to co-amoxiclav	Moderate
24	(1) Adverse drug reaction: Trimetazidine MR contraindicated in renal failure	Moderate
	(2) Adverse drug reaction: Digoxin continued despite renal failure and high digoxin levels	Serious
25	(1) Drug drug interaction: Tramadol contraindicated in SSRI overdose	Serious
	(2) Allergic reaction: Ketoprofen prescribed in patient with ibuprofen allergy	Serious

26	(1) Adverse drug reaction: Empaglifozin not held off in patient with infection and possible bleeding	Moderate
	(2) Duplication of therapy: Linaglpitin and sitagliptin both DPP-IV Inhibitors prescribed together	Moderate
27	(1) Duplication of Therapy: Patient is on neurobion and pyridoxine	Minor
	(2) Inappropriate dosage regimen: Antihypertensives regimen to be streamlined	Moderate
28	(1) Adverse drug reaction: Statin therapy in patient with elevated liver function test (ALT/AST > 3x ULN) from cholangiits and shock	Serious
	(2) Omission of therapy: No sliding scale insulin in hospitalized patient with elevated glucose level	Moderate
29	(1) Adverse drug reaction: IV Iron therapy continued in patient with active infection	Moderate
	(2) Adverse drug reaction: Azithromycin prescribed in patient with history of long QT syndrome	Serious
30	(1) Inappropriate drug selection: Novorapid (short-acting insulin) ordered as novomix, prescribed three times a day	Serious
31	(1) Monitoring and tests: IgA antibody titres not checked before Intragam infusion	Serious
	(2) Drug Interaction: Tramadol and dextromethorphan and risk for serotonin syndrome	Moderate
	(3) No indication for drug: sodium bicarbonate in high bicarbonate levels	Moderate
32	(1) Adverse drug reaction: Nifedipine immediate release intead of long acting formulation prescribed in 78 year old patient with hypertensive emergency.	Moderate
	(2) Inappropriate dosage regimen: Sitagliptin dose not adjusted in CKD	Moderate
33	(1) Inappropriate dosage regimen: Antibiotic doses (Piperacillin-Tazobactam and Vancomycin) not adjusted after termination of contious renal replacement therapy	Moderate
	(2) Adverse drug reaction: Magnesium sulfate injection given bolus for hypomagnesemia	Serious
34	(1) Drug Drug Interactions: Patient on warfarin prescribed with metronidazole without adjustments to warfarin dosage or closer INR monitoring	Serious
35	(1) Drug Drug interaction: Patient on posaconazole suspension for chronic suppression started on high dose IV omeprazole	Moderate
	(2) Inappropriate dosage regimen: Dose of potassium chloride too low for potassium replacement in adult patient with K level of 3.1 mmol/L	Moderate
36	(1) Drug Allergy: Patient with DRESS from esomeprazole, prescribed with omeprazole	Serious
	(2) Adverse Drug Reaction: Continued on aspirin and clopidogrel during immediate post EVT phase	Serious
37	(1) No indication for drug: Zirconium Cyclosilicate prescribed for pseudohyperkalemia (hemolysed blood sample)	Moderate
	(2) Drug Drug Interaction: Clarithromcyin (prescribed for ?otitis media) in patient starting on IV vincristine as part of chemotherapy for lymphoma treatment	Moderate
38	(1) Inappropriate dosage regimen: 60kg Female patient presenting with hypervolemic hyponatremia, metabolic acidosis and acute kidney injury started on a 1 litre isotonic bicarbonate infusion.	Moderate
	(2) Omission of therapy: Background of hypertension and hyperlipidemia, medication reconciliation not performed and chronic medications not resumed.	Minor
39	(1) Inappropriate dosage regimen: 50kg patient prescribed with desmopressin at 1mcg (dose is too low) for prevention of uremic bleeding prior to line insertion	Moderate
40	Control	NA

Table S3: Results from reasoning model (OpenAI's o4-mini)

**Model**	**Accuracy (SD)**	**Precision (SD)**	**Recall (SD)**	**F1 Score (SD)**
o4-mini Native	72.5% (0.63)	0.39 (0.02)	0.73 (0.01)	0.51 (0.02)
o4-mini RAG	73.6% (0.28)	0.37 (0.02)	0.71 (0.03)	0.49 (0.02)

SD: Standard Deviation

Data S1: Summary of Case Vignettes

Case No	Discipline	Brief Description of Clinical Vignettes	Number of Medications Prescribed	ATC Categories
1	Cardiology	61-year-old Malay male with history of diabetes, hypertension, chronic kidney disease, and recurrent vascular thrombotic events. He has undergone multiple surgeries, including amputations and thrombolysis. He presented with chest pain and was diagnosed with an evolved anterior myocardial infarction, for which he underwent successful percutaneous coronary intervention (PCI).	13	Electrolytes, Antithrombotics (Heparins), Insulin (fast-acting; rapid-acting and long-acting), Platelet aggregation inhibitors, proton pump inhibitors, Vasodilators, DPP-IV inhibitors, Beta-blocking agent (selective), ACE-inhibitors, B vitamins
2	Cardiology / Gastroenterology	41-year-old Malay male with a history of coronary artery disease, diabetes mellitus, KDIGO stage 3 kidney disease, and a recent episode of acute gout. He presented with epigastric pain and was admitted to hospital from the clinic. Investigations included coronary angiography with a view for percutaneous coronary intervention and gastrointestinal scopes. His medication allergies include penicillin.	13	Fast-acting insulins, parenteral nutritional products, blood glucose-lowering drugs, oral antidiabetics, lipid-modifying agents, sulfonylureas, beta-blocking agents, agents for gout, macrolides, nitroimidazole derivatives, proton pump inhibitors, antithrombotic agents.
3	Cardiology	66-year-old male with a history of minor coronary artery disease, hypertension, hyperlipidaemia, diabetes, and a chronic left caudate nucleus infarct. He presents with left lower limb swelling and pain, fever, and chest tightness. Diagnosed with an evolved inferior STEMI and left lower limb cellulitis complicated by acute kidney injury.	15	Long Acting insulin, Heparin group, low molecular weight heparins, Macrolides, Platelet aggregation inhibitors excl. heparin, Angiotensin-converting enzyme inhibitors, Sulfonylureas, Biguanides, Dipeptidyl peptidase 4 (DPP-4) inhibitors, HMG CoA reductase inhibitors, Beta-blocking agents, selective, Dihydropyridine derivatives, Proton pump inhibitors, Organic nitrates, Drugs used in erectile dysfunction
4	Cardiology	75-year-old Chinese male with a history of hypertension, hyperlipidaemia, benign prostatic hyperplasia, and a right occipital parasagittal meningioma. He presents with atypical chest pain, which is a dull ache and non-radiating. He has a history of ischemic cardiomyopathy and has undergone coronary artery bypass graft surgery and mitral valve repair.	6	Beta-blocking agents, selective, Platelet aggregation inhibitors excl. heparin, 5-alpha-reductase inhibitors, proton pump inhibitors, Alpha-adrenoreceptor antagonists, HMG CoA reductase inhibitors
5	Cardiology / Respiratory	58-year-old female admitted for acute pulmonary edema. Her past medical history includes left ataxic hemiparesis, poorly controlled diabetes mellitus, and hypertension. She exhibits shortness of breath, lower limb swelling, and is found to have an NSTEMI with underlying chronic type 2 respiratory failure likely contributed by obstructive sleep apnea.	16	Insulins and analogues, Organic nitrates, High-ceiling diuretics, Heparin group, low molecular weight heparins, Angiotensin II antagonists, Beta-blocking agents, selective, Sulfonylureas, Dipeptidyl peptidase 4 (DPP-4) inhibitors, Platelet aggregation inhibitors excl. heparin, Proton pump inhibitors
6	General Medicine	58-year-old male with a history of hypertension, admitted with dizziness and vertiginous symptoms, associated with difficulty balancing. He has a strong smoking history and had a possible posterior circulation stroke. The patient also shows signs of hyponatremia, high anion gap metabolic acidosis likely from fasting ketoacidosis, and possible polycythemia related to his smoking history.	7	Angiotensin-converting enzyme inhibitors, Platelet aggregation inhibitors excl. heparin, HMG CoA reductase inhibitors, Combination of penicillins, including beta-lactamase inhibitors
7	Endocrinology	65-year-old Indian female with a history of type 2 diabetes mellitus, hyperlipidemia, hypertension, osteoarthritis, and a past surgical procedure for a distal radius fracture. She was recently admitted for Group B streptococcus bacteremia secondary to pneumonia and experienced septic shock. She presented with an unwitnessed loss of consciousness and was	10	Angiotensin-converting enzyme inhibitors, Other antiepileptics), Sulfonylureas, Biguanides, HMG CoA reductase inhibitors, High-ceiling diuretics, Acetic acid derivatives and related substances

		found to have hypoglycemia and acute kidney injury on admission		
8	Endocrinology	68-year-old Chinese female with hypertension and osteoarthritis. She was previously admitted for severe symptomatic hypercalcemia with inappropriately normal parathyroid hormone levels, acute kidney injury, urinary tract infection, hypertensive urgency, anemia, and bilateral lower limb weakness due to cervical myelopathy. She is currently admitted from clinic for hypercalcemia management.	11	Calcitonins, Propulsives, Angiotensin II antagonists,Anilides, Antivertigo preparations, Osmotically acting laxatives, Vitamin D and analogues, Dihydropyridine derivatives
9	Endocrinology / Infectious disease / Vascular	71-year-old Chinese male with a complex medical history including ischemic heart disease, poorly controlled type 2 diabetes mellitus, peripheral vascular disease, chronic kidney disease stage 4, and moderate-severe dementia. He has been admitted multiple times for fluid overload and is currently admitted for the same issue. The patient also has infected lower limb diabetic ulcers and a painful indwelling catheter.	10	Insulins and analogues, High-ceiling diuretics, Biguanides, HMG CoA reductase inhibitors, Platelet aggregation inhibitors excl. heparin, H2-receptor antagonists, Contact laxatives
10	Ophthalmology	Female patient with right phacomorphic glaucoma and pseudophakia. She has diabetes mellitus, hypertension, hyperlipidemia, and no known drug allergies. There is a history of cataract and glaucoma, with no familial history of glaucoma.	16	Carbonic anhydrase inhibitors, HMG CoA reductase inhibitors, Alpha-adrenoreceptor agonists, Angiotensin-converting enzyme inhibitors, Sulfonylureas, Anxiolytics, Prostaglandin analogues, Sodium-glucose co-transporter 2 (SGLT2) inhibitors, Biguanides, Aldosterone antagonists)
11	Ophthalmology / Endocrinology	65-year-old female with severe thyroid eye disease (TED) related to Graves' disease. She has undergone total thyroidectomy and bilateral orbital decompression. Her current admission is for postoperative management of TED.	15	Combination of penicillins, including beta-lactamase inhibitors, Glucocorticoids, Anilides, HMG CoA reductase inhibitors, Selective COX-2 inhibitors, Thyroid hormones, Proton pump inhibitors, Aminoglycoside antibiotics
12	Ophthalmology / Infectious Disease	45-year-old male with no significant past medical history. He presented with right eye endophthalmitis and underwent various treatments, including intravitreal vancomycin and washout procedures. The patient was diagnosed with mycobacterium abscess and was treated with a combination of intravenous and oral medications.	12	Carbapenems, Macrolides, Quinolone, Other antibacterials, Anilides, Glucocorticoids, Other opioids
13	Gastroenterology	20-year-old female with primary sclerosing cholangitis and Child's Pugh A cirrhosis, chronic ulcerative pancolitis, and a history of cholelithiasis. She presented with left knee pain, fever, and symptoms of a respiratory infection. The preliminary assessment suggests a likely gout flare.	10	Immunosuppressants, Bile acids and derivatives, Proton pump inhibitors, Aminosalicylic acid and similar agents, Alpha and beta blocking agents, Quinolone antibacterials, Aminopyrazoles, Imidazole derivatives, Monobactams, Combination of penicillins, including beta-lactamase inhibitors
14	Gastroenterology	45-year-old Malay male with a history of Hepatitis C genotype 3A and Child’s B cirrhosis. He was admitted for deranged liver function tests and presented with jaundice. He presented with a needle stick injury and agrees to start treatment with Epclusa for Hepatitis C. Other active issues also includes managing possible acute cholecystitis and fluid restriction	6	High-ceiling diuretics, proton pump inhibitors, Aldosterone antagonists,Anilides<br>*Epclusa not listed
15	Gastroenterology	64-year-old Indian male with a history of Child's B8 Cirrhosis, diabetes, hypertension, hyperlipidemia, ischemic heart disease, atrial fibrillation, stage 3 chronic kidney disease, and hypothyroidism. He presents with worsening abdominal distension, lower limb edema, and reduced urine output. The patient has a history of spontaneous bacterial peritonitis and is on lifelong Ciprofloxacin for SBP prophylaxis.	10	Organic nitrates, Dipeptidyl peptidase 4 (DPP-4) inhibitors, Aminopyrazoles, Proton pump inhibitors, Platelet aggregation inhibitors excl. heparin, Alpha and beta blocking agents, Quinolone antibacterials, Cardiac glycosides, Thyroid hormones

16	Gastroenterology	77-year-old female with a history of Child's C10 cryptogenic liver cirrhosis with portal vein hypertension. She presented with diuretic-resistant ascites s/p ascitic drain with H. pylori infection.	8	Glycopeptide antibacterials, Antibiotics, Imidazole derivatives, Proton pump inhibitors, Tetracyclines, Osmotically acting laxatives, Quinolone antibacterials, Dihydropyridine derivatives)
17	General Surgery / Oncology	55-year-old Chinese male with a history of perforated duodenal ulcer, epithelial carcinoma of the salivary gland with liver metastasis, and neutropenia sepsis. His current condition includes postoperative care following laparoscopic omental patch repair of a perforated duodenal ulcer and chemotherapy for his cancer.	10	Insulins and analogues, Echinocandins, Colony-stimulating factors, Proton pump inhibitors, Anilides, Combination of penicillins, including beta-lactamase inhibitors, Other opioids, Glycopeptide antibacterials, Nucleoside and nucleotide reverse transcriptase inhibitors
18	Vascular Surgery	71-year-old Malay female with a complex medical history including end stage renal failure post living donor kidney transplant, complicated by chronic kidney disease of allograft, left renal cell carcinoma post radical nephrectomy, hyperparathyroidism with hypercalcemia, hypertension, type 2 diabetes mellitus, benign prostatic hyperplasia, and high cholesterol. She presents with right big toe pain and duskiness, leading to a diagnosis of dry gangrene of the right first toe.	18	Insulins and analogues, Beta-blocking agents, selective, HMG CoA reductase inhibitors, Immunosuppressants, Other calcimimetics, Platelet aggregation inhibitors excl. heparin, Trimethoprim and derivatives, combinations with sulfamethoxazole, Other lipid modifying agents, Folic acid, High-ceiling diuretics, Sulfonylureas, Dipeptidyl peptidase 4 (DPP-4) inhibitors, Angiotensin-converting enzyme inhibitors, Magnesium, Mycophenolic acid, proton pump inhibitors, Alpha-adrenoreceptor antagonists)
19	General Surgery / Colorectal Surgery	60-year-old Chinese male with a history of diabetes, hypertension, hyperlipidemia, polycythemia rubra vera, end-stage renal failure on hemodialysis, diverticular disease, and past surgery for perforated jejunal diverticulitis. He presented with lower abdominal pain and constipation suspicious for intestinal obstruction and later developed anal pain.	17	Osmotically acting laxatives, Anilides, Contact laxatives, Local anesthetics, Other opioids, HMG CoA reductase inhibitors, Other antiepileptics, Dipeptidyl peptidase 4 (DPP-4) inhibitors, Sulfonylureas, Propulsives, Other calcimimetics, Vasodilators used in peripheral vascular diseases, Phosphate binders, Organic nitrates, Quinolone antibacterials
20	General Surgery	46-year-old male with a history of adjustment disorder, lupus nephritis, hypertensive urgency, and chest pain. He presented with right lower abdominal pain associated with fever, diagnosed as perforated appendicitis. He underwent a laparotomy converted to open limited right hemicolectomy.	12	Potassium, Third -generation cephalosporins, Imidazole derivatives, Proton pump inhibitors, Serotonin (5HT3) antagonists, Propulsives, Anilides, Dihydropyridine derivatives, Angiotensin-converting enzyme inhibitors, Non-steroidal anti-inflammatory and antirheumatic products, coxibs, Hydralazine and diuretics, Selective COX-2 inhibitors
21	General Surgery	71-year-old male with a diagnosis of cecal diverticulitis and a small diverticular abscess. His past medical history includes benign prostatic hyperplasia, hypertension, and hyperlipidemia. The patient presented with right iliac fossa pain and had a history of abdominal pain.	11	Potassium, Third-generation cephalosporins, Imidazole derivatives, Proton pump inhibitors, Propulsives, 5-alpha-reductase inhibitors, Alpha-adrenoreceptor antagonists, Anilides, Other opioids, Sulfonamides, plain, Phenothiazines with aliphatic side-chain
22	Urology	75-year-old male with a history of ischemic colitis, hemorrhoids, benign prostatic hyperplasia (BPH), asthma, hypertension, gastritis, ischemic cardiomyopathy, and prostate cancer. He was admitted for gross hematuria and underwent bladder cystoscopy	14	Potassium, Combination of penicillins, including beta-lactamase inhibitors, Other antineoplastic agents, Platelet aggregation inhibitors excl. heparin, Beta-blocking agents,

		and cystodiathermy. His current issues include gross hematuria likely from prostate cancer and a urinary tract infection		selective, 5-alpha-reductase inhibitors, Anilides, Alpha-adrenoreceptor antagonists, Acetic acid derivatives and related substances, Organic nitrates, High-ceiling diuretics, Osmotically acting laxatives, HMG CoA reductase inhibitors
23	Urology	67-year-old male with a history of hypertension, type 2 diabetes mellitus, ischemic heart disease, benign prostatic hyperplasia, and pyelonephritis. He was admitted for pyelonephritis with persistent purulent discharge from the left loin and underwent percutaneous drainage.	12	Insulins and analogues, Platelet aggregation inhibitors excl. heparin, Beta-blocking agents, selective, Combination of penicillins, including beta-lactamase inhibitors, Other antiepileptics, Osmotically acting laxatives, Anilides, Other opioids, Alpha-adrenoreceptor antagonists,
24	Cardiology	80-year-old Chinese female with a significant history of cardiac issues, including severe cardiomyopathy and coronary artery disease, admitted with symptoms indicative of a new ST-elevation myocardial infarction (STEMI). She also has complications like acute renal failure and metabolic acidosis likely stemming from her cardiac condition and recent medical interventions.	13	Electrolytes, Antithrombotic agents, Platelet aggregation inhibitors, Proton pump inhibitors, Vasodilators used in cardiac diseases, Other vasodilators used in cardiac diseases, Cardiac therapy, Vasodilators for systemic use, Lipid modifying agents, plain, Beta blocking agents, plain, ACE inhibitors, plain, Vitamin B1, plain, and combination with Vitamin B6 and B12.
25	Emergency Medicine	17-year-old female who intentionally overdosed on over 20 fluvoxamine tablets due to emotional distress, witnessed by her family. Following the incident, she was brought to the emergency department, where she was alert, conversant, and stable, although she vomited once without expelling the pills. She expressed persistent harmful thoughts but no intent to act on them and requested psychological support. Her vital signs remained within normal limits, and her neurological exam showed no abnormalities. She was diagnosed with a fluvoxamine overdose and admitted to the ICU for monitoring, supported by telemetry and a consultation with a toxicologist.	5	Anesthetics, general, Electrolytes, Emollients and protectives, Non-steroidal anti-inflammatory drugs for topical use, Opioids
26	Endocrinology	79-year-old Chinese female with significant medical history including type 2 diabetes with complications, hypertension, hyperlipidemia, stage 3 chronic kidney disease, right knee replacement, hypothyroidism, and atrial fibrillation. She presented with severe right knee pain and swelling following a recent fall, leading to mobility issues. Lab results highlighted elevated INR and HbA1c, with clinical findings suggesting haemarthrosis exacerbated by warfarin use. The treatment plan includes fluid restriction, warfarin suspension, antibiotic administration to exclude septic arthritis, and potential knee joint aspiration.	18	Vitamin K, Proton pump inhibitors, High-ceiling diuretics, Beta-lactam antibacterials, monobactams, Glycopeptides, Blood glucose lowering drugs, insulins and analogues, Angiotensin II antagonists, plain, Antithrombotic agents, Sulfonylureas, Dipeptidyl peptidase-4 (DPP-4) inhibitors, Sodium-glucose co-transporter 2 (SGLT2) inhibitors, Lipid modifying agents, plain, Beta blocking agents, plain, Calcium channel blockers, Platelet aggregation inhibitors, Analgesics, non-opioid, Dipeptidyl peptidase-4 (DPP-4) inhibitors.
27	Family Medicine	Patient with past medical history that includes atrial fibrillation, incipient diabetic nephropathy, type II diabetes, hyperlipidemia, hypertension, and folate and vitamin B12 deficiencies. Recently hospitalized due to a urinary tract infection. Claims compliant to medications and report no side effects. The current plan is to continue with the existing medications and monitor diabetes management closely.	12	Emollients and protectives, Calcium channel blockers, ACE inhibitors, plain, Low-ceiling diuretics, thiazides, Vasodilators used in cardiac diseases, Lipid modifying agents, plain, Dipeptidyl peptidase-4 (DPP-4) inhibitors, Antithrombotic agents, direct factor Xa inhibitors, vitamins and minerals

28	Gastroenterology	80-year-old Chinese female patient with a background of hypertension, hyperlipidemia, and impaired glucose tolerance who presented with three weeks of vomiting, diarrhea, and abdominal pain, leading to hypotension requiring noradrenaline support. The patient is currently in the high dependency unit still complaining of abdominal pain. Labs show significantly deranged liver enzymes and bilirubin levels, while a CT scan revealed a 7mm distal common bile duct calculus causing duct dilation and signs of cholangitis. The primary diagnosis is Tokyo Grade III acute cholangitis due to choledocholithiasis and septic shock.	9	Prokinetic agents, Analgesics, non-opioid, Other beta-lactam antibacterials, Antibacterials for systemic use, Proton pump inhibitors, Vasopressors, Potassium-sparing agents in combination, Analgesics, non-opioid and muscle relaxants, Lipid modifying agents, plain.
29	Gastroenterology	75-year-old female with a history of type 2 diabetes, hypertension, peripheral vascular disease, end-stage renal failure on hemodialysis, long QT syndrome, and atrial fibrillation, currently not on anticoagulation due to bleeding risks. She was electively admitted for an ERCP to address an incidental common bile duct stone. Additionally, she has been experiencing a productive cough for two weeks, diagnosed as community-acquired pneumonia via a recent chest X-ray showing lower zone consolidation.	16	Antianemic preparations, Iron preparations, Antibacterials for systemic use, Other beta-lactam antibacterials, Proton pump inhibitors, Electrolytes, Analgesics, non-opioid, Opioids, Dipeptidyl peptidase-4 (DPP-4) inhibitors, Prokinetic agents, Lipid modifying agents, plain, Platelet aggregation inhibitors, Vitamin D and analog, Multivitamins with minerals, Laxatives, Phosphate binders
30	General Surgery	86-year-old Chinese female with a notable history, including a subtotal gastrectomy performed in January 2015 for a poorly differentiated adenocarcinoma of the stomach (pT3N1M0), gout treated with allopurinol, bilateral osteoarthritis of the knees, and diabetes managed with NovoMix 30. Recent evaluations via OGD (esophagogastroduodenoscopy) in June showed no signs of cancer recurrence. The patient reports a good appetite, tolerates solid foods well, and maintains energy with small, frequent meals. She is asymptomatic with an unremarkable physical examination and a pain score of zero.	9	Blood glucose lowering drugs, insulins and analogues, Proton pump inhibitors, Platelet aggregation inhibitors, Blood glucose lowering drugs excluding insulins, Blood glucose lowering drugs, SGLT2 inhibitors, Vitamin D and analogues, Agents for gout attacks.
31	Haematology	68-year-old male with a complex medical history including idiopathic thrombocytopenic purpura (ITP), ischemic heart disease with previous percutaneous coronary interventions, cerebrovascular accidents and transient ischemic attacks, chronic kidney disease stage 5 pending dialysis, hypertension, hyperlipidemia, and diabetes. He presented with recent haemoptysis and a noted petechial rash on his arms and chest, attributed to thrombocytopenia from ITP. Previously, his ITP was managed with steroids which were ceased due to induced diabetes, and currently, his response to eltrombopag is poor; however, he has shown good response to IV immunoglobulin (IVIg).	16	Antianemic preparations, Blood glucose lowering drugs, insulins and analogues, Antifibrinolytics, Corticosteroids for systemic use, Immune sera and immunoglobulins, Vasodilators used in cardiac diseases, Calcium channel blockers, Beta blocking agents, plain, Lipid modifying agents, plain, Vitamin D and analogues, Phosphate binders, Cough suppressants (excluding combinations with expectorants), Analgesics, non-opioid, Opioids, Antacids, Laxatives.
32	General Medicine	78-year-old woman with a medical history of diabetes, hypertension, glaucoma, and ischemic heart disease presented with non-vertiginous dizziness and hypertension (SBP in the 200s) after three episodes of vomiting that resolved upon hospital admission. She is alert and non-toxic, with a blood pressure of 185/95 and oxygen saturation of 99% on room air. Physical and neurological exams were unremarkable, and lab tests indicated hypokalemia and elevated creatinine levels. A CT brain scan and chest X-ray showed no acute issues. Her management includes close monitoring, dietary adjustments, potassium chloride replacement, and blood pressure control with nifedipine.	13	Electrolytes, Analgesics, non-opioid, Prokinetic agents, Mineral supplements, Calcium channel blockers, Blood glucose lowering drugs excluding insulins, Dipeptidyl peptidase-4 (DPP-4) inhibitors, Platelet aggregation inhibitors, Proton pump inhibitors, Lipid modifying agents, plain, Beta blocking agents, plain, Vitamin D and analogues.

33	General Medicine / Critical Care	65-year-old Indian male with a medical history of Child's A liver cirrhosis due to chronic hepatitis B, post-pyloric ulcer surgery, and recent right knee septic arthritis, is currently managing complications including fluid overload-related acute decompensated type 2 respiratory failure and KDIGO 3 acute kidney injury requiring dialysis. Admitted following septic shock with episodes of hypotension, he was stabilized in the medical intensive care unit with inotropic support and non-invasive ventilation.	15	Electrolytes, Beta-lactam antibacterials, including penicillins, Mineral supplements, Mineral supplements, Glycopeptides, Corticosteroids for systemic use, High-ceiling diuretics, Nucleoside and nucleotide reverse transcriptase inhibitors, Proton pump inhibitors, Beta blocking agents, plain, Antithrombotic agents, direct factor Xa inhibitors, Sulfonamides, plain, Laxatives, Analgesics, non-opioid, Mucolytics.
34	General Medicine	72-year-old Chinese male with multiple chronic conditions including type 2 diabetes, hyperlipidemia, hypertension, osteoarthritis, chronic glomerulonephritis-induced CKD, and alcoholic liver cirrhosis presented with generalized myalgia and weakness due to an accidental double dosing of statins, leading to rhabdomyolysis. Additional complications include fluid overload and heart failure with a mid-range ejection fraction. His treatment has been adjusted to manage these conditions, with a suspension of statins, fibrates, and dapagliflozin to mitigate further complications. He is under close monitoring with regular vitals, intake/output charting, and medication adjustments to manage fluid overload and support liver and kidney functions.	14	Electrolytes, Proton pump inhibitors, Other beta-lactam antibacterials, Antibacterials for systemic use, High-ceiling diuretics, Potassium-sparing agents, Beta blocking agents, plain, Blood glucose lowering drugs, SGLT2 inhibitors, Sulfonylureas, Laxatives, Contact laxatives, Lipid modifying agents, plain, Lipid modifying agents, fibrates, Antithrombotic agents.
35	Infectious Disease	82-year-old Chinese female with a history of hypertension, hyperlipidemia, diabetes, right hip osteoarthritis, methicillin-sensitive Staphylococcus aureus (MSSA) bacteremia, and triple vessel disease currently presents with low hemoglobin and a gradual functional decline reported over the past year. She experiences intermittent fever spikes and lethargy, alongside a reduced exercise tolerance. The patient denies pain, cough, or shortness of breath and exhibits stable vital signs with poor dental hygiene and consistent weakness on the right side compared to the left, as noted by her son. Lab results indicate anemia with hemoglobin at 8.7 g/dL, mild hyponatremia, and subclinical hypothyroidism.	11	Electrolytes, Proton pump inhibitors, Platelet aggregation inhibitors, H2-receptor antagonists, Laxatives, Contact laxatives, Calcium channel blockers, Drugs for urinary frequency and incontinence, Iron preparations, Antifungals for systemic use, Mineral supplements.
36	Neurology	72-year-old male with a history of hypertension and recent stroke symptoms during a trip to Indonesia is being treated for an acute left middle cerebral artery (MCA) infarction confirmed by CT, showing a hyperdense clot and good collateral circulation. His current management includes endovascular therapy (EVT) and medication regimen including IV nicardipine for blood pressure control, and integrilin infusion to maintain vascular patency, with strict neurological monitoring. He was deemed unsuitable for TPA due to MRI findings.	7	Platelet aggregation inhibitors, Proton pump inhibitors, Electrolytes, Calcium channel blockers, Lipid modifying agents, plain, Platelet aggregation inhibitors, Platelet aggregation inhibitors.
37	Medical Oncology	70-year-old Chinese male with a history of chronic ischemic heart disease, type 2 diabetes with nephropathy, chronic kidney disease stage 3, gout, and a history of otitis media is currently being treated for newly diagnosed stage IV aggressive large B-cell lymphoma. The patient presents with left flank pain, scrotal swelling, significant weight loss, loss of appetite, and worsening shortness of breath on exertion, alongside a left-sided upper rib pain. His condition includes complications from lung cancer in a family member and a 40 pack-year	22	Blood glucose lowering drugs, insulins and analogues, Electrolytes, Antiemetics and antinauseants, Antineoplastic agents, Antineoplastic agents, Antineoplastic agents, Antineoplastic agents, Antineoplastic agents, Antihistamines for systemic use, Antineoplastic and immunomodulating agents, Antineoplastic agents, Colony

		smoking history. Diagnostic workups have identified a retroperitoneal mass and involvement of the adrenal gland and omentum.		stimulating factors, ACE inhibitors, plain, Blood glucose lowering drugs, SGLT2 inhibitors, Blood glucose lowering drugs excluding insulins, Agents for gout attacks, Lipid modifying agents, plain, Platelet aggregation inhibitors, Calcium channel blockers, H2-receptor antagonists, Mucolytics, Potassium-binding agents, Macrolides, Analgesics, non-opioid.
38	Renal	65-year-old white male with a complex medical history including hypertension, dyslipidemia, hepatic steatosis, renal cysts, and previous surgery for a gluteal lipomatous tumor is currently admitted with central chest discomfort, orthopnea, and worsening exertional dyspnea. His symptoms have been progressive over the past two weeks, and he has developed bilateral lower limb swelling and abdominal bloating, suggestive of fluid overload. Lab results indicate acute kidney injury (KDIGO 3), hypervolemic hyponatremia, thrombocytopenia, and cholestatic liver function tests. He is being managed with intravenous furosemide for fluid overload and tazocin for suspected infection.	7	High-ceiling diuretics, Electrolytes, Beta-lactam antibacterials, including penicillins, Proton pump inhibitors, Laxatives, Contact laxatives, Cough suppressants (excluding combinations with expectorants).
39	Respiratory Medicine	52-year-old Chinese female with systemic lupus erythematosus and end-stage renal failure underwent bilateral radical nephrectomy for multifocal renal cell carcinoma, complicated by retroperitoneal hematoma and pulseless electrical activity requiring tracheostomy. Post-operatively, she developed Stenotrophomonas maltophilia bacteremia and type 2 myocardial infarction. Current management includes nasojejunal feeding to reduce aspiration risk, intravenous ceftazidime for bacteremia, and monitoring for potential dialysis. She remains stable with ongoing hemoptysis and raised troponins but no acute ischemic changes on ECG.	13	Other beta-lactam antibacterials, including penicillins, Beta blocking agents, plain, Vasodilators for systemic use, Pituitary and hypothalamic hormones and analogues, Platelet aggregation inhibitors, Proton pump inhibitors, Angiotensin II antagonists, plain, Calcium channel blockers, Alpha-adrenoreceptor antagonists, Beta blocking agents, plain, Other antiepileptics, Sympathomimetics, Emollients and protectives.
40	Respiratory Medicine	61-year-old Caucasian female with chronic obstructive pulmonary disease (COPD), hyperlipidemia, and heart failure was admitted for cough, shortness of breath, vomiting, and lethargy, presenting with decompensated type 2 respiratory failure. Treatment included non-invasive ventilation, antibiotics (ceftazidime and levofloxacin), and steroids (IV hydrocortisone) to manage an exacerbation likely caused by a viral infection.	11	Corticosteroids for systemic use, Proton pump inhibitors, Beta-lactam antibacterials, including penicillins, Macrolides, Sympathomimetics, anticholinergics, Inhalants for obstructive airway diseases, Electrolytes, Antivirals for systemic use, Analgesics, non-opioid, Drugs for obstructive airway diseases, combinations, Lipid modifying agents, plain.
