# Supplementary material for: Large language model as clinical decision support system augments medication safety in 16 clinical specialties
Source: Cell Rep Med. 2025 Sep 24;6(10):102323. doi: 10.1016/j.xcrm.2025.102323 (PMC12629785; doi:10.1016/j.xcrm.2025.102323)
Supplement: Document S2. Article plus supplemental information [file mmc2.pdf]

Cell Reports Medicine

Article

Large language model as clinical decision support system augments medication safety in 16 clinical specialties

Graphical abstract

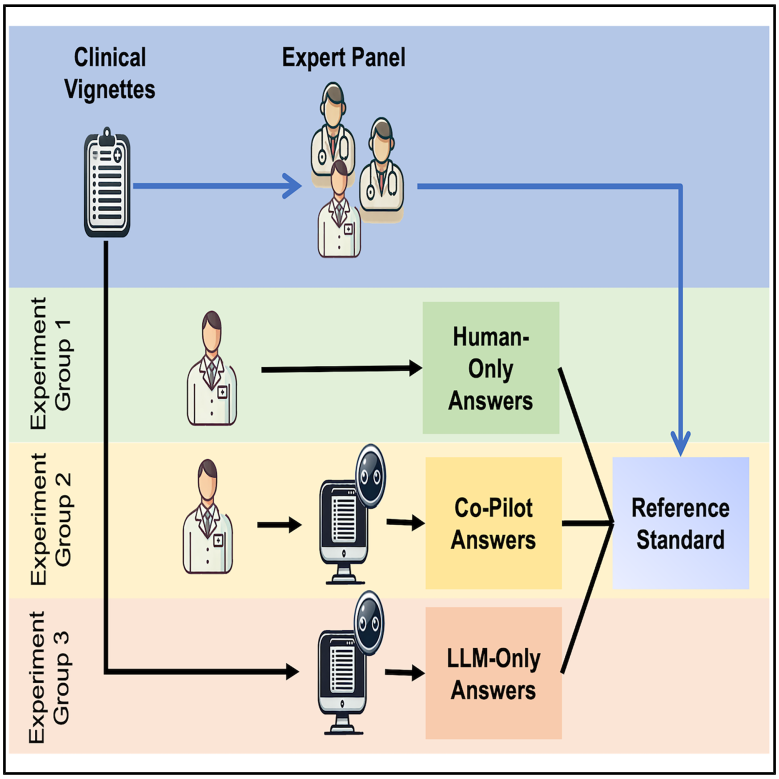

Highlights

- Evaluated LLMs in identifying drug-related problems across complex cases
- Developed and validated RAG-LLM to enhance reasoning in clinical decision support tasks
- Demonstrated varying LLM accuracy in medication safety-related reasoning
- LLM use in real-world clinical practice

Authors

Jasmine Chiat Ling Ong, Liyuan Jin, Kabilan Elangovan, ..., Tat Ming Ng, Nan Liu, Daniel Shu Wei Ting

Correspondence

daniel.ting.s.w@singhealth.com.sg

In brief

Ong et al. explored how large language models (LLMs) support safe prescribing by detecting drug-related problems across clinical scenarios. The study demonstrated that LLMs augmented performance of pharmacists and improved overall accuracy of medication chart review. However, limitations in clinical generalizability, bias, and prompt design must be addressed before routine use.

Ong et al., 2025, Cell Reports Medicine *6*, 102323
October 21, 2025 © 2025 The Author(s). Published by Elsevier Inc.
https://doi.org/10.1016/j.xcrm.2025.102323

CellPress

Cell Reports Medicine

CellPress OPEN ACCESS

Article

Large language model as clinical decision support system augments medication safety in 16 clinical specialties

Jasmine Chiat Ling Ong,[1,2,14] Liyuan Jin,[2,3,4,14] Kabilan Elangovan,[3,4] Gilbert Yong San Lim,[3,4] Daniel Yan Zheng Lim,[5,6] Gerald Gui Ren Sng,[6,7] Yu He Ke,[8] Joshua Yi Min Tung,[6,9] Ryan Jian Zhong,[1] Christopher Ming Yao Koh,[1] Keane Zhi Hao Lee,[1] Xiang Chen,[1] Jack Kian Ch'ng,[10] Aung Than,[11] Ken Junyang Goh,[12] Chuan Poh Lim,[1] Tat Ming Ng,[13] Nan Liu,[2] and Daniel Shu Wei Ting[2,3,4,15,*]

[1]Division of Pharmacy, Singapore General Hospital, Singapore, Singapore
[2]Duke-NUS Medical School, Singapore, Singapore
[3]Singapore National Eye Centre, Singapore Eye Research Institute, Singapore, Singapore
[4]Singapore Health Services, Artificial Intelligence Office, Singapore, Singapore
[5]Department of Gastroenterology, Singapore General Hospital, Singapore, Singapore
[6]Data Science and Artificial Intelligence Lab, Singapore General Hospital, Singapore, Singapore
[7]Department of Anesthesiology, Singapore General Hospital, Singapore, Singapore
[8]Department of Endocrinology, Singapore General Hospital, Singapore, Singapore
[9]Department of Urology, Singapore General Hospital, Singapore, Singapore
[10]Department of Vascular Surgery, Singapore General Hospital, Singapore, Singapore
[11]Department of Internal Medicine, Singapore General Hospital, Singapore, Singapore
[12]Department of Respiratory and Critical Care Medicine, Singapore General Hospital, Singapore, Singapore
[13]Division of Pharmacy, Tan Tock Seng Hospital, Singapore, Singapore
[14]These authors contributed equally
[15]Lead contact
*Correspondence: daniel.ting.s.w@singhealth.com.sg
https://doi.org/10.1016/j.xcrm.2025.102323

SUMMARY

Large language models (LLMs) have emerged as tools to support healthcare delivery, from automating tasks to aiding clinical decision-making. This study evaluated LLMs as alternative to rule-based alert systems, focusing on their ability to identify prescribing errors. This was designed as a prospective, cross-over, open-label study involving 91 error scenarios based on 40 clinical vignettes across 16 medical and surgical specialties. We developed and validated five LLM models using a retrieval-augmented generation framework. The best-performing model evaluated three different implementation strategies: LLM-based clinical decision support system (CDSS) alone, pharmacist plus LLM-based CDSS (co-pilot), and pharmacist alone. The co-pilot arm demonstrated the best performance with an accuracy of 61% (precision 0.57, recall 0.61, and F1 0.59). In detecting errors posing serious harm, the co-pilot mode increased accuracy by 1.5-fold over the pharmacist alone. Effective LLM integration for complex tasks like medication chart reviews can enhance healthcare professional performance, improving patient safety.

INTRODUCTION

Medical errors remain a formidable challenge for healthcare institutions all around the world and are the third leading cause of mortality in the United States. Medication-related errors account for an average of 21% of hospital readmissions, with 69% of these admissions considered preventable.[1,2] Medication errors can potentially result in prolonged hospitalization stay and elevated risk for morbidity and mortality as well as an increase in healthcare spending.[3] This translates to high economic burden of medication errors, amounting up to USD$40 billion in the United States and £750 million in England per year.[3,4] In an acute care setting, medication errors can occur at any stage of the medication use process: medication prescribing, dispensing, and administration and patient monitoring. A vast majority of errors occur at the prescribing stage, accounting for 70% of errors that result in adverse patient events.[5] Halting errors at this stage is critical in preventing perpetuation of the error downstream and eventually reaching the patient.

Clinical decision support systems (CDSSs) have become a cornerstone of modern healthcare systems as a direct aid to clinical decision-making. A CDSS is intended to improve healthcare delivery by enhancing medical decisions with targeted clinical knowledge, patient information, and other health information.[6] CDSSs have demonstrated utility in reducing prescribing errors or adverse events when integrated with electronic health records and computerized provider order entry systems.[7] In particular categories of prescribing errors such as drug-drug interactions,

© 2025 The Author(s). Published by Elsevier Inc.
This is an open access article under the CC BY license (http://creativecommons.org/licenses/by/4.0/).

Table 1. Demographics of patient case vignettes

Variable	Case vignette subjects, no. (%), (*N* = 40)	
Age, median (interquartile range, IQR) [range], years	65.5	(58.5, 72), [17–86]
Gender		
Female	15	(37.5)
Male	25	(62.5)
Admitting discipline		
Medical	27	(67.5)
Surgical	13	(32.5)
Charlson's comorbidity index, median (IQR) [range]	3	(2, 5), [0–10]
Co-morbidities		
Myocardial infarction	14	(35)
Congestive heart failure	6	(15)
Peripheral vascular disease	2	(5)
CVA or TIA	5	(12.5)
Dementia	2	(5)
COPD	1	(2.5)
Peptic ulcer disease	1	(2.5)
Severe liver disease	6	(15)
Complicated diabetes mellitus	19	(47.5)
Moderate to severe CKD	16	(40)
Solid cancer	6	(15)
Hematological cancer	1	(2.5)
Medication reconciliation performed		
Yes	33	(82.5)
No	7	(17.5)
No. of medication orders, median (IQR) [range]	13	(10, 15), [5–22]
MCRI score, median (IQR) [range]	40	(30, 52), [14–83]

the real-world implementation of CDSS was effective in reducing the incidence of errors.[8] However, a vast majority of current systems are rule based, resulting in the generation of voluminous and clinically irrelevant alerts to users.[9] Over time, safety alerts are ignored and overwritten by physicians in as high as 95%. "Alert fatigue," coupled with heavy patient load and high cognitive burden from the need to process a massive amount of patient health information, poses barrier to effective adoption and utilization of CDSS systems.[10–12] Greater personalization of alerts, more intuitive interfaces, and lesser emphasis on disruptive alerts show promise in improving current state of CDSS.

The growing capabilities of large language models (LLMs) in medical tasks are becoming more apparent. LLMs are advancing in handling such tasks, particularly those that do not require extensive specialized expertise.[13,14] This includes simplifying administrative duties like composing medical letters, creating summaries upon patient discharge using information from electronic health records (EHRs) to semi-autonomous decision-making support for managing operating theaters.[15,16] Finally, LLM-powered healthcare chatbots are capable of providing patients and health professionals with highly professional-sounding, accurate, and personalized responses to medical queries.[17,18] Healthcare systems are facing critical shortage of healthcare professionals compounded by a mounting issue of healthcare professional burnout.[19–21] LLM-powered solutions are well poised to improve operational efficiency and standards of patient care when trained with the right data, robustly evaluated in the clinical setting with a deployment strategy armed with appropriate safety measures.

Prior published studies have developed LLM-based tools to support various clinical applications and domains.[22–24] Generative AI tools, such as LLMs, demonstrate potential to reduce medication-related harm when integrated into the medication use process as clinical decision support tools. LLMs were designed to perform specific tasks such as identifying inappropriate benzodiazepine prescriptions, classifying adverse drug reactions to chemotherapy, and predicting potential drug-drug interactions. The use of LLMs as an innovative substitute to current rules-based CDSS for medication review, however, has not been described. In this study, we propose a modified clinical model of care, leveraging upon LLMs as a tool to improve safety of medication use in acute care settings. The objective of this study was to evaluate the performance of LLM-based tools in correctly identifying prescribing errors and provision of clinically aligned recommendations to rectify identified errors across different medical disciplines. This has implications for the potential integration of such tools in healthcare and attendant improvements in patient safety and quality of care.

RESULTS

Six pharmacists of varying levels of work experience participated in this study. Four participants were junior pharmacists with post-licensure practice experience of between 2 and 5 years, while 2 participants were senor pharmacists with >10 years of post-licensure practice experience. The case vignettes are representative of complex clinical case scenarios with multiple co-morbidities and problem lists (Table 1). The expert panel determined that 31.9% (29 out of 91) error scenarios were capable of causing serious harm and 52.7% (48 out of 91) capable of inflicting moderate harm while the remaining 15.4% (14 out of 91) were rated as capable of causing minor or no harm. The three most common drug-related problems (DRPs) in the case scenarios were inappropriate dosage regimen that arose due the need for dose, frequency or duration adjustment; adverse drug reactions requiring change in medication or reversal agents (this also includes prescribing medications that are contraindicated based on patient profile), and significant drug-drug interactions requiring change in medication or therapeutic drug monitoring. The median unique medications across anatomical therapeutic chemical classes was 12 (IQR 5–16) per case vignette, suggesting that our constructed cases were complex and demonstrative of patients seen at a tertiary healthcare institution.

Comparative performance of native and RAG-LLMs

We evaluated outputs from native LLMs and retrieval augmented generation (RAG)-LLMs. Mean performance of all models were presented in Figure 1. Of the native models, Gemini Flash scored the lowest while Claude 3.5 Sonnet performed the best on

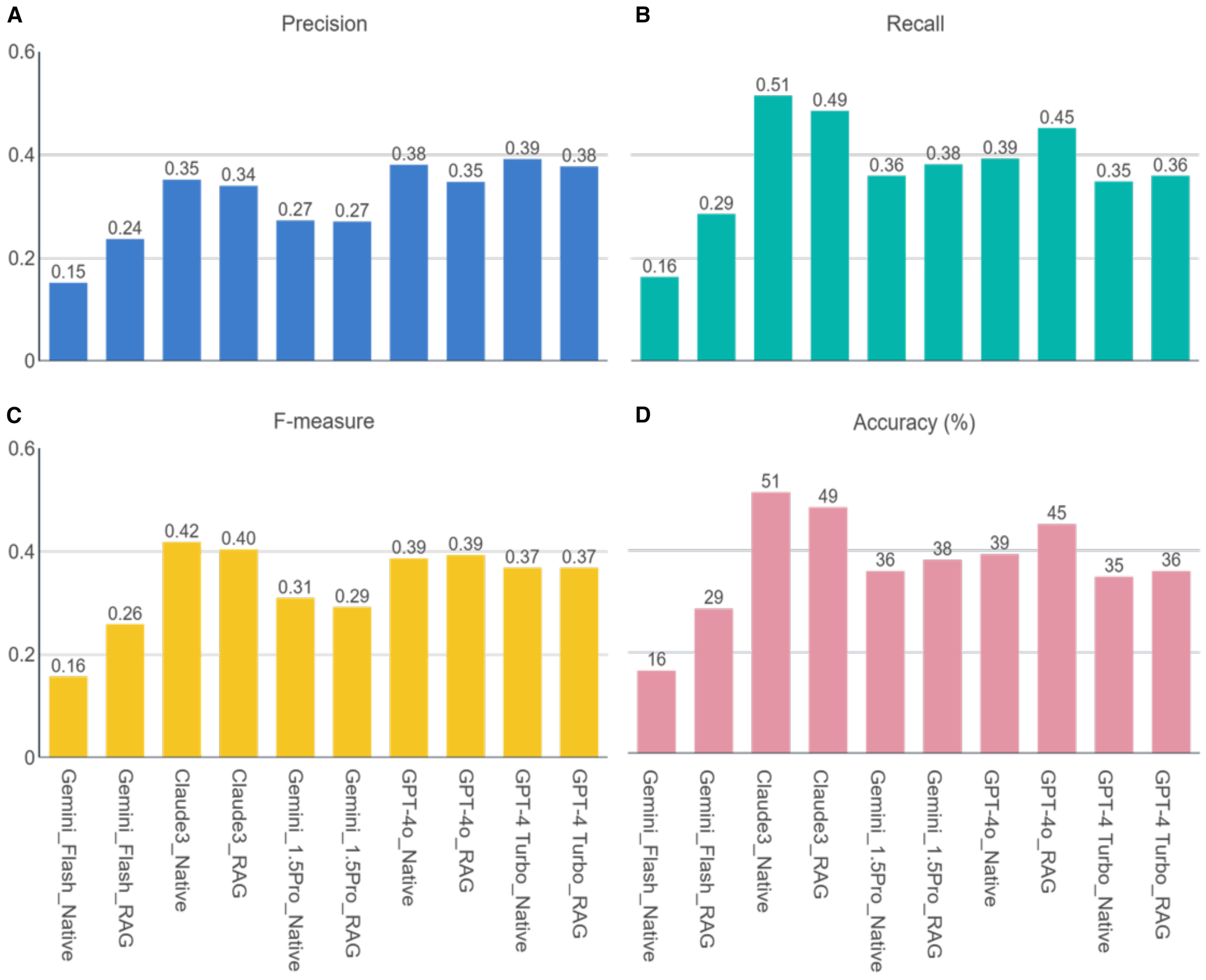

Figure 1. Comparative performance of respective native and RAG-LLMs
Performance of different LLMs in terms of (A) precision, (B) recall, (C) F-measure, and (D) accuracy.

measures of recall (0.16 vs. 0.51), precision (0.15 vs. 0.35), F1 (0.16 vs. 0.42), and overall accuracy (16% vs. 51%). Applying a RAG framework improved accuracy and recall of all models except for Claude 3.5 Sonnet. When compared against the native model, the RAG model demonstrated difference in accuracy of −2% (51% vs. 49%), recall of −0.02 (0.51 vs. 0.49), precision of −0.01 (0.35 vs. 0.34), and F-measure of −0.02 (0.42 vs. 0.40). A secondary analysis using a reasoning model, OpenAI's o4-mini, was performed with results reported in Table S3.

Reproducibility as evaluated using BERTscore (bidirectional encoder representations from transformer)ML, cosine similarity, and BLEU (bilingual evaluation understudy) score demonstrated high degree of reproducibility across LLM responses when prompted repeatedly. Claude 3.5 Sonnet demonstrated high degree of semantic reproducibility, scoring an average of 0.84, 0.947, and 0.570 for BERT score, cosine similarity, and BLEU score, respectively.

Comparative performance of different modes of delivery

The Claude 3.5 Sonnet (native) model was the adopted model in LLM-CDSS due to the best overall performance during validation. There was no statistically significant differences in mean accuracy between different modes of care: co-pilot (pharmacist with LLM-CDSS), human only (pharmacist without LLM-CDSS), and LLM only ($p = 0.38$) (Figure 2A). The mean accuracy in identifying DRPs increased by 32.6% in co-pilot mode when compared against human alone (61%, SD 17.7 vs. 46%, SD 21.1). Precision, recall, and F1 score were all higher in co-pilot mode when compared against human alone (0.57 vs. 0.52, 0.61 vs. 0.46, and 0.49 vs. 0.45) (Figure 2B). Performance under various modes was not significantly correlated with increasing case complexity as measured with the medication regimen complexity index (MCRI) score (Figure 2C).

In terms of DRP categories (Figure 3), co-pilot mode showed higher accuracy of DRP identification when compared against

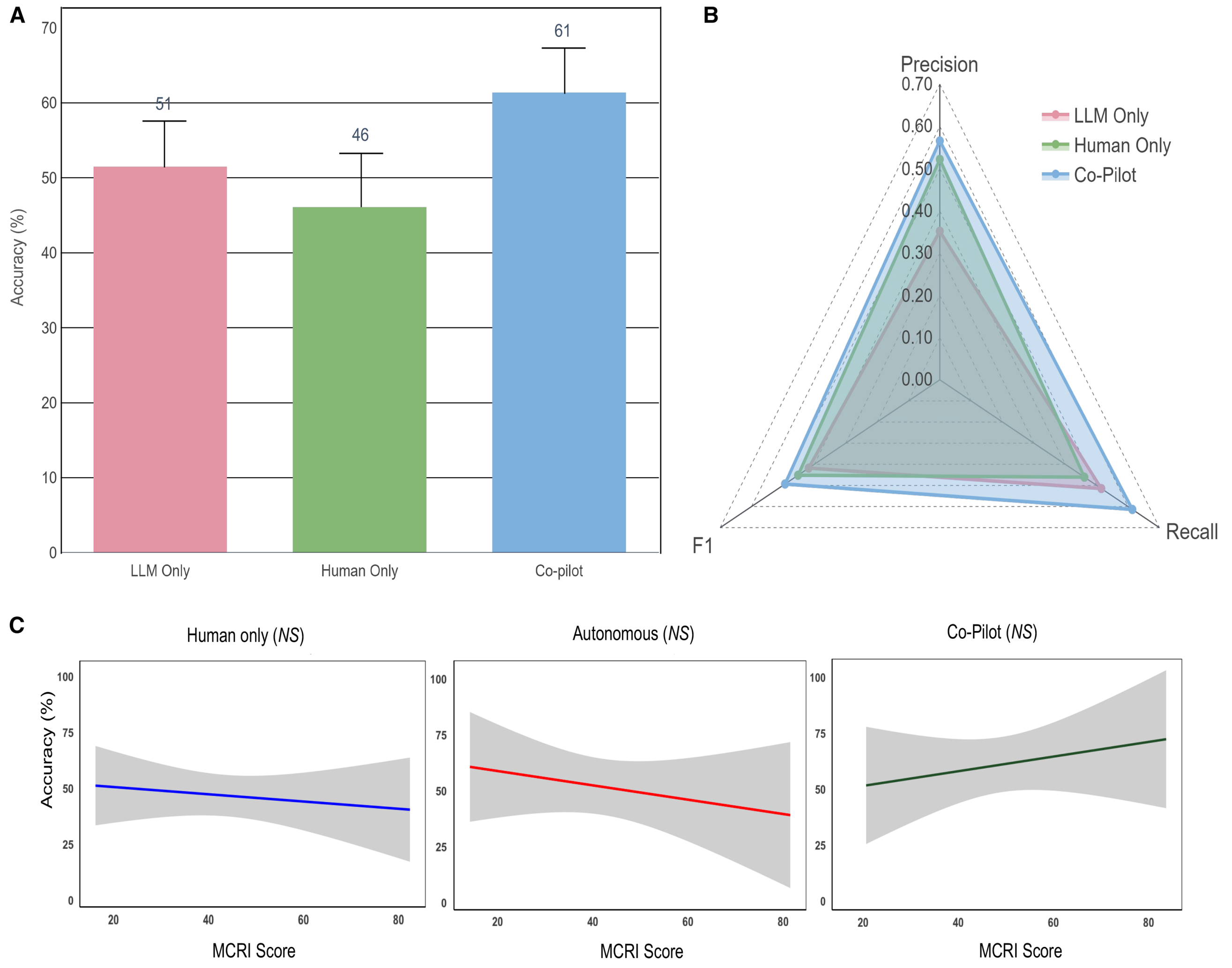

Figure 2. Performance of different modalities of implementation

(A) Chart showing comparative accuracy of different modes.

(B) Spider diagram showing relative precision, recall, and F1 scores of different modes.

(C) No correlation between MCRI score and accuracy in identifying medication-related problems. Higher MCRI scores suggest greater complexity of cases. The three plots are presented as correlation coefficient −0.0777 (95% CI: −0.3272 to 0.182) for human only, −0.1389 (95% CI: −0.4353 to 0.1848), and 0.1456 (95% CI: −0.2081 to 0.4657). The solid line represents respective regression lines. The translucent band around the regression line represents the 95% CI. NS indicates no statistical significance ($p > 0.05$).

human alone across all categories except for inappropriate dosage regimen. Accuracy was seen to decline in co-pilot mode when an inappropriate dosing regimen was encountered in the clinical vignettes (52% vs. 43%). LLMs alone were capable of catching 52% of serious errors, while that improved with co-pilot mode to 66%.

DISCUSSION

Previous work evaluating the utility of LLMs in medicine focuses heavily on reporting model performance while few prospective studies have evaluated the practical performance of healthcare professionals when LLMs are incorporated as part of a clinical workflow.[25–27] In this study, we provide a proof of concept of an application of LLM-based CDSS, designed to improve safety of medication use and prescribing in an acute care practice setting. Our findings are based on 90 different errors and DRPs, embedded within 40 clinical vignettes. Different modes of delivery were tested in a cross-over, open-label study design. We report greater accuracy in DRP identification by 32% when used in a co-pilot mode by pharmacists as compared to pharmacists alone without the tool. Similarly, the accuracy of co-pilot mode in identifying DRPs surpassed that of LLM alone. In co-pilot mode, two-thirds of DRPs with potential for causing serious harm were detected. This study evaluates LLM in assessing prescription appropriateness using patient-specific textual information, which is the most common format encountered by pharmacists during medication reviews.

CellPress
OPEN ACCESS

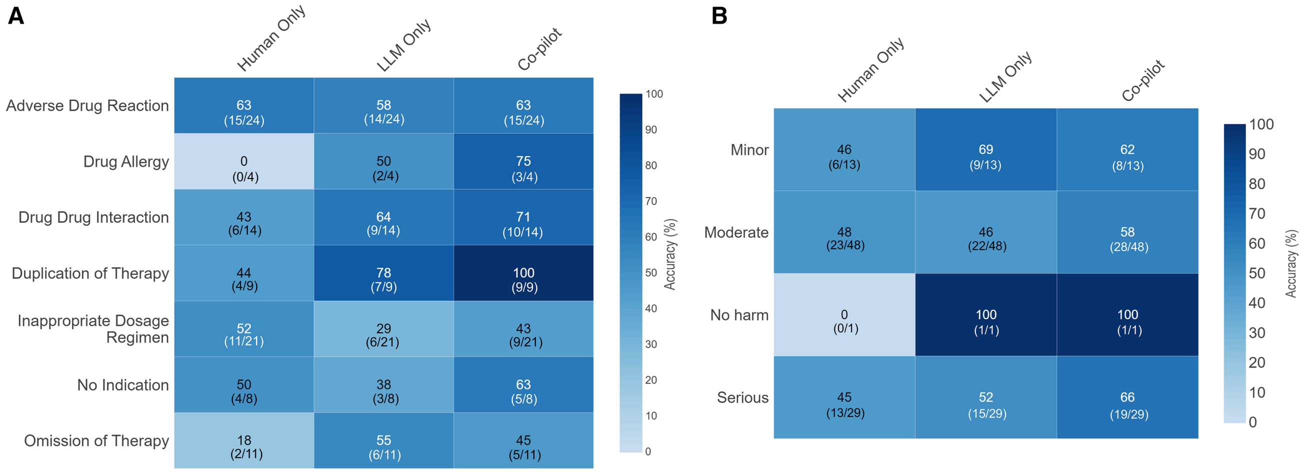

Figure 3. Performance across different categories of DRPs and different severities of potential harm

(A) Heatmap showing mean accuracy of different modes in various categories of DRP.

(B) Heatmap showing mean accuracy of different modes for DRPs of varying severity of harm.

Data are represented as mean ± SD.

In contrast to prior work that focused on narrow tasks using limited input data,[26] our study used a combination of patient information and medication details to leverage the general reasoning capabilities of LLMs for evaluating clinical appropriateness. We compared three modes of implementation: LLM alone, human alone, and a collaborative setting where the LLM assisted the user. This approach provides a more comprehensive and realistic understanding of how LLMs can be integrated into clinical practice, addressing limitations of previous studies that lacked direct comparisons or real-world application scenarios.[28,29] In addition, previous studies that included a comparator often evaluated LLMs against human experts under the assumption that LLMs will be deployed autonomously in clinical settings.[30] In contrast, our study examined three configurations: human expert-only, LLM-only, and a co-pilot approach. This provides a more complete and practical understanding of how these models perform when used alongside clinicians in real-world settings.

Our results suggests that, for complex reasoning clinical tasks such as medication chart review, LLMs can potentially augment human performance. However, we observed that, in the co-pilot mode, performance declined in 1 key category of DRP: inappropriate dosage regimen. Variability in medication dosing regimen across different geographical regions and academic institutions has been well described.[31,32] In our study, we evaluated the performance of Claude Sonnet 3.5, a commercially available LLM, that was pre-trained on a broad dataset not restricted to carefully curated medication content or the most up-to-date drug dosing guidelines.[33] These results are consistent with those reported by Yu et al., who found that inaccurate AI predictions negatively impacted the performance of radiologists.[34] The impact of AI-based decision support tools on user performance has been shown to be heterogeneous, suggesting a need for personalized approaches in the design of clinician-AI interfaces.[35] The differences between clinician and medical AI decisions are due to a basic mismatch in how each defines the clinical problem. AI systems developed for healthcare applications often lack a clear explanation of how decisions are derived. Although explainable AI tools aim to reveal the reasoning behind an AI's output, they often use technical explanations that remain confusing or are misleading to users.[35] This lack of clarity can make it hard for clinicians to trust or effectively use these systems. In contrast, LLMs with conversational interfaces allow clinicians to ask questions and receive context-specific explanations in plain language. We believe that this interactive and adaptive communication can strengthen the partnership between clinicians and AI, making the technology more useful and trustworthy in clinical decision-making.

CDSSs can be broadly classified into two distinct categories: knowledge-based (KB) and AI-driven systems. KB CDSSs rely on a set of rules and associations derived from clinical guidelines or expert opinions. They function by matching patient-specific data with a knowledge base and providing recommendations or alerts.[36] AI-driven systems, on the other hand, are capable of analyzing large datasets, identify patterns, and make predictions or recommendations based on new data without predefined rules.[7] Our LLM-based CDSS is an interesting approach to an AI-driven system. Widespread adoption of AI-driven CDSS systems is met with barriers, including lack of transparency, uncertainty relating to the evidence, lack of trust in the system, and disruptions to clinical workflow that add time to routine clinical practice.[37,38] To date, ML-based CDSSs are fairly narrow in application, most being domain specific. LLMs grounded with contextual knowledge present various advantages over ML-based models, including the ability to integrate and process vast amounts of varied data types, including unstructured clinical texts;easily update the clinical knowledge corpus without the need for explicit retraining; and offer explanations in natural language that are more comprehensible to human practitioners. In our study, we leveraged upon the generalist capability of LLMs and tested the model in a wide variety of clinical scenarios from different disciplines and included a wide range of medication classes.

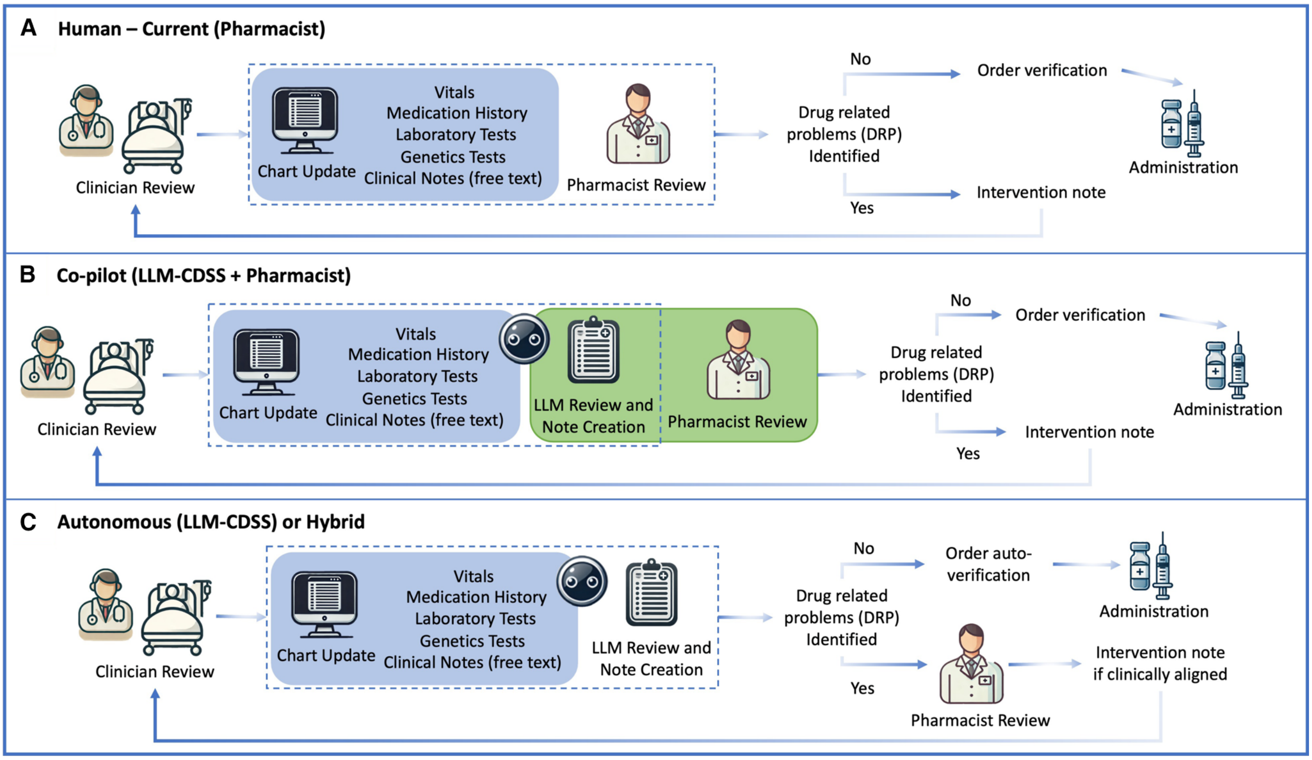

Figure 4. Proposed clinical flow integrating LLM-based CDSS in medication review in acute care setting

(A) Describes current state, whereby patient information and medication orders are updated in electronic health record system after clinician review of the patient. In a sequential fashion, the pharmacist reviews the medication order and patient chart and intervenes with prescriber if DRP is identified.

(B) LLM-CDSS acts as a co-pilot to summarize patient information, performs a medication review, and creates a note if DRP is identified for pharmacist to preview.

(C) Institutions with no access to trained clinical pharmacists or insufficient pharmacy staffing may use LLM-CDSS as an initial screening process for DRPs. Medication orders with DRP will be highlighted for pharmacist review and intervention.

However, despite the strengths of LLM-based CDSS, the potential pitfalls of LLM-CDSS tools must be recognized. Although contextually grounded models show promise in reducing hallucinations and improve clinical accuracy of LLMs, our findings suggest that even supplementing an LLM with a knowledge base through the use of RAG did not yield comparatively better results (Figure 2). This might be explained by various reasons. First, our study tested RAG-LLMs on a small number of vignettes and explored a limited range of scenarios. A bigger sample size of scenarios is required to validate this observation. Second, LLMs may demonstrate reluctance to fully accept newly retrieved knowledge, especially when it conflicts with the model's pretraining data. When conflicting knowledge is integrated, the LLM often produces ambiguous or inconsistent recommendations. Moreover, with regards to LLM-RAG-based CDSSs, RAG systems face challenges due to the lack of a state-of-the-art pipeline or retrieval techniques that have been universally established. This necessitates the creation of highly tailored RAG pipelines for each specific use case, which can be resource intensive and technically demanding. Finally, suboptimal performance of the RAG pipeline in our study can be attributed to the complexity of clinical reasoning required for medication-related tasks, which often exceeds the capabilities of general-purpose retrieval and generation mechanisms. Conflicts between retrieved and pre-trained knowledge and retrieval noise may have compromised the integration of relevant clinical context. Barriers to adopting AI-driven CDSSs, including LLM-based systems, also remain substantial. These systems are met with concerns about their lack of transparency, uncertainty surrounding the evidence base, and disruptions they may cause to clinical workflow, which can add time to routine clinical practice. As such, further refinements and reliable methodologies are required before AI-driven CDSSs can achieve widespread adoption in healthcare.

KB CDSSs are implemented for various purposes and often tailored to the peculiar needs of the healthcare facility. Regarding their efficacy in reinforcing prescribing safety, various studies have shown that KB CDSSs can significantly contribute to the reduction of prescription errors and improve prescribing practices. In a meta-analysis of 68 trials evaluating CDSSs on physician prescribing, positive behavior improved by 4.4% (95% confidence interval [CI] 2.6%–6.2%) with the deployment of KB CDSSs.[36] AI-based CDSSs have similarly demonstrated positive outcomes on prescribing safety and potential cost savings from DRP avoidance.[37,38] These trials underscore the vital role of CDSSs in enhancing prescribing safety, whether through rule-based systems or through advanced AI-driven analytics.

CellPress
OPEN ACCESS

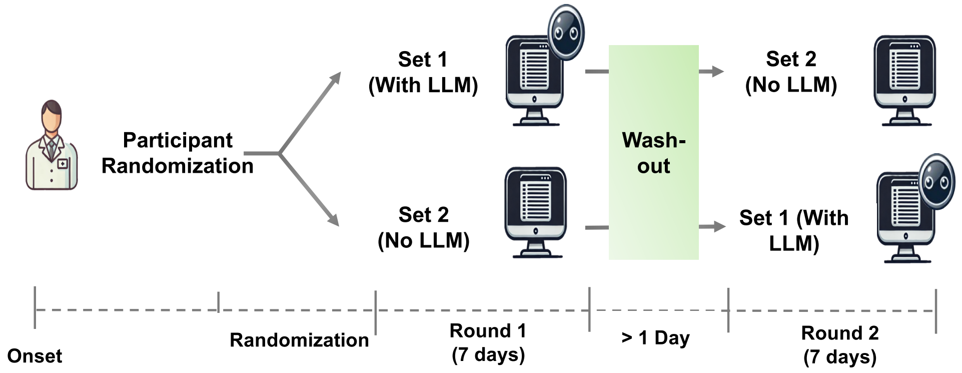

Figure 5. An overview of the cross-over study flow

However, the successful implementation and effectiveness of these systems depend on several factors, including system design, user interface, integration into clinical workflows, and training of healthcare professionals. LLM-based CDSSs present with unique advantages such as the ability to process and interpret large volumes of unstructured clinical data, adapt to evolving medical knowledge, and provide personalized treatment recommendations.[39,40] We foresee that LLM-based CDSSs can be integrated into clinical workflow in co-pilot mode to augment the performance of pharmacists (Figure 4). In addition, we envisage that, in low resource settings with manpower constraints, an autonomous/hybrid mode of AI delivery may plug current gaps in clinical services. In our study, we were unable to perform a direct comparison of our LLM-based CDSS against traditional KB CDSS. This is due to high variability of CDSS design across different healthcare institutions, making standardization of KB CDSSs unfeasible. Another reason was that the inclusion of traditional KB CDSS tools could have introduced confounding factors, such as differences in pharmacists' familiarity with the tools and the potential influence of automation bias.[41]

The limitations of our study are as follows. (1) DRPs identified from clinical scenarios were limited to 5 categories, and this might limit generalizability in clinical cases with other DRP categories. Test scenarios were adapted from errors captured by pharmacists and may represent bias toward scenarios that reach intervention and reporting thresholds. (2) The number of fictional clinical scenarios was limited, notwithstanding that each fictional scenario was designed to be highly complex in order to allow for initial exploration of LLM-based CDSS capabilities. (3) Only 5 LLMs in complex clinical decision-making tasks were evaluated. (4) Bias and fairness of output were not evaluated. Studies have shown that LLM outputs may encode gender and racial biases. (5) In this study, we only tested LLM performance based on one prompt. As different prompt strategies may influence model outputs, future research should focus on designing more precise prompts. (6) Rapid development of new LLM models and versions limits conclusions from study results. (7) Knowledge base data were limited, such as the use of only local institutional monographs and drug use guidelines due to copyright constraints, which may affect generalizability.

Future research should consider several key directions to enhance the accuracy and reliability of LLM-based CDSSs. First, improvements to current tested pipeline are needed, particularly through iterative refinement of information retrieval strategies. This includes chunking and indexing methods, as well as exploration of alternative knowledge sources like real-time online browsing or integration with AI agents. Second, research should focus on optimizing human-computer interaction and clinician-AI collaboration. Although our findings indicate better performance when the model is used collaboratively with clinical pharmacists, critical issues such as model bias, automation bias, and the erosion of trust in AI systems require further evaluation, particularly when deployed at scale in real-world settings. Finally, investigating newly released models with advanced reasoning capabilities, such as OpenAI's o3-mini and DeepSeek's R1, offers promising potential. These models support chain-of-thought reasoning, which can enhance transparency by revealing the steps behind an output, thereby addressing concerns around the "black box" nature of many AI systems and improving user trust.

The integration of LLM-based CDSSs presents as a potential tool in improving prescription safety. Our study reveals that, when used in tandem with pharmacists, identification of DRPs is enhanced, surpassing the accuracy of humans alone. The combined pharmacist and LLM model (co-pilot mode) demonstrates superior performance in detecting severe DRPs and offers a promising hybrid solution for improving patient safety in medication management. This co-pilot model could represent the next step in CDSS development, merging human expertise with the analytical prowess of AI to improve healthcare outcomes.

Limitations of the study

This study's limitations constrain its immediate clinical applicability. It addressed only five DRP categories using a small set of complex, fictional scenarios that may not reflect the diversity of real-world cases. Test cases were drawn from known pharmacist-reported errors, introducing potential bias. The evaluation of five LLMs with a single prompt, without assessing output bias or fairness, further limits generalizability. Additionally, rapid LLM evolution and reliance on local drug references restrict applicability across broader clinical settings. These factors underscore

the need for further validation before integrating such tools into routine clinical practice.

RESOURCE AVAILABILITY

Lead contact

Further information and requests for resources should be directed to and will be fulfilled by the lead contact, Dr. Daniel Shu Wei Ting (daniel.ting.s.w@singhealth.com.sg).

Materials availability

This study did not generate new unique reagents.

Data and code availability

- Individual participant data will be made available on reasonable request, such as for research collaboration, directed to the corresponding author (also the lead contact), Dr. Daniel Shu Wei Ting.
- Sample dataset and codes are openly available at https://github.com/Liyuan1Y/CDSS-LLM-Cookbook/tree/main.
- Any additional information required to reanalyze the data reported in this work paper is available from the lead contact upon request.

ACKNOWLEDGMENTS

This work was supported by the National Medical Research Council, Singapore (grants MOH-001689-00, MOH-000655-00, and MOH-001014-00); Duke-NUS Medical School (grants Duke-NUS/RSF/2021/0018, 05/FY2020/EX/15-A58, and 05/FY2022/EX/66-A128); and Agency for Science, Technology and Research, Singapore (grants A20H4g2141 and H20C6a0032).

AUTHOR CONTRIBUTIONS

J.C.L.O., L.J., K.E., and D.S.W.T. developed the initial concept, design of the study, and the initial manuscript draft. R.J.Z., C.M.Y.K., K.Z.H.L., and X.C. refined the study design and analyzed results. G.Y.S.L., D.Y.Z.L., G.G.R.S., Y.H.K., J.Y.M.T., J.K.C., A.T., K.J.G., C.P.L., T.M.N., and N.L. refined and vetted the manuscript.

DECLARATION OF INTERESTS

The authors declare no competing interests.

STAR★METHODS

Detailed methods are provided in the online version of this paper and include the following:

- KEY RESOURCES TABLE
- EXPERIMENTAL MODEL AND STUDY PARTICIPANT DETAILS
  - Development of prescribing error scenarios
  - Development of reference standard
  - Development and validation of LLM tool
  - Knowledge source
  - LLM prompt
  - Generation of LLM and Pharmacist Responses
  - Assessment of accuracy of responses
- METHOD DETAILS
- QUANTIFICATION AND STATISTICAL ANALYSIS

SUPPLEMENTAL INFORMATION

Supplemental information can be found online at https://doi.org/10.1016/j.xcrm.2025.102323.

Received: November 21, 2024
Revised: April 30, 2025
Accepted: August 4, 2025
Published: September 24, 2025

REFERENCES

1. Foot, H., Scott, I., Sturman, N., Whitty, J.A., Rixon, K., Connelly, L., Williams, I., and Freeman, C. (2022). Impact of pharmacist and physician collaborations in primary care on reducing readmission to hospital: A systematic review and meta-analysis. Res. Social Adm. Pharm. *18*, 2922–2943. https://doi.org/10.1016/j.sapharm.2021.07.015.
2. Al Hamid, A., Ghaleb, M., Aljadhey, H., and Aslanpour, Z. (2014). A systematic review of hospitalization resulting from medicine-related problems in adult patients. Br. J. Clin. Pharmacol. *78*, 202–217. https://doi.org/10.1111/bcp.12293.
3. Walsh, E.K., Hansen, C.R., Sahm, L.J., Kearney, P.M., Doherty, E., and Bradley, C.P. (2017). Economic impact of medication error: a systematic review. Pharmacoepidemiol. Drug Saf. *26*, 481–497. https://doi.org/10.1002/pds.4188.
4. Elliott, R.A., Camacho, E., Jankovic, D., Sculpher, M.J., and Faria, R. (2021). Economic analysis of the prevalence and clinical and economic burden of medication error in England. BMJ Qual. Saf. *30*, 96–105. https://doi.org/10.1136/bmjqs-2019-010206.
5. Velo, G.P., and Minuz, P. (2009). Medication errors: prescribing faults and prescription errors. Br. J. Clin. Pharmacol. *67*, 624–628. https://doi.org/10.1111/j.1365-2125.2009.03425.x.
6. Osheroff, J.A., Teich, J.M., Levick, D., Saldana, L., Velasco, F., Sittig, D.F., Rogers, K., and Jenders, R. (2023). Improving Outcomes with Clinical Decision Support: An Implementer's Guide, Second Edition (HIMSS).
7. Sutton, R.T., Pincock, D., Baumgart, D.C., Sadowski, D.C., Fedorak, R.N., and Kroeker, K.I. (2020). An overview of clinical decision support systems: benefits, risks, and strategies for success. ReviewPaper. NPJ Digital Med. *3*, 17. https://doi.org/10.1038/s41746-020-0221-y.
8. Helmons, P.J., Suijkerbuijk, B.O., Nannan Panday, P.V., and Kosterink, J.G. (2015). Drug-drug interaction checking assisted by clinical decision support: a return on investment analysis. J. Am. Med. Inform. Assoc. *22*, 764–772. https://doi.org/10.1093/jamia/ocu010.
9. Taegtmeyer, A.B., Kullak-Ublick, G.A., Widmer, N., Falk, V., and Jetter, A. (2023). Clinical Usefulness of Electronic Drug-Drug Interaction Checking in the Care of Cardiovascular Surgery Inpatients. Cardiology *123*, 219–222. https://doi.org/10.1159/000343272.
10. Khalifa, M., and Zabani, I. (2016). Improving Utilization of Clinical Decision Support Systems by Reducing Alert Fatigue: Strategies and Recommendations. Stud. Health Technol. Inform. *226*, 51–54.
11. Ancker, J.S., Edwards, A., Nosal, S., Hauser, D., Mauer, E., and Kaushal, R.; with the HITEC Investigators (2017). Effects of workload, work complexity, and repeated alerts on alert fatigue in a clinical decision support system. BMC Med. Inform. Decis. Mak. *17*, 36. https://doi.org/10.1186/s12911-017-0430-8.
12. Olakotan, O.O., and Yusof, M.M. (2021). The appropriateness of clinical decision support systems alerts in supporting clinical workflows: A systematic review. Health Informatics J. *27*, 14604582211007536. https://doi.org/10.1177/14604582211007536.
13. Thirunavukarasu, A.J., Ting, D.S.J., Elangovan, K., Gutierrez, L., Tan, T.F., and Ting, D.S.W. (2023). Large language models in medicine. Nat. Med. *29*, 1930–1940. https://doi.org/10.1038/s41591-023-02448-8.
14. Jiang, L.Y., Liu, X.C., Nejatian, N.P., Nasir-Moin, M., Wang, D., Abidin, A., Eaton, K., Riina, H.A., Laufer, I., Punjabi, P., et al. (2023). Health system-scale language models are all-purpose prediction engines. Nature *619*, 357–362. https://doi.org/10.1038/s41586-023-06160-y.

15. Shoja, M.M., Van de Ridder, J.M.M., and Rajput, V. (2023). The Emerging Role of Generative Artificial Intelligence in Medical Education, Research, and Practice. Cureus *15*, e40883. https://doi.org/10.7759/cureus.40883.

16. Kumar, Y., Koul, A., Singla, R., and Ijaz, M.F. (2023). Artificial intelligence in disease diagnosis: a systematic literature review, synthesizing framework and future research agenda. J. Ambient Intell. Humaniz. Comput. *14*, 8459–8486. https://doi.org/10.1007/s12652-021-03612-z.

17. Caranfa, J.T., Bommakanti, N.K., Young, B.K., and Zhao, P.Y. (2023). Accuracy of Vitreoretinal Disease Information From an Artificial Intelligence Chatbot. JAMA Ophthalmol. *141*, 906–907. https://doi.org/10.1001/jamaophthalmol.2023.3314.

18. Suharwardy, S., Ramachandran, M., Leonard, S.A., Gunaseelan, A., Lyell, D.J., Darcy, A., Robinson, A., and Judy, A. (2023). Feasibility and impact of a mental health chatbot on postpartum mental health: a randomized controlled trial. AJOG Glob. Rep. *3*, 100165. https://doi.org/10.1016/j.xagr.2023.100165.

19. Johnston, K., O'Reilly, C.L., Scholz, B., Georgousopoulou, E.N., and Mitchell, I. (2021). Burnout and the challenges facing pharmacists during COVID-19: results of a national survey. Int. J. Clin. Pharm. *43*, 716–725. https://doi.org/10.1007/s11096-021-01268-5.

20. Jones, G.M., Roe, N.A., Louden, L., and Tubbs, C.R. (2017). Factors Associated With Burnout Among US Hospital Clinical Pharmacy Practitioners: Results of a Nationwide Pilot Survey. Hosp. Pharm. *52*, 742–751. https://doi.org/10.1177/0018578717732339.

21. Macaron, M.M., Segun-Omosehin, O.A., Matar, R.H., Beran, A., Nakanishi, H., Than, C.A., and Abulseoud, O.A. (2023). A systematic review and meta analysis on burnout in physicians during the COVID-19 pandemic: A hidden healthcare crisis. Front. Psychiatry *13*, 1071397. https://doi.org/10.3389/fpsyt.2022.1071397.

22. Krusche, M., Callhoff, J., Knitza, J., and Ruffer, N. (2024). Diagnostic accuracy of a large language model in rheumatology: comparison of physician and ChatGPT-4. Rheumatol. Int. *44*, 303–306. https://doi.org/10.1007/s00296-023-05464-6.

23. Wang, H., Gao, C., Dantona, C., Hull, B., and Sun, J. (2024). DRG-LLaMA: tuning LLaMA model to predict diagnosis-related group for hospitalized patients. npj Digit. Med. *7*, 16. https://doi.org/10.1038/s41746-023-00989-3.

24. Wang, A.Y., Lin, S., Tran, C., Homer, R.J., Wilsdon, D., Walsh, J.C., Goebel, E.A., Sansano, I., Sonawane, S., Cockenpot, V., et al. (2024). Assessment of Pathology Domain-Specific Knowledge of ChatGPT and Comparison to Human Performance. Arch. Pathol. Lab Med. *148*, 1152–1158. https://doi.org/10.5858/arpa.2023-0296-OA.

25. Li, J., Guan, Z., Wang, J., Cheung, C.Y., Zheng, Y., Lim, L.L., Lim, C.C., Ruamviboonsuk, P., Raman, R., Corsino, L., et al. (2024). Integrated image-based deep learning and language models for primary diabetes care. OriginalPaper. Nat. Med. *30*, 2886–2896. https://doi.org/10.1038/s41591-024-03139-8.

26. Pais, C., Liu, J., Voigt, R., Gupta, V., Wade, E., and Bayati, M. (2024). Large language models for preventing medication direction errors in online pharmacies. Nat. Med. *30*, 1574–1582. https://doi.org/10.1038/s41591-024-02933-8.

27. Van Veen, D., Van Uden, C., Blankemeier, L., Delbrouck, J.B., Aali, A., Bluethgen, C., Pareek, A., Polacin, M., Reis, E.P., Seehofnerová, A., et al. (2024). Adapted large language models can outperform medical experts in clinical text summarization. Nat. Med. *30*, 1134–1142. https://doi.org/10.1038/s41591-024-02855-5.

28. Sheikh, M.S., Dreesman, B., Barreto, E.F., Thongprayoon, C., Miao, J., Suppadungsuk, S., Mao, M.A., Qureshi, F., Pham, J.H., Craici, I.M., et al. (2024). Identification of kidney-related medications using AI from self-captured pill images. Ren. Fail. *46*, 2402075.

29. Zaretsky, J., Kim, J.M., Baskharoun, S., Zhao, Y., Austrian, J., Aphinyanaphongs, Y., Gupta, R., Blecker, S.B., and Feldman, J. (2024). Generative Artificial Intelligence to Transform Inpatient Discharge Summaries to Patient-Friendly Language and Format. JAMA Netw. Open *7*, e240357.

30. Flory, J.H., Ancker, J.S., Kim, S.Y.H., Kuperman, G., Petrov, A., and Vickers, A. (2025). Large Language Model GPT-4 Compared to Endocrinologist Responses on Initial Choice of Glucose-Lowering Medication Under Conditions of Clinical Uncertainty. Diabetes Care *48*, 185–192.

31. Van Spall, H.G., Wallentin, L., Yusuf, S., Eikelboom, J.W., Nieuwlaat, R., Yang, S., Kabali, C., Reilly, P.A., Ezekowitz, M.D., and Connolly, S.J. (2012). Variation in warfarin dose adjustment practice is responsible for differences in the quality of anticoagulation control between centers and countries: an analysis of patients receiving warfarin in the randomized evaluation of long-term anticoagulation therapy (RE-LY) trial. Circulation *126*, 2309–2316. https://doi.org/10.1161/CIRCULATIONAHA.112.101808.

32. Metsvaht, T., Nellis, G., Varendi, H., Nunn, A.J., Graham, S., Rieutord, A., Storme, T., McElnay, J., Mulla, H., Turner, M.A., and Lutsar, I. (2015). High variability in the dosing of commonly used antibiotics revealed by a Europe-wide point prevalence study: implications for research and dissemination. BMC Pediatr. *15*, 41. https://doi.org/10.1186/s12887-015-0359-y.

33. Barnett, S., Kurniawan, S., Thudumu, S., Brannelly, Z., and Abdelrazek, M. (2024). Seven Failure Points When Engineering a Retrieval Augmented Generation System. Preprint at arXiv. https://doi.org/10.48550/arXiv.2401.05856.

34. Yu, F., Moehring, A., Banerjee, O., Salz, T., Agarwal, N., and Rajpurkar, P. (2024). Heterogeneity and predictors of the effects of AI assistance on radiologists. Nat. Med. *30*, 837–849. https://doi.org/10.1038/s41591-024-02850-w.

35. Tikhomirov, L., Semmler, C., McCradden, M., Searston, R., Ghassemi, M., and Oakden-Rayner, L. (2024). Medical artificial intelligence for clinicians: the lost cognitive perspective. Lancet Digit. Health *6*, e589–e594. https://doi.org/10.1016/S2589-7500(24)00095-5.

36. Kwan, J.L., Lo, L., Ferguson, J., Goldberg, H., Diaz-Martinez, J.P., Tomlinson, G., Grimshaw, J.M., and Shojania, K.G. (2020). Computerised clinical decision support systems and absolute improvements in care: meta-analysis of controlled clinical trials. BMJ *370*, m3216. https://doi.org/10.1136/bmj.m3216.

37. Corny, J., Rajkumar, A., Martin, O., Dode, X., Lajonchère, J.P., Billuart, O., Bézie, Y., and Buronfosse, A. (2024). A machine learning–based clinical decision support system to identify prescriptions with a high risk of medication error. J. Am. Med. Inform. Assoc. *27*, 1688–1694. https://doi.org/10.1093/jamia/ocaa154.

38. Rozenblum, R., Rodriguez-Monguio, R., Volk, L.A., Forsythe, K.J., Myers, S., McGurrin, M., Williams, D.H., Bates, D.W., Schiff, G., and Seoane-Vazquez, E. (2020). Using a Machine Learning System to Identify and Prevent Medication Prescribing Errors: A Clinical and Cost Analysis Evaluation. Jt. Comm. J. Qual. Patient Saf. *46*, 3–10. https://doi.org/10.1016/j.jcjq.2019.09.008.

39. Li, B., Meng, T., Shi, X., Zhai, J., and Ruan, T. (2023). MedDM:LLM-executable clinical guidance tree for clinical decision-making. Preprint at arXiv. https://doi.org/10.48550/arXiv.2312.02441.

40. Rao, A., Kim, J., Kamineni, M., Pang, M., Lie, W., and Succi, M.D. (2023). Evaluating ChatGPT as an Adjunct for Radiologic Decision-Making. Preprint at medRxiv. https://doi.org/10.1101/2023.02.02.23285399.

41. Hines, L.E., Saverno, K.R., Warholak, T.L., Taylor, A., Grizzle, A.J., Murphy, J.E., and Malone, D.C. (2011). Pharmacists' awareness of clinical decision support in pharmacy information systems: an exploratory evaluation. Res. Social Adm. Pharm. *7*, 359–368. https://doi.org/10.1016/j.sapharm.2010.10.007.

42. Pharmaceutical Care Network Europe (PCNE). 2018. The PCNE classification V8.02. http://www.pcne.org/upload/files/230_PCNE_classification_V8-02.pdf.

43. American Journal of Hospital Pharmacy (1993). ASHP statement on pharmaceutical care. Am. J. Hosp. Pharm. *3*, 1720–1723.

44. Gates, P.J., Baysari, M.T., Mumford, V., Raban, M.Z., and Westbrook, J.I. (2019). Standardising the Classification of Harm Associated with Medication Errors: The Harm Associated with Medication Error Classification (HAMEC). Drug Saf. *42*, 931–939. https://doi.org/10.1007/s40264-019-00823-4.

45. Lu, Y., Green, A.R., Quiles, R., and Taylor, C.O. (2024). An Automated Strategy to Calculate Medication Regimen Complexity. AMIA Annu. Symp. Proc. *2023*, 1077–1086.

46. Nori, H., Lee, Y.T., Zhang, S., Carignan, D., Edgar, R., Fusi, N., King, N., Larson, J., Li, Y., Liu, W., et al. (2023). Can Generalist Foundation Models Outcompete Special-Purpose Tuning? Case Study in Medicine. Preprint at arXiv. https://doi.org/10.48550/arXiv.2311.16452.

CellPress
OPEN ACCESS

STAR★METHODS

KEY RESOURCES TABLE

REAGENT or RESOURCE	SOURCE	IDENTIFIER
Software and algorithms		
Gemini Flash, Gemini 1.5 Pro, GPT-4 Turbo, GPT-4 omni, Claude 3.5 Sonnet	Google, OpenAI, Anthropic	Sample dataset and code can be accessed at: https://github.com/Liyuan1Y/CDSS-LLM-Cookbook/tree/main. Any additional information required to reanalyse the data reported in this work paper is available from the lead contact A/Prof Daniel Ting upon request at daniel.ting.s.w@singhealth.com.sg

EXPERIMENTAL MODEL AND STUDY PARTICIPANT DETAILS

This was a prospective, cross-over, open-label study. The institutional ethics review board exempted this study from review as no identifiable patient data was used. An overview of the evaluation workflow is shown in Figure 5. Participants were registered pharmacists, practicing in 2 different tertiary acute care institutions. Participants were randomized using a random number generator into one of 2 arms. In the first arm, participants reviewed a set of patient case vignettes without LLM-CDS assistance in round 1. Participants waited at least one day before they are asked to review another set of patient case vignettes with appended LLM-CDS output in round 2. In the second arm, participants reviewed a set of case vignettes with LLM-CDS assistance in round 1, followed a set of vignettes without LLM-CDS assistance after the washout period. During each round, participants were asked to complete the reviews of 7–8 case within a time frame of 1.5 h. Rounds consisted of a fresh set of 7–8 vignettes (not used in the previous round). We provide details of response generation in subsection on "Generation of LLM and Pharmacist Responses".

Development of prescribing error scenarios

A total of 91 different simulated prescribing error scenarios based on 40 case vignettes modeled after complex clinical cases were curated. Prescribing error scenarios were adapted from our institution's pharmacy intervention and error reporting databases to maintain realism. The vignettes covered clinical scenarios from 16 different medical or surgical subspecialties (Cardiology, Colorectal Surgery, Endocrinology, Emergency Medicine, Family Medicine, Gastroenterology, General Surgery, General Medicine, Haematology, Infectious Disease, Neurology, Oncology, Ophthalmology, Respiratory Medicine, Urology, Vascular Surgery) with some cases involving more than one discipline. Each case vignette consisted of a patient clinical note and medication prescription. Prescribing errors in the clinical scenarios were designed to be reflective of drug-related problems (DRP) encountered in an acute care tertiary medical institution in Singapore. We present a sample of one case vignette in Figure S1. A detailed description of all case vignettes is found in Data S1. The Anatomical Therapeutic Chemical (ATC) category of medications prescribed in each clinical scenario is also presented in Table S2.

Development of reference standard

The reference standard was developed through manual grading of DRP categories and severity by a multi-disciplinary expert panel. The expert panel consisted of pharmacotherapy board certified pharmacists and physicians with >10 years of clinical practice experience in tertiary hospitals. Every clinical scenario was graded by at least 1 pharmacist and 1 physician member of the panel. Any disagreements were resolved by a 3rd independent member. DRPs from each clinical scenario were categorized using the Pharmaceutical Care Network Europe (PCNE) classification V9.1 and ASHP statement of pharmaceutical care as a guide.[42,43] Types of errors in the clinical scenarios included adverse drug reaction, allergy, drug-drug interactions, duplication of therapy, inappropriate choice of therapy, inappropriate dosage regimen, no indication and omission of drug therapy. The potential severity of these errors were graded according to the Harm Associated with Medication Error Classification (HAMEC) classification tool.[44] In addition, we calculated the MCRI (medication regimen complexity index) for each case. The MCRI is a 65-item instrument validated across different study populations to quantify medication complexity at patient level and identify patients who require medication therapy management interventions. This instrument demonstrated moderate to good concordance between patient-level MRCI scores and experts in ranking of medication regimen complexity.[45]

Development and validation of LLM tool

We first evaluated the performance of 5 different state-of-the-art LLMs (Gemini Flash, Gemini 1.5 Pro, GPT-4 Turbo, GPT-4 omni, Claude 3.5 Sonnet) with and without provision of contextual knowledge. We then developed and validated a retrieval augmented generation (RAG) based-LLM model through optimizing the transformation of specialized documents into embedding vectors. This process utilizes advanced pre-processing and embedding models, with a focus on similarity-based retrieval between query vectors and embedded vectors of targeted documents, including drug monographs and hospital drug-use protocols.

Our RAG framework integrated the Llamaindex, relying on auto-merging retrieval to provide contextualized search. We optimized pharmacological knowledge corpus with manual indexing of drug names improved specificity during retrieval. Total number of retrieved passages were set as 50, expanded with increased context size in latest LLMs, for optimal breadth and depth of information retrieval.

The prompt was designed as a series of tasks, simulating human clinical thinking process. Each task required retrieval of knowledge for each medication on the prescription (e.g., Is this medication indicated for the patient?). The response from each task is collated into a final LLM model for clinical synthesis and recommendations. A diagrammatic view of the sequence is shown in Figure S2. All responses were generated and analyzed in triplicates to account for reproducibility.

Knowledge source

Institutional medication use and dosing guidelines, medication monographs were used as sources of information. Each medication monograph was split into 4 separate sections according to the following categories of information: (1) Adverse drug reactions, cautions and contraindications, (2) ATC category and mechanism of action, (3) Drug-drug interactions, (4) Drug dosing and adjustments.

LLM prompt

We designed a prompt strategy informed by prior research and expert recommendations. Studies have demonstrated that prompting methods such as zero shot, few shot, and chain of thought can significantly influence LLM performance in medical applications, with no single approach universally optimal across all models and tasks. Based on this, we followed best practice guidelines and adopted a chain-of-thought prompting format. This approach was chosen to reflect the structured, step by step reasoning processes commonly used by clinicians and pharmacists, thereby enabling a more clinically relevant assessment of the LLM's performance in evaluating medication appropriateness.[46] The final adapted prompt used is presented in Figure S3. To standardize LLM outputs, we prompted the models to present its final recommendations in a SOAP (Subjective, Objective, Assessment and Plan) format.

Generation of LLM and Pharmacist Responses

Native LLM and RAG-LLM were presented with all case scenarios to generate a response. For reproducibility, we generated all LLM and RAG-LLM outputs in triplicates, resulting in a total of 1,200 responses for assessment. Respective performance was used to select the best model to be used as comparator against human only and for adoption in co-pilot mode.

We randomly assigned the scenarios to participants to independently identify any DRPs and generate a standard clinical note in standardized healthcare SBAR (situation, background, assessment, recommendation) format for each scenario. Each pharmacist had access to all institutional protocols and guidelines.

Assessment of accuracy of responses

We evaluated the accuracy of all DRP responses using the human expert panel as the criterion standard (Table S1). To determine whether the LLMs accurately identified these issues, we employed a structured methodology:

(1) Evaluation was grounded upon internationally accepted definition of a drug-related problem, specifically the one provided by the Pharmaceutical Care Network Europe (PCNE), version 9.1. According to this definition, ‘‘A Drug-Related Problem is an event or circumstance involving drug therapy that actually or potentially interferes with desired health outcomes.’’
(2) LLM output should identify the medication order(s) that posed potential risk(s) to the patient.
(3) Third, LLM should propose an appropriate action or intervention. Recommended actions were evaluated using a rubric of acceptable actions, adapted from the PCNE classification system for DRPs (Table S2).
(4) Finally, an overall assessment (global assessment) of the LLM output for the presence of any clinically significant risk to the patient.

For a response to be graded as accurate, the DRP response should fulfill criteria (1) – (3), and assessed to present no clinically significant risk to patient as described in (4).

METHOD DETAILS

This prospective, crossover, open-label study assessed the effectiveness of a large language model (LLM)-based clinical decision support system (CDSS) in identifying prescribing errors. Pharmacists from two tertiary hospitals reviewed 91 prescribing error scenarios across 40 complex clinical vignettes, drawn from 16 medical specialties. Each participant completed reviews in two randomized arms: with and without LLM-CDSS assistance, separated by a washout period. A multi-disciplinary expert panel annotated each case using the Pharmaceutical Care Network Europe (PCNE) v9.1 and HAMEC frameworks to classify drug-related problems (DRPs) and assess potential harm severity.

Five state-of-the-art LLMs (Gemini Flash, Gemini 1.5 Pro, GPT-4 Turbo, GPT-4 omni, Claude 3.5 Sonnet) were tested, both in their native forms and within a retrieval-augmented generation (RAG) framework. The RAG system incorporated LlamaIndex with auto-merging retrieval and indexed pharmacological knowledge (e.g., drug monographs, dosing protocols). Prompts followed a

CellPress
OPEN ACCESS

chain-of-thought structure and were standardized to a SOAP format. Responses were generated in triplicate for each case to assess reproducibility. Pharmacist responses were produced in SBAR format. Claude 3.5 Sonnet, the top-performing model, was selected for the co-pilot arm.

Accuracy was determined by comparing LLM and pharmacist outputs to expert consensus. A response was marked correct if it identified the relevant medication and DRP, recommended an appropriate intervention, and posed no risk of harm. Quantitative metrics included accuracy, precision, recall, and F1 score, calculated using R version 4.3.0. Statistical analysis included one-way ANOVA and Pearson correlation with the Medication Regimen Complexity Index (MCRI). To evaluate consistency across LLM responses, BERT scores, BLEU scores, and cosine similarity were computed, indicating high reproducibility of model outputs.

QUANTIFICATION AND STATISTICAL ANALYSIS

Concordance between performance of co-pilot and autonomous modes, using human expert input as the benchmark, was measured in terms of accuracy, precision, recall, and F1 score. Accuracy is expressed as a percentage of correctly identified DRP against expert. Precision, which denotes the fraction of DRPs correctly identified amongst all suggested DRPs, was defined as precision = true positives/(true positives + false positives). Recall, or the fraction of all DRPs in the criterion standard correctly identified by LLMs, was defined as recall = true positives/(true positives + false negatives). The F1 score is the harmonic mean of precision and recall, and thus penalizes unbalanced precision and recall scores (i.e., is higher when both precision and recall have similar values): F1 score = (2 $\times$ precision $\times$ recall)/(precision + recall). The higher any of the 3 scores, the better the response, with 1 being the maximum value for each score. One-way ANOVA was applied to compare differences in accuracy between different modes with a one-tailed $\alpha = 0.05$. Pearson's correlation analysis was performed to examine correlation between MCRI and accuracy. Data analysis, calculation of precision, recall and F1 scores were done in R version 4.3.0 (R Project for Statistical Computing).

BERT scores, Cosine Similarity and BLEU scores were generated to assess semantic similarity across repeated LLM responses. These metrics were chosen to assess both semantic consistency and lexical overlap between responses. All LLM responses were generated via consecutive API calls in a single session. To quantify overall reproducibility of LLM responses, the sum and average of each metric across the three comparisons were calculated. This provides a structured assessment of LLM consistency across multiple response iterations.

Cell Reports Medicine, Volume *6*

Supplemental information

Large language model as clinical decision support system augments medication safety in 16 clinical specialties

Jasmine Chiat Ling Ong, Liyuan Jin, Kabilan Elangovan, Gilbert Yong San Lim, Daniel Yan Zheng Lim, Gerald Gui Ren Sng, Yu He Ke, Joshua Yi Min Tung, Ryan Jian Zhong, Christopher Ming Yao Koh, Keane Zhi Hao Lee, Xiang Chen, Jack Kian Ch'ng, Aung Than, Ken Junyang Goh, Chuan Poh Lim, Tat Ming Ng, Nan Liu, and Daniel Shu Wei Ting

Table of Supplementary Data

Title	Legend
Figure S1: Sample of Case Vignette.	Abbreviations (CVM: cardiovascular Medicine, BP: blood pressure, RR: respiratory rate, Ht: height, BMI: body mass index, T: temperature, Hb: hemoglobin, TW: total white count, Plt: platelet count, SCr: serum creatinine, INR: international normalized ratio, PMHx: past medical history, DM: diabetes mellitus, HTN: hypertension, HLD: hyperlipidemia, CKD: chronic kidney disease, BKA: below knee amputation , OM: osteomyelitis, I&D: incision and drainage, DVT: deep vein thrombosis, PE: pulmonary embolism, VAS: vascular, HOPC: history of presenting complain, CABG: coronary artery bypass grafting, NSAID (non-steroidal anti-inflammatory drug), LV: left ventricle, RV: right ventricle, EF: ejection fraction, MI: myocardial infarction, O/E: on examination, NSR: normal sinus rhythm, JVP: jugular venous pressure, SNT: soft non tender, BS: bowel sound, LAD: left anterior descending artery, AV: atrioventricular, LCx: left circumflex artery, ICA: intermediate care area, DAPT: dual anti-platelet therapy)
Figure S2: Overview of RAG-LLM architecture with auto-merging retrieval	NA
Figure S3: Final adapted prompt	NA
Table S1: Rubric to evaluate acceptable actions produced by LLM based on DRP category	NA
Table S2: DRPs and Risk / Potential for Harm Categories	NA
Table S3: Results from reasoning model (OpenAI's o4-mini)	SD: Standard Deviation
Data S1: Summary of Case Vignettes	NA

Figure S1: Sample of Case Vignette.

CVM Inpatient Daily Ward Round
General Information:
Admission Date 11-Sep-2024 14:24:49 Post Admission Day 2.

Clinical Notes:
Latest Vital Signs: 12-Sep-2024 07:00:00
12/09/2024 07:00:00
Pain Score: 0
BP (NIBP) (mmHg): 103/83 (101-144/68-118), **HR (beats/min):** 65 (55-99)
RR (breaths/min): 12 (12-21), **SPO2 (%):** 100 (95-100), **O2 Therapy (L/min):** NP 2 (2-3)
Hypocount (from 11/09/2024 06:00:00 to 12/09/2024 07:18:37):
15.8(H) <- 20.4(H) <- 27.5(HH) <- 21.0(H) <- 5.9(N)
Ht: 182 cm (11-Sep-2024 15:18:00), **Wt:** 99.1 kg (11-Sep-2024 16:57:00)
BMI: 29.9, **BSA:** 2.24 m2
12/09/2024 04:00:00
T (deg.C): 36.6, **Tmax (deg.C):** 36.6 (12/09/2024 04:00:00)

Latest I/O from 11/09/2024 06:01 to 12/09/2024 06:00
Intake: 2100 Output: 3750 Net: -1650
Intake: 2100
- Diet Fluid Volume: 100
- IV: 2000
Output: 3750
- Urine Output: 3750

Lab Values:
12/09/2024 06:15
Hb: **11.3 [12.0 – 16.0 G/DL]**
TW: 8.64 [4.0 – 10.0 x 10(9)/L]
Plt: 250 [140 – 440 x 10(9)/L]
SCr: 100 [37 – 75 UMOL/L]
INR: 1.2

Surgical operations:
right LL deep vein thrombolysis and creation of SFA-LSV arteriovenous fistual on 15-May-2024.

Clinical Notes:
61 / M / Malay
Allergic to mefenamic acid and salicylate, claims rashes and facial swelling
Lives with wife and children
ADL independent, Community ambulant

PMHx:
1. Poorly controlled DM
2. HTN, HLD
3. CKD Stage 2
4. Necrotising fasciitis of R LL
- s/p R BKA 12/2/20
5. Left foot OM
- s/p 2nd and 3rd ray amputation Dec 2019
6. Large right medial thigh abscess
- s/p I&D 6/3/24
7. Recurrent DVT and PE (on long-term warfarin) - f/u Haem and VAS
- 2018: L LL DVT
- 2020: Extensive left arm DVT s/p R LL thrombolysis and IVC filter insertion
- s/p R LL venous thrombectomy with stenting 28/4/24
- Possible right interlobar pulmonary embolism (April 2024)

HOPC:
Chest pain since Friday
Started at rest
Central pressing with dyspnoea and diaphoresis
No radiation
Stuttering since then
Worse with exertion
Worse since 5am today hence presented
Still has ongoing mild discomfort
No fever or intercurrent illness
No bleeding history
Normally takes warfarin at night - has not taken for today
All his brothers had CABG at his age
Single NSAID (mefenamic acid) allergy - rashes
Progress in emergency department:
Bedside echo: LV impaired systolic function (also does look dilated), RV normal size and function, EF < 45%
ECG: evolved anterior MI (anterior Q waves new since May 2024)
Cath lab activated

O/E:
Vitals stable, afebrile
SpO2 95-100% on 2LNP
Poor H/C control
Telemetry ON: NSR
JVP not elevated
H S1 S2 NIL murmurs
L Clear
Abdomen SNT, BS+

Issues and progress:
1) Evolved anterior MI with ongoing chest discomfort
- Cath activated
- Findings:
- Co-dominant coronary system
- Severe proximal to mid LAD stenosis, involving the bifurcation with the dominant diagonal branch
- Diffusely diseased AV groove branch (continuation of the distal LCx)
- Anomalous origin of the RCA (high anterior). Diffuse moderate proximal to mid RCA narrowing.
- Successful PCI to the LAD and implantation of overlapping drug eluting stent (Onyx) from mid to proximal LAD. Ostium of the diagonal branch preserved with modified jailed balloon technique.
- Post cath stable - to ICA
2) b/g of recurrent VTE on Warfain
Hold off warfarin for now
Bridge with clexane
Trend Hb whilst on DAPT + anticoagulation
- Hb on 12/9 11.3 (stable)

Plan:
To GW with telemetry
Vitals as per ward protocol
CBG TDS + 10PM with SCSI cover
Low salt/low fat/DM diet
Heart failure for medical therapy in the interim
Aspirin for 1/12
Clopidogrel for at least 12 months

Allergies:
Mefenamic Acid. Facial Swelling.
Salicylate. Facial Swelling

Medications Prescribed	Status
Sodium Chloride 0.9% InFUSion, IV Intermittent 2,000 mL, Once, Infuse Over 16 hour, 125 mL/hr	Active
Enoxaparin Sodium Injection, Sub-Cutaneous 60 mg, BD	Active
ACTRAPID [Insulin Soluble] Injection, Sub-Cutaneous 4 unit, Once	Active
LANTUS [Insulin Glargine] Solostar, Sub-Cutaneous 24 unit, OM	Active
NovoRAPID [Insulin Aspart] Flexpen, Sub-Cutaneous 8 unit, TDS (Pre-meal)	Active
Aspirin Tablet, PO 100 mg, OM	Active
Clopidogrel Tablet, PO 75 mg, OM	Active
OMEprazole Capsule, PO 20 mg, OM	Active
Glyceryl Trinitrate Tablet, Sub-Lingual 0.5 mg, Use as directed PRN Chest Pain	Active
Linagliptin Tablet, PO 5 mg, OM	Active
Bisoprolol Fumarate Tablet, PO 2.5 mg, OM	Active
Perindopril Erbumine [Tert-butylamine] Tablet, PO 2 mg, OM	Active
Neurobion Tablet [Vit B1 100mg, B6 200mg, B12 200mcg], PO 1 tablet, OM	Active

Abbreviations (CVM: cardiovascular Medicine, BP: blood pressure, RR: respiratory rate, Ht: height, BMI: body mass index, T: temperature, Hb: hemoglobin, TW: total white count, Plt: platelet count, SCr: serum creatinine, INR: international normalized ratio, PMHx: past medical history, DM: diabetes mellitus, HTN: hypertension, HLD: hyperlipidemia, CKD: chronic kidney disease, BKA: below knee amputation , OM: osteomyelitis, I&D: incision and drainage, DVT: deep vein thrombosis, PE: pulmonary embolism, VAS: vascular, HOPC: history of presenting complain, CABG: coronary artery bypass grafting, NSAID (non-steroidal anti-inflammatory drug), LV: left ventricle, RV: right ventricle, EF: ejection fraction, MI: myocardial infarction, O/E: on examination, NSR: normal sinus rhythm, JVP: jugular venous pressure, SNT: soft non tender, BS: bowel sound, LAD: left anterior descending artery, AV: atrioventricular, LCx: left circumflex artery, ICA: intermediate care area, DAPT: dual anti-platelet therapy)

Figure S2: Overview of RAG-LLM architecture with auto-merging retrieval

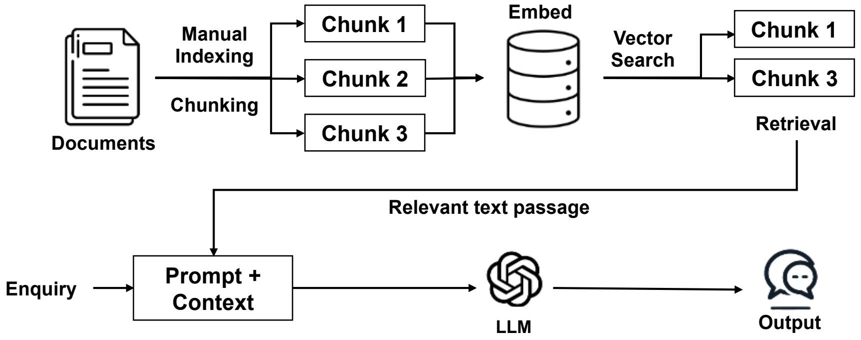

Figure S3: Final adapted prompt

For the Junior Pharmacist

Assume the role of a clinical pharmacist. You are tasked to perform a medication chart review for a patient admitted to the department of <cardiology>. I will provide you with the patient's medication list, clinical note, and drug monographs as reference. Identify drug related problems specific to the patient's profile using this guide:

- Medication Indications: Confirm that each medication has a clear indication and that current health conditions are being addressed with appropriate pharmacotherapy. [Drug monograph reference sections: "Pharmacologic Category", "Use: Labeled Indications", "Use: Off-Label: Adult", "Mechanism of Action"]
- Dosing Verification: Check that the dosages of medications are within the recommended ranges and adjust if necessary, considering factors such as age, kidney function, and liver function [Drug monograph reference sections: "Dosing: Adult", "Dosing: Older Adult", "Dosing: Altered Kidney Function: Adult", "Dosing: Hepatic Impairment: Adult"]
- Drug-Drug Interactions: Investigate potential interactions between current medications that could increase the risk of adverse effects or reduce therapeutic efficacy and warrants a change in therapy or monitoring tests. [Drug monograph reference sections: "Metabolism/Transport Effects", "Drug Interactions"]
- Potential adverse drug reaction, contraindications and cautions, medication allergy [Drug monograph reference sections: "Special alerts", "ALERT: U.S. Boxed Warning", "Warnings/Precautions", "Contraindications", "Adverse Reactions", "Adverse Reactions (Significant): Considerations"]
- Medication Omissions: Look for any conditions that are not being treated which should be, according to the patient's history and current clinical guidelines.
- Any duplication in medication class or therapy
- Patient-Specific Factors: Take into account patient-specific factors such as age, allergies, and preferences that may influence medication selection and management.

Create a pharmacist recommendation note to address any identified drug related problem(s) in the following format: "situation, background, assessment, recommendation". Your plan should be clear and justified with specific recommendations for any changes to the medication regimen, including discontinuations, dose adjustments, or additions.

Table S1: Rubric to evaluate acceptable actions produced by LLM based on DRP category

**Category**	**Description**	**Acceptable Action**
Drug Selection	The cause of the DRP can be related to the selection of the drug, e.g. No indication for drug, inappropriate selection of drug, untreated indication	Discontinue medication; Substitute medication; Initiate medication for untreated indication
Drug Form	The cause of the DRP is related to the selection of the drug formulation	Substitute with different formulation of medication; Substitute with different medication of same formulation; Suggest different route of administration
Dose Selection	The cause of the DRP can be related to the selection of the dosage schedule or dosing regimen	Amend dosing regimen: dose or frequency
Treatment duration	The cause of the DRP is related to the duration of treatment	Amend medication duration; Discontinue medication; Resume medication (unintended discontinuation)

Table S2: DRPs and Risk / Potential for Harm Categories

Case No	DRP(s) Category and Description	Severity / Potential for Harm
1	(1) Drug allergy: Background of NSAIDS allergy (rash) but prescribed with aspirin without any challenge or test dose	Moderate
	(2) Inappropriate dosage regimen: Enoxaparin dosed at 60mg BD in obese patient of 90kg, no bleeding.	Serious
	(3) Omission of therapy: Omission of statin therapy in patient presenting with myocardial infarction	Moderate
	(4) Adverse drug reaction: Borderline blood pressure but prescribed with perindopril and bisoprolol	Moderate
2	(1) Drug Drug Interaction: Significant Interaction between atorvastatin and clarithromycin	Moderate
	(2) Inappropriate dosage regimen: Colchicine dosed in MG instead of MCG	Serious
	(3) Adverse drug reaction: Bradycardia but prescribed with both bisoprolol and ticagrelor	Moderate
3	(1) Wrong indication: Wrong drug of clarithromycin instead of clindamycin for cellulitis in patient with penicillin allergy	Serious
	(2) Inappropriate dosage regimen: Wrong dose of enoxaparin (dosed 1mg/kg BD) in patient with renal failure	Moderate
	(3) Drug drug interaction: Significant drug interaction (contraindicated) between sildenafil and isosorbide mononitrate	Serious
4	Control Case	NA
5	(1) Adverse drug reaction: Patient presented with hypokalemia, on intensive furosemide therapy without electrolyte replacement	Serious
	(2) Adverse drug reaction: Patient presenting with acute pulmonary edema but continued on beta-blocker therapy	Moderate
	(3) No indication for medication: Patient on triple antithrombotic therapy with aspirin / clopidogrel / enoxaparin post myocardial infarction, also continued on dipyridamole (chronic medication for previous history of CVA)	Serious
6	(1) Duplication of therapy: Duplication between Simvastatin and Atorvastatin. Both belong to the same category of HMG-CoA reductase inhibitor	Minor
	(2) Inappropriate dosing frequency for atorvastatin. It is usually given once daily, but does not exceed max daily dose	Minor
	(3) Adverse Drug Reaction: Patient has allergy to amoxicillin (rash) but was prescribed with Co-amoxiclav which contains amoxicillin	Moderate
7	(4) Adverse Drug Reaction: Acute kidney injury with hyperkalemia but continued on enalapril	Moderate
	(5) Adverse drug reaction: Acute kidney injury but prescribed with NSAID	Moderate
	(6) Adverse drug reaction: Presented with hypoglycemia but continued on glipizide	Serious
8	(1) Inappropriate dosage regimen: Wrong weight-based dose of calcitonin prescribed for patient with hypercalcemia of malignancy	Serious
	(2) Adverse drug reaction: Colecalciferol not held off despite hypercalcemia	Moderate
	(3) No indication: Wrong drug of cinnarizine prescribed instead of cinacalcet for patient with b/g secondary hyperparathyroidism	Minor
9	(1) Duplication of therapy: Patient with poorly controlled diabetes on both ultra-short-acting insulin Aspart and short-acting insulin Actrapid.	Moderate
	(2) Omission of therapy: Omisson of basal insulin such as insulatard in patient with poorly controlled diabetes	Moderate
	(3) Omission of therapy: Antibiotics not ordered for patient with suspected sepsis	Serious
10	(1) Adverse drug reaction: Laboratory results demonstrating non-anion gap metabolic acidosis with respiratory compensation, continued on acetazolamide	Moderate
	(2) Adverse drug reaction: Hydroxyzine (first generation antihistamine with significant anticholinergic effects) contraindicated in acute-angled glaucoma	Moderate
	(3) Adverse drug reaction: Patient with frequent urinary tract infection but continued on dapaglifozin, an SGLT-2 inhibitor	Moderate
11	(1) Duplication of therapy: Patient prescribed with PO prednisolone and IV Methylprednisolone (high-intensity) concurrently	Minor
	(2) Inappropriate dosage regimen: Levothyroxine prescribed in MG instead of MCG	Serious
12	(1) Drug drug interaction: Significant interaction between tramadol and linezolid leading to increased risk for serotonin syndrome	Moderate
	(2) Duplication of therapy: Patient prescribed with IV Tienam and PO ciprofloxacin for gram-negative coverage of urinary tract infection	Minor

	(3) Inappropriate dosage regimen: Instructions for prednisolone eye drops was to administer to left eye, when the affected eye was the right	Serious
13	(1) Drug drug interaction: Significant (contraindicated) interaction between azathioprine and allopurinol leading to increased risk for neutropenia	Serious
	(2) Omission of therapy: Patient initiated on allopurinol but not on anti-inflammation therapy to avoid exacerbation of gout flare (e.g. colchicine or steroids)	Moderate
14	(1) Omission of therapy: Noted complains of no bowel movement for n-days but no laxatives ordered for patient with a significant history of liver cirrhosis	Moderate
	(2) Drug drug interaction: Significant interaction between Epclusa and omeprazole that will reduce absorption and efficacy of anti-viral agent	Moderate
	(3) Inappropriate dosage regimen: Dosage of paracetamol not adjusted in the presence of liver cirrhosis	Moderate
15	Control Case	NA
16	(1) Drug drug interaction: Significant interaction between calcium supplement and tetracycline. Calcium reduces absorption of tetracycline when taken concurrently	Moderate
	(2) Inappropriate dosage regimen: Dose of metronidazole not adjusted in the presence of significant liver impairment	Moderate
17	(1) Inappropriate dosage regiment: The infusion rate of vancomycin has exceeded the maximum recommended rate, leading to increased risk for red man's syndrome	Serious
	(2) No indication: G-CSF (filgrastim) continued despite recovery of absolute neutrophil counts	Moderate
18	(1) Drug drug interaction: Significant interaction between ciclosporin and atorvastatin, with max dose of atorvastatin limited to not more than 20mg per day	Moderate
	(2) Inappropriate dosage regimen: Inappropriate dose of co-trimoxazole, prescribed in trimethoprim component instead of co-trimoxazole for PCP prophylaxis	Moderate
19	(1) Inappropriate dosage regimen: Dose of tramadol not adjusted in the presence of significant renal failure	Moderate
	(1) Inappropriate dosage regimen: Dose of gabapentin not adjusted in the presence of significant renal failure	Moderate
	(2) Omission of therapy: Aspirin omitted in patient with significant cardiac history and low risk of bleeding	Minor
	(3) Drug drug interaction: significant interaction between sulphonylurea and ciprofloxacin leading to increased risk for hypoglycemia	Minor
20	(1) Duplication of therapy: Celecoxib and etoricoxib overlapping mechanism of action	Minor
	(2) Duplication of therapy: PO and IV omeprazole both ordered	No harm
	(3) Adverse drug reaction: Initiation of hydralazine in patient with a background of lupus disease	Serious
21	(1) Adverse Drug Reaction: Hyperkalemia but continued on potassium chloride infusion	Serious
	(2) Inappropriate dosage regimen: Exceeded maximum recommended dose of omeprazole for the indication of ulcer prevention	Minor
	(3) Drug drug interaction: Significant interaction between prochlorperazine and metoclopramide	Serious
	(4) Omission of therapy: Untreated hyperlipidemia	Minor
	(5) Omission of therapy: Untreated hyperkalemia	Serious
22	(1) Inappropriate dosage regimen: Rapid intravenous infusion of potassium chloride 10mmol over 1 minute	Moderate
	(2) Omission of therapy: Omission of steroid (e.g. prednisolone) when on abiraterone treatment for castrate resistant bladder cancer	Moderate
	(3) Adverse drug reaction: Diclofenac use in patient with recent myocardial infarction presenting with heightened risk for cardiovascular events	Serious
23	(1) Duplication of therapy: Prescription of 2 alpha-blockers (Tamsulosin and Alfuzosin) in a patient with history of benign prostate hyperplasia	Minor
	(2) Wrong choice of therapy: Co-amoxiclav use in patient with wound culture growing *E.Coli* reported to be resistant to co-amoxiclav	Moderate
24	(1) Adverse drug reaction: Trimetazidine MR contraindicated in renal failure	Moderate
	(2) Adverse drug reaction: Digoxin continued despite renal failure and high digoxin levels	Serious
25	(1) Drug drug interaction: Tramadol contraindicated in SSRI overdose	Serious
	(2) Allergic reaction: Ketoprofen prescribed in patient with ibuprofen allergy	Serious

26	(1) Adverse drug reaction: Empaglifozin not held off in patient with infection and possible bleeding	Moderate
	(2) Duplication of therapy: Linaglpitin and sitagliptin both DPP-IV Inhibitors prescribed together	Moderate
27	(1) Duplication of Therapy: Patient is on neurobion and pyridoxine	Minor
	(2) Inappropriate dosage regimen: Antihypertensives regimen to be streamlined	Moderate
28	(1) Adverse drug reaction: Statin therapy in patient with elevated liver function test (ALT/AST > 3x ULN) from cholangiits and shock	Serious
	(2) Omission of therapy: No sliding scale insulin in hospitalized patient with elevated glucose level	Moderate
29	(1) Adverse drug reaction: IV Iron therapy continued in patient with active infection	Moderate
	(2) Adverse drug reaction: Azithromycin prescribed in patient with history of long QT syndrome	Serious
30	(1) Inappropriate drug selection: Novorapid (short-acting insulin) ordered as novomix, prescribed three times a day	Serious
31	(1) Monitoring and tests: IgA antibody titres not checked before Intragam infusion	Serious
	(2) Drug Interaction: Tramadol and dextromethorphan and risk for serotonin syndrome	Moderate
	(3) No indication for drug: sodium bicarbonate in high bicarbonate levels	Moderate
32	(1) Adverse drug reaction: Nifedipine immediate release intead of long acting formulation prescribed in 78 year old patient with hypertensive emergency.	Moderate
	(2) Inappropriate dosage regimen: Sitagliptin dose not adjusted in CKD	Moderate
33	(1) Inappropriate dosage regimen: Antibiotic doses (Piperacillin-Tazobactam and Vancomycin) not adjusted after termination of contious renal replacement therapy	Moderate
	(2) Adverse drug reaction: Magnesium sulfate injection given bolus for hypomagnesemia	Serious
34	(1) Drug Drug Interactions: Patient on warfarin prescribed with metronidazole without adjustments to warfarin dosage or closer INR monitoring	Serious
35	(1) Drug Drug interaction: Patient on posaconazole suspension for chronic suppression started on high dose IV omeprazole	Moderate
	(2) Inappropriate dosage regimen: Dose of potassium chloride too low for potassium replacement in adult patient with K level of 3.1 mmol/L	Moderate
36	(1) Drug Allergy: Patient with DRESS from esomeprazole, prescribed with omeprazole	Serious
	(2) Adverse Drug Reaction: Continued on aspirin and clopidogrel during immediate post EVT phase	Serious
37	(1) No indication for drug: Zirconium Cyclosilicate prescribed for pseudohyperkalemia (hemolysed blood sample)	Moderate
	(2) Drug Drug Interaction: Clarithromcyin (prescribed for ?otitis media) in patient starting on IV vincristine as part of chemotherapy for lymphoma treatment	Moderate
38	(1) Inappropriate dosage regimen: 60kg Female patient presenting with hypervolemic hyponatremia, metabolic acidosis and acute kidney injury started on a 1 litre isotonic bicarbonate infusion.	Moderate
	(2) Omission of therapy: Background of hypertension and hyperlipidemia, medication reconciliation not performed and chronic medications not resumed.	Minor
39	(1) Inappropriate dosage regimen: 50kg patient prescribed with desmopressin at 1mcg (dose is too low) for prevention of uremic bleeding prior to line insertion	Moderate
40	Control	NA

Table S3: Results from reasoning model (OpenAI's o4-mini)

Model	Accuracy (SD)	Precision (SD)	Recall (SD)	F1 Score (SD)
o4-mini Native	72.5% (0.63)	0.39 (0.02)	0.73 (0.01)	0.51 (0.02)
o4-mini RAG	73.6% (0.28)	0.37 (0.02)	0.71 (0.03)	0.49 (0.02)

SD: Standard Deviation

Data S1: Summary of Case Vignettes

Case No	Discipline	Brief Description of Clinical Vignettes	Number of Medications Prescribed	ATC Categories
1	Cardiology	61-year-old Malay male with history of diabetes, hypertension, chronic kidney disease, and recurrent vascular thrombotic events. He has undergone multiple surgeries, including amputations and thrombolysis. He presented with chest pain and was diagnosed with an evolved anterior myocardial infarction, for which he underwent successful percutaneous coronary intervention (PCI).	13	Electrolytes, Antithrombotics (Heparins), Insulin (fast-acting; rapid-acting and long-acting), Platelet aggregation inhibitors, proton pump inhibitors, Vasodilators, DPP-IV inhibitors, Beta-blocking agent (selective), ACE-inhibitors, B vitamins
2	Cardiology / Gastroenterology	41-year-old Malay male with a history of coronary artery disease, diabetes mellitus, KDIGO stage 3 kidney disease, and a recent episode of acute gout. He presented with epigastric pain and was admitted to hospital from the clinic. Investigations included coronary angiography with a view for percutaneous coronary intervention and gastrointestinal scopes. His medication allergies include penicillin.	13	Fast-acting insulins, parenteral nutritional products, blood glucose-lowering drugs, oral antidiabetics, lipid-modifying agents, sulfonylureas, beta-blocking agents, agents for gout, macrolides, nitroimidazole derivatives, proton pump inhibitors, antithrombotic agents.
3	Cardiology	66-year-old male with a history of minor coronary artery disease, hypertension, hyperlipidaemia, diabetes, and a chronic left caudate nucleus infarct. He presents with left lower limb swelling and pain, fever, and chest tightness. Diagnosed with an evolved inferior STEMI and left lower limb cellulitis complicated by acute kidney injury.	15	Long Acting insulin, Heparin group, low molecular weight heparins, Macrolides, Platelet aggregation inhibitors excl. heparin, Angiotensin-converting enzyme inhibitors, Sulfonylureas, Biguanides, Dipeptidyl peptidase 4 (DPP-4) inhibitors, HMG CoA reductase inhibitors, Beta-blocking agents, selective, Dihydropyridine derivatives, Proton pump inhibitors, Organic nitrates, Drugs used in erectile dysfunction
4	Cardiology	75-year-old Chinese male with a history of hypertension, hyperlipidaemia, benign prostatic hyperplasia, and a right occipital parasagittal meningioma. He presents with atypical chest pain, which is a dull ache and non-radiating. He has a history of ischemic cardiomyopathy and has undergone coronary artery bypass graft surgery and mitral valve repair.	6	Beta-blocking agents, selective, Platelet aggregation inhibitors excl. heparin, 5-alpha-reductase inhibitors, proton pump inhibitors, Alpha-adrenoreceptor antagonists, HMG CoA reductase inhibitors
5	Cardiology / Respiratory	58-year-old female admitted for acute pulmonary edema. Her past medical history includes left ataxic hemiparesis, poorly controlled diabetes mellitus, and hypertension. She exhibits shortness of breath, lower limb swelling, and is found to have an NSTEMI with underlying chronic type 2 respiratory failure likely contributed by obstructive sleep apnea.	16	Insulins and analogues, Organic nitrates, High-ceiling diuretics, Heparin group, low molecular weight heparins, Angiotensin II antagonists, Beta-blocking agents, selective, Sulfonylureas, Dipeptidyl peptidase 4 (DPP-4) inhibitors, Platelet aggregation inhibitors excl. heparin, Proton pump inhibitors
6	General Medicine	58-year-old male with a history of hypertension, admitted with dizziness and vertiginous symptoms, associated with difficulty balancing. He has a strong smoking history and had a possible posterior circulation stroke. The patient also shows signs of hyponatremia, high anion gap metabolic acidosis likely from fasting ketoacidosis, and possible polycythemia related to his smoking history.	7	Angiotensin-converting enzyme inhibitors, Platelet aggregation inhibitors excl. heparin, HMG CoA reductase inhibitors, Combination of penicillins, including beta-lactamase inhibitors
7	Endocrinology	65-year-old Indian female with a history of type 2 diabetes mellitus, hyperlipidemia, hypertension, osteoarthritis, and a past surgical procedure for a distal radius fracture. She was recently admitted for Group B streptococcus bacteremia secondary to pneumonia and experienced septic shock. She presented with an unwitnessed loss of consciousness and was	10	Angiotensin-converting enzyme inhibitors, Other antiepileptics), Sulfonylureas, Biguanides, HMG CoA reductase inhibitors, High-ceiling diuretics, Acetic acid derivatives and related substances

		found to have hypoglycemia and acute kidney injury on admission		
8	Endocrinology	68-year-old Chinese female with hypertension and osteoarthritis. She was previously admitted for severe symptomatic hypercalcemia with inappropriately normal parathyroid hormone levels, acute kidney injury, urinary tract infection, hypertensive urgency, anemia, and bilateral lower limb weakness due to cervical myelopathy. She is currently admitted from clinic for hypercalcemia management.	11	Calcitonins, Propulsives, Angiotensin II antagonists,Anilides, Antivertigo preparations, Osmotically acting laxatives, Vitamin D and analogues, Dihydropyridine derivatives
9	Endocrinology / Infectious disease / Vascular	71-year-old Chinese male with a complex medical history including ischemic heart disease, poorly controlled type 2 diabetes mellitus, peripheral vascular disease, chronic kidney disease stage 4, and moderate-severe dementia. He has been admitted multiple times for fluid overload and is currently admitted for the same issue. The patient also has infected lower limb diabetic ulcers and a painful indwelling catheter.	10	Insulins and analogues, High-ceiling diuretics, Biguanides, HMG CoA reductase inhibitors, Platelet aggregation inhibitors excl. heparin, H2-receptor antagonists, Contact laxatives
10	Ophthalmology	Female patient with right phacomorphic glaucoma and pseudophakia. She has diabetes mellitus, hypertension, hyperlipidemia, and no known drug allergies. There is a history of cataract and glaucoma, with no familial history of glaucoma.	16	Carbonic anhydrase inhibitors, HMG CoA reductase inhibitors, Alpha-adrenoreceptor agonists, Angiotensin-converting enzyme inhibitors, Sulfonylureas, Anxiolytics, Prostaglandin analogues, Sodium-glucose co-transporter 2 (SGLT2) inhibitors, Biguanides, Aldosterone antagonists)
11	Ophthalmology / Endocrinology	65-year-old female with severe thyroid eye disease (TED) related to Graves' disease. She has undergone total thyroidectomy and bilateral orbital decompression. Her current admission is for postoperative management of TED.	15	Combination of penicillins, including beta-lactamase inhibitors, Glucocorticoids, Anilides, HMG CoA reductase inhibitors, Selective COX-2 inhibitors, Thyroid hormones, Proton pump inhibitors, Aminoglycoside antibiotics
12	Ophthalmology / Infectious Disease	45-year-old male with no significant past medical history. He presented with right eye endophthalmitis and underwent various treatments, including intravitreal vancomycin and washout procedures. The patient was diagnosed with mycobacterium abscess and was treated with a combination of intravenous and oral medications.	12	Carbapenems, Macrolides, Quinolone, Other antibacterials, Anilides, Glucocorticoids, Other opioids
13	Gastroenterology	20-year-old female with primary sclerosing cholangitis and Child's Pugh A cirrhosis, chronic ulcerative pancolitis, and a history of cholelithiasis. She presented with left knee pain, fever, and symptoms of a respiratory infection. The preliminary assessment suggests a likely gout flare.	10	Immunosuppressants, Bile acids and derivatives, Proton pump inhibitors, Aminosalicylic acid and similar agents, Alpha and beta blocking agents, Quinolone antibacterials, Aminopyrazoles, Imidazole derivatives, Monobactams, Combination of penicillins, including beta-lactamase inhibitors
14	Gastroenterology	45-year-old Malay male with a history of Hepatitis C genotype 3A and Child’s B cirrhosis. He was admitted for deranged liver function tests and presented with jaundice. He presented with a needle stick injury and agrees to start treatment with Epclusa for Hepatitis C. Other active issues also includes managing possible acute cholecystitis and fluid restriction	6	High-ceiling diuretics, proton pump inhibitors, Aldosterone antagonists,Anilides<br>*Epclusa not listed
15	Gastroenterology	64-year-old Indian male with a history of Child's B8 Cirrhosis, diabetes, hypertension, hyperlipidemia, ischemic heart disease, atrial fibrillation, stage 3 chronic kidney disease, and hypothyroidism. He presents with worsening abdominal distension, lower limb edema, and reduced urine output. The patient has a history of spontaneous bacterial peritonitis and is on lifelong Ciprofloxacin for SBP prophylaxis.	10	Organic nitrates, Dipeptidyl peptidase 4 (DPP-4) inhibitors, Aminopyrazoles, Proton pump inhibitors, Platelet aggregation inhibitors excl. heparin, Alpha and beta blocking agents, Quinolone antibacterials, Cardiac glycosides, Thyroid hormones

16	Gastroenterology	77-year-old female with a history of Child's C10 cryptogenic liver cirrhosis with portal vein hypertension. She presented with diuretic-resistant ascites s/p ascitic drain with H. pylori infection.	8	Glycopeptide antibacterials, Antibiotics, Imidazole derivatives, Proton pump inhibitors, Tetracyclines, Osmotically acting laxatives, Quinolone antibacterials, Dihydropyridine derivatives)
17	General Surgery / Oncology	55-year-old Chinese male with a history of perforated duodenal ulcer, epithelial carcinoma of the salivary gland with liver metastasis, and neutropenia sepsis. His current condition includes postoperative care following laparoscopic omental patch repair of a perforated duodenal ulcer and chemotherapy for his cancer.	10	Insulins and analogues, Echinocandins, Colony-stimulating factors, Proton pump inhibitors, Anilides, Combination of penicillins, including beta-lactamase inhibitors, Other opioids, Glycopeptide antibacterials, Nucleoside and nucleotide reverse transcriptase inhibitors
18	Vascular Surgery	71-year-old Malay female with a complex medical history including end stage renal failure post living donor kidney transplant, complicated by chronic kidney disease of allograft, left renal cell carcinoma post radical nephrectomy, hyperparathyroidism with hypercalcemia, hypertension, type 2 diabetes mellitus, benign prostatic hyperplasia, and high cholesterol. She presents with right big toe pain and duskiness, leading to a diagnosis of dry gangrene of the right first toe.	18	Insulins and analogues, Beta-blocking agents, selective, HMG CoA reductase inhibitors, Immunosuppressants, Other calcimimetics, Platelet aggregation inhibitors excl. heparin, Trimethoprim and derivatives, combinations with sulfamethoxazole, Other lipid modifying agents, Folic acid, High-ceiling diuretics, Sulfonylureas, Dipeptidyl peptidase 4 (DPP-4) inhibitors, Angiotensin-converting enzyme inhibitors, Magnesium, Mycophenolic acid, proton pump inhibitors, Alpha-adrenoreceptor antagonists)
19	General Surgery / Colorectal Surgery	60-year-old Chinese male with a history of diabetes, hypertension, hyperlipidemia, polycythemia rubra vera, end-stage renal failure on hemodialysis, diverticular disease, and past surgery for perforated jejunal diverticulitis. He presented with lower abdominal pain and constipation suspicious for intestinal obstruction and later developed anal pain.	17	Osmotically acting laxatives, Anilides, Contact laxatives, Local anesthetics, Other opioids, HMG CoA reductase inhibitors, Other antiepileptics, Dipeptidyl peptidase 4 (DPP-4) inhibitors, Sulfonylureas, Propulsives, Other calcimimetics, Vasodilators used in peripheral vascular diseases, Phosphate binders, Organic nitrates, Quinolone antibacterials
20	General Surgery	46-year-old male with a history of adjustment disorder, lupus nephritis, hypertensive urgency, and chest pain. He presented with right lower abdominal pain associated with fever, diagnosed as perforated appendicitis. He underwent a laparotomy converted to open limited right hemicolectomy.	12	Potassium, Third -generation cephalosporins, Imidazole derivatives, Proton pump inhibitors, Serotonin (5HT3) antagonists, Propulsives, Anilides, Dihydropyridine derivatives, Angiotensin-converting enzyme inhibitors, Non-steroidal anti-inflammatory and antirheumatic products, coxibs, Hydralazine and diuretics, Selective COX-2 inhibitors
21	General Surgery	71-year-old male with a diagnosis of cecal diverticulitis and a small diverticular abscess. His past medical history includes benign prostatic hyperplasia, hypertension, and hyperlipidemia. The patient presented with right iliac fossa pain and had a history of abdominal pain.	11	Potassium, Third-generation cephalosporins, Imidazole derivatives, Proton pump inhibitors, Propulsives, 5-alpha-reductase inhibitors, Alpha-adrenoreceptor antagonists, Anilides, Other opioids, Sulfonamides, plain, Phenothiazines with aliphatic side-chain
22	Urology	75-year-old male with a history of ischemic colitis, hemorrhoids, benign prostatic hyperplasia (BPH), asthma, hypertension, gastritis, ischemic cardiomyopathy, and prostate cancer. He was admitted for gross hematuria and underwent bladder cystoscopy	14	Potassium, Combination of penicillins, including beta-lactamase inhibitors, Other antineoplastic agents, Platelet aggregation inhibitors excl. heparin, Beta-blocking agents,

		and cystodiathermy. His current issues include gross hematuria likely from prostate cancer and a urinary tract infection		selective, 5-alpha-reductase inhibitors, Anilides, Alpha-adrenoreceptor antagonists, Acetic acid derivatives and related substances, Organic nitrates, High-ceiling diuretics, Osmotically acting laxatives, HMG CoA reductase inhibitors
23	Urology	67-year-old male with a history of hypertension, type 2 diabetes mellitus, ischemic heart disease, benign prostatic hyperplasia, and pyelonephritis. He was admitted for pyelonephritis with persistent purulent discharge from the left loin and underwent percutaneous drainage.	12	Insulins and analogues, Platelet aggregation inhibitors excl. heparin, Beta-blocking agents, selective, Combination of penicillins, including beta-lactamase inhibitors, Other antiepileptics, Osmotically acting laxatives, Anilides, Other opioids, Alpha-adrenoreceptor antagonists,
24	Cardiology	80-year-old Chinese female with a significant history of cardiac issues, including severe cardiomyopathy and coronary artery disease, admitted with symptoms indicative of a new ST-elevation myocardial infarction (STEMI). She also has complications like acute renal failure and metabolic acidosis likely stemming from her cardiac condition and recent medical interventions.	13	Electrolytes, Antithrombotic agents, Platelet aggregation inhibitors, Proton pump inhibitors, Vasodilators used in cardiac diseases, Other vasodilators used in cardiac diseases, Cardiac therapy, Vasodilators for systemic use, Lipid modifying agents, plain, Beta blocking agents, plain, ACE inhibitors, plain, Vitamin B1, plain, and combination with Vitamin B6 and B12.
25	Emergency Medicine	17-year-old female who intentionally overdosed on over 20 fluvoxamine tablets due to emotional distress, witnessed by her family. Following the incident, she was brought to the emergency department, where she was alert, conversant, and stable, although she vomited once without expelling the pills. She expressed persistent harmful thoughts but no intent to act on them and requested psychological support. Her vital signs remained within normal limits, and her neurological exam showed no abnormalities. She was diagnosed with a fluvoxamine overdose and admitted to the ICU for monitoring, supported by telemetry and a consultation with a toxicologist.	5	Anesthetics, general, Electrolytes, Emollients and protectives, Non-steroidal anti-inflammatory drugs for topical use, Opioids
26	Endocrinology	79-year-old Chinese female with significant medical history including type 2 diabetes with complications, hypertension, hyperlipidemia, stage 3 chronic kidney disease, right knee replacement, hypothyroidism, and atrial fibrillation. She presented with severe right knee pain and swelling following a recent fall, leading to mobility issues. Lab results highlighted elevated INR and HbA1c, with clinical findings suggesting haemarthrosis exacerbated by warfarin use. The treatment plan includes fluid restriction, warfarin suspension, antibiotic administration to exclude septic arthritis, and potential knee joint aspiration.	18	Vitamin K, Proton pump inhibitors, High-ceiling diuretics, Beta-lactam antibacterials, monobactams, Glycopeptides, Blood glucose lowering drugs, insulins and analogues, Angiotensin II antagonists, plain, Antithrombotic agents, Sulfonylureas, Dipeptidyl peptidase-4 (DPP-4) inhibitors, Sodium-glucose co-transporter 2 (SGLT2) inhibitors, Lipid modifying agents, plain, Beta blocking agents, plain, Calcium channel blockers, Platelet aggregation inhibitors, Analgesics, non-opioid, Dipeptidyl peptidase-4 (DPP-4) inhibitors.
27	Family Medicine	Patient with past medical history that includes atrial fibrillation, incipient diabetic nephropathy, type II diabetes, hyperlipidemia, hypertension, and folate and vitamin B12 deficiencies. Recently hospitalized due to a urinary tract infection. Claims compliant to medications and report no side effects. The current plan is to continue with the existing medications and monitor diabetes management closely.	12	Emollients and protectives, Calcium channel blockers, ACE inhibitors, plain, Low-ceiling diuretics, thiazides, Vasodilators used in cardiac diseases, Lipid modifying agents, plain, Dipeptidyl peptidase-4 (DPP-4) inhibitors, Antithrombotic agents, direct factor Xa inhibitors, vitamins and minerals

28	Gastroenterology	80-year-old Chinese female patient with a background of hypertension, hyperlipidemia, and impaired glucose tolerance who presented with three weeks of vomiting, diarrhea, and abdominal pain, leading to hypotension requiring noradrenaline support. The patient is currently in the high dependency unit still complaining of abdominal pain. Labs show significantly deranged liver enzymes and bilirubin levels, while a CT scan revealed a 7mm distal common bile duct calculus causing duct dilation and signs of cholangitis. The primary diagnosis is Tokyo Grade III acute cholangitis due to choledocholithiasis and septic shock.	9	Prokinetic agents, Analgesics, non-opioid, Other beta-lactam antibacterials, Antibacterials for systemic use, Proton pump inhibitors, Vasopressors, Potassium-sparing agents in combination, Analgesics, non-opioid and muscle relaxants, Lipid modifying agents, plain.
29	Gastroenterology	75-year-old female with a history of type 2 diabetes, hypertension, peripheral vascular disease, end-stage renal failure on hemodialysis, long QT syndrome, and atrial fibrillation, currently not on anticoagulation due to bleeding risks. She was electively admitted for an ERCP to address an incidental common bile duct stone. Additionally, she has been experiencing a productive cough for two weeks, diagnosed as community-acquired pneumonia via a recent chest X-ray showing lower zone consolidation.	16	Antianemic preparations, Iron preparations, Antibacterials for systemic use, Other beta-lactam antibacterials, Proton pump inhibitors, Electrolytes, Analgesics, non-opioid, Opioids, Dipeptidyl peptidase-4 (DPP-4) inhibitors, Prokinetic agents, Lipid modifying agents, plain, Platelet aggregation inhibitors, Vitamin D and analog, Multivitamins with minerals, Laxatives, Phosphate binders
30	General Surgery	86-year-old Chinese female with a notable history, including a subtotal gastrectomy performed in January 2015 for a poorly differentiated adenocarcinoma of the stomach (pT3N1M0), gout treated with allopurinol, bilateral osteoarthritis of the knees, and diabetes managed with NovoMix 30. Recent evaluations via OGD (esophagogastroduodenoscopy) in June showed no signs of cancer recurrence. The patient reports a good appetite, tolerates solid foods well, and maintains energy with small, frequent meals. She is asymptomatic with an unremarkable physical examination and a pain score of zero.	9	Blood glucose lowering drugs, insulins and analogues, Proton pump inhibitors, Platelet aggregation inhibitors, Blood glucose lowering drugs excluding insulins, Blood glucose lowering drugs, SGLT2 inhibitors, Vitamin D and analogues, Agents for gout attacks.
31	Haematology	68-year-old male with a complex medical history including idiopathic thrombocytopenic purpura (ITP), ischemic heart disease with previous percutaneous coronary interventions, cerebrovascular accidents and transient ischemic attacks, chronic kidney disease stage 5 pending dialysis, hypertension, hyperlipidemia, and diabetes. He presented with recent haemoptysis and a noted petechial rash on his arms and chest, attributed to thrombocytopenia from ITP. Previously, his ITP was managed with steroids which were ceased due to induced diabetes, and currently, his response to eltrombopag is poor; however, he has shown good response to IV immunoglobulin (IVIg).	16	Antianemic preparations, Blood glucose lowering drugs, insulins and analogues, Antifibrinolytics, Corticosteroids for systemic use, Immune sera and immunoglobulins, Vasodilators used in cardiac diseases, Calcium channel blockers, Beta blocking agents, plain, Lipid modifying agents, plain, Vitamin D and analogues, Phosphate binders, Cough suppressants (excluding combinations with expectorants), Analgesics, non-opioid, Opioids, Antacids, Laxatives.
32	General Medicine	78-year-old woman with a medical history of diabetes, hypertension, glaucoma, and ischemic heart disease presented with non-vertiginous dizziness and hypertension (SBP in the 200s) after three episodes of vomiting that resolved upon hospital admission. She is alert and non-toxic, with a blood pressure of 185/95 and oxygen saturation of 99% on room air. Physical and neurological exams were unremarkable, and lab tests indicated hypokalemia and elevated creatinine levels. A CT brain scan and chest X-ray showed no acute issues. Her management includes close monitoring, dietary adjustments, potassium chloride replacement, and blood pressure control with nifedipine.	13	Electrolytes, Analgesics, non-opioid, Prokinetic agents, Mineral supplements, Calcium channel blockers, Blood glucose lowering drugs excluding insulins, Dipeptidyl peptidase-4 (DPP-4) inhibitors, Platelet aggregation inhibitors, Proton pump inhibitors, Lipid modifying agents, plain, Beta blocking agents, plain, Vitamin D and analogues.

33	General Medicine / Critical Care	65-year-old Indian male with a medical history of Child's A liver cirrhosis due to chronic hepatitis B, post-pyloric ulcer surgery, and recent right knee septic arthritis, is currently managing complications including fluid overload-related acute decompensated type 2 respiratory failure and KDIGO 3 acute kidney injury requiring dialysis. Admitted following septic shock with episodes of hypotension, he was stabilized in the medical intensive care unit with inotropic support and non-invasive ventilation.	15	Electrolytes, Beta-lactam antibacterials, including penicillins, Mineral supplements, Mineral supplements, Glycopeptides, Corticosteroids for systemic use, High-ceiling diuretics, Nucleoside and nucleotide reverse transcriptase inhibitors, Proton pump inhibitors, Beta blocking agents, plain, Antithrombotic agents, direct factor Xa inhibitors, Sulfonamides, plain, Laxatives, Analgesics, non-opioid, Mucolytics.
34	General Medicine	72-year-old Chinese male with multiple chronic conditions including type 2 diabetes, hyperlipidemia, hypertension, osteoarthritis, chronic glomerulonephritis-induced CKD, and alcoholic liver cirrhosis presented with generalized myalgia and weakness due to an accidental double dosing of statins, leading to rhabdomyolysis. Additional complications include fluid overload and heart failure with a mid-range ejection fraction. His treatment has been adjusted to manage these conditions, with a suspension of statins, fibrates, and dapagliflozin to mitigate further complications. He is under close monitoring with regular vitals, intake/output charting, and medication adjustments to manage fluid overload and support liver and kidney functions.	14	Electrolytes, Proton pump inhibitors, Other beta-lactam antibacterials, Antibacterials for systemic use, High-ceiling diuretics, Potassium-sparing agents, Beta blocking agents, plain, Blood glucose lowering drugs, SGLT2 inhibitors, Sulfonylureas, Laxatives, Contact laxatives, Lipid modifying agents, plain, Lipid modifying agents, fibrates, Antithrombotic agents.
35	Infectious Disease	82-year-old Chinese female with a history of hypertension, hyperlipidemia, diabetes, right hip osteoarthritis, methicillin-sensitive Staphylococcus aureus (MSSA) bacteremia, and triple vessel disease currently presents with low hemoglobin and a gradual functional decline reported over the past year. She experiences intermittent fever spikes and lethargy, alongside a reduced exercise tolerance. The patient denies pain, cough, or shortness of breath and exhibits stable vital signs with poor dental hygiene and consistent weakness on the right side compared to the left, as noted by her son. Lab results indicate anemia with hemoglobin at 8.7 g/dL, mild hyponatremia, and subclinical hypothyroidism.	11	Electrolytes, Proton pump inhibitors, Platelet aggregation inhibitors, H2-receptor antagonists, Laxatives, Contact laxatives, Calcium channel blockers, Drugs for urinary frequency and incontinence, Iron preparations, Antifungals for systemic use, Mineral supplements.
36	Neurology	72-year-old male with a history of hypertension and recent stroke symptoms during a trip to Indonesia is being treated for an acute left middle cerebral artery (MCA) infarction confirmed by CT, showing a hyperdense clot and good collateral circulation. His current management includes endovascular therapy (EVT) and medication regimen including IV nicardipine for blood pressure control, and integrilin infusion to maintain vascular patency, with strict neurological monitoring. He was deemed unsuitable for TPA due to MRI findings.	7	Platelet aggregation inhibitors, Proton pump inhibitors, Electrolytes, Calcium channel blockers, Lipid modifying agents, plain, Platelet aggregation inhibitors, Platelet aggregation inhibitors.
37	Medical Oncology	70-year-old Chinese male with a history of chronic ischemic heart disease, type 2 diabetes with nephropathy, chronic kidney disease stage 3, gout, and a history of otitis media is currently being treated for newly diagnosed stage IV aggressive large B-cell lymphoma. The patient presents with left flank pain, scrotal swelling, significant weight loss, loss of appetite, and worsening shortness of breath on exertion, alongside a left-sided upper rib pain. His condition includes complications from lung cancer in a family member and a 40 pack-year	22	Blood glucose lowering drugs, insulins and analogues, Electrolytes, Antiemetics and antinauseants, Antineoplastic agents, Antineoplastic agents, Antineoplastic agents, Antineoplastic agents, Antineoplastic agents, Antihistamines for systemic use, Antineoplastic and immunomodulating agents, Antineoplastic agents, Colony

		smoking history. Diagnostic workups have identified a retroperitoneal mass and involvement of the adrenal gland and omentum.		stimulating factors, ACE inhibitors, plain, Blood glucose lowering drugs, SGLT2 inhibitors, Blood glucose lowering drugs excluding insulins, Agents for gout attacks, Lipid modifying agents, plain, Platelet aggregation inhibitors, Calcium channel blockers, H2-receptor antagonists, Mucolytics, Potassium-binding agents, Macrolides, Analgesics, non-opioid.
38	Renal	65-year-old white male with a complex medical history including hypertension, dyslipidemia, hepatic steatosis, renal cysts, and previous surgery for a gluteal lipomatous tumor is currently admitted with central chest discomfort, orthopnea, and worsening exertional dyspnea. His symptoms have been progressive over the past two weeks, and he has developed bilateral lower limb swelling and abdominal bloating, suggestive of fluid overload. Lab results indicate acute kidney injury (KDIGO 3), hypervolemic hyponatremia, thrombocytopenia, and cholestatic liver function tests. He is being managed with intravenous furosemide for fluid overload and tazocin for suspected infection.	7	High-ceiling diuretics, Electrolytes, Beta-lactam antibacterials, including penicillins, Proton pump inhibitors, Laxatives, Contact laxatives, Cough suppressants (excluding combinations with expectorants).
39	Respiratory Medicine	52-year-old Chinese female with systemic lupus erythematosus and end-stage renal failure underwent bilateral radical nephrectomy for multifocal renal cell carcinoma, complicated by retroperitoneal hematoma and pulseless electrical activity requiring tracheostomy. Post-operatively, she developed Stenotrophomonas maltophilia bacteremia and type 2 myocardial infarction. Current management includes nasojejunal feeding to reduce aspiration risk, intravenous ceftazidime for bacteremia, and monitoring for potential dialysis. She remains stable with ongoing hemoptysis and raised troponins but no acute ischemic changes on ECG.	13	Other beta-lactam antibacterials, including penicillins, Beta blocking agents, plain, Vasodilators for systemic use, Pituitary and hypothalamic hormones and analogues, Platelet aggregation inhibitors, Proton pump inhibitors, Angiotensin II antagonists, plain, Calcium channel blockers, Alpha-adrenoreceptor antagonists, Beta blocking agents, plain, Other antiepileptics, Sympathomimetics, Emollients and protectives.
40	Respiratory Medicine	61-year-old Caucasian female with chronic obstructive pulmonary disease (COPD), hyperlipidemia, and heart failure was admitted for cough, shortness of breath, vomiting, and lethargy, presenting with decompensated type 2 respiratory failure. Treatment included non-invasive ventilation, antibiotics (ceftazidime and levofloxacin), and steroids (IV hydrocortisone) to manage an exacerbation likely caused by a viral infection.	11	Corticosteroids for systemic use, Proton pump inhibitors, Beta-lactam antibacterials, including penicillins, Macrolides, Sympathomimetics, anticholinergics, Inhalants for obstructive airway diseases, Electrolytes, Antivirals for systemic use, Analgesics, non-opioid, Drugs for obstructive airway diseases, combinations, Lipid modifying agents, plain.
